# Efficient estimation for deep generalized accelerated hazards models with interval-censored data

**DOI:** 10.1093/biomtc/ujag140

**Published:** 2026-08-10

**Authors:** Qiang Wu, Mingyue Du, Shuangge Ma, Xingqiu Zhao

**Affiliations:** Department of Applied Mathematics, The Hong Kong Polytechnic University, Hong Kong 999077, China; School of Mathematics, Jilin University, Changchun, Jilin 130012, China; Department of Biostatistics, Yale University, New Haven, CT 06520, United States; Department of Applied Mathematics, The Hong Kong Polytechnic University, Hong Kong 999077, China

**Keywords:** deep neural networks, generalized accelerated hazards model, interval-censored data, nonasymptotic error bound, semiparametric efficiency

## Abstract

For the analysis of interval-censored data, we propose a deep generalized accelerated hazards model. This model is designed to facilitate a detailed exploration of the relationship between various risk factors and the hazard associated with failure time. We develop a sieve maximum likelihood estimation procedure that combines deep neural networks and monotonic splines. By employing deep neural networks, we can effectively capture nonparametric effects, enabling a flexible and adaptive modeling approach for complex relationships. Under certain regularity conditions, we derive a nonasymptotic error bound for the resulting estimator and show that the finite-dimensional estimator is asymptotically normal and achieves the semiparametric efficiency. We conduct simulation studies to evaluate the finite-sample performance of the proposed approach. Furthermore, the proposed method is applied to the Atherosclerosis Risk in Communities study for practical illustration.

## 1 Introduction

Recent years have seen significant growth in the analysis of failure time data, particularly interval-censored data, which frequently arises in clinical trials and longitudinal studies with periodic follow-ups (Finkelstein, 1986; Kalbfleisch and Prentice, 2002). Interval-censored data arises when the exact failure time is unobserved, known only to fall within an interval bounded by consecutive examinations. For example, in the Atherosclerosis Risk in Communities study (Zhou et al., 2017), participants were examined approximately every 3 years. Consequently, the exact onset time of diseases such as diabetes was unknown, yielding only interval-censored data bounded by 2 consecutive visits.

Regression models are widely used to assess covariate effects and predict survival probabilities. A foundational approach is the proportional hazards (PH) model (Cox, 1972), which assumes the conditional hazard function for survival time *T* given covariates $\boldsymbol{X}$ takes the form $\lambda (t|\boldsymbol{X})=\lambda (t) \exp (\boldsymbol{X}^\top {\boldsymbol{\beta }})$, where $\lambda (t)$ is an unknown baseline hazard and ${\boldsymbol{\beta }}$ represents the regression coefficients. To provide greater flexibility, various alternatives have been developed, including the accelerated failure time (AFT) and additive hazards models. Unifying these approaches, Chen and Jewell (2001) proposed an extended class of semiparametric hazards regression models (EHM) for right-censored data, encompassing the PH and AFT models as special cases. Further methodological advances include a sieve maximum likelihood estimation technique for the general accelerated hazards model (Zhao et al., 2017), and generalized accelerated hazards mixture cure models for interval-censored data (Liu and Xiang, 2021).

Recent applications of deep learning in survival analysis include partially linear Cox and deep extended hazards models for right-censored data (Zhong et al., 2021, 2022). These approaches utilize sparse DNNs and partial likelihoods, elegantly bypassing baseline hazard estimation during training. For interval-censored data, Sun and Ding (2023) approximated the baseline hazard using Bernstein polynomials in a deep nonparametric Cox model. Despite this progress, existing paradigms face critical bottlenecks: (1) sparse architectures often suffer from optimization instability and difficult parameter tuning; (2) partial likelihood approaches require a cumbersome two-step prediction process and are intractable for interval-censored data; (3) Bernstein polynomials exhibit numerical fragility and global distortion in dynamic time-scaling environments; and (4) theoretical guarantees under a full-likelihood framework remain fundamentally underexplored, particularly regarding the complex projection onto the joint tangent space of infinite-dimensional nuisance parameters for interval-censored data.

In this paper, we introduce a semiparametric modeling framework for interval-censored data that is sufficiently general to accommodate different goals. If the magnitude of a treatment effect must be interpretable, then we model the corresponding variable linearly and capture all remaining covariates jointly through a single nonparametric function; otherwise, for purely predictive goals, we adopt a fully nonparametric model in which all covariates enter jointly through a unified flexible function. Let *T* denote the survival time of interest. Suppose that we observe 2 sets of covariates, $\boldsymbol{Z}$ and $\boldsymbol{X}$. The vector $\boldsymbol{Z}\in \mathbb {R}^p$ collects the covariates of substantive interest, that is, the variables to be explained, including categorical predictors and potential treatment indicators. The vector $\boldsymbol{X}\in \mathbb {R}^r$ comprises the remaining covariates that are not the primary focus. Assume that given the covariates $(\boldsymbol{Z},\boldsymbol{X})\in \mathbb {R}^p\times \mathbb {R}^r$, the cumulative hazard function of *T* follows a generalized accelerated hazards model:


(1) \begin{eqnarray*}
\Lambda (t| \boldsymbol{Z}, \boldsymbol{X})=\Lambda \left(t \exp \left\lbrace {\boldsymbol{\beta }}^{\top }\boldsymbol{Z}+g_1(\boldsymbol{X})\right\rbrace \right)\exp \left\lbrace {\boldsymbol{\gamma }}^{\top }\boldsymbol{Z}+g_2(\boldsymbol{X})\right\rbrace ,
\end{eqnarray*}

where $\Lambda (\cdot )$ denotes an unknown baseline cumulative hazard function, ${\boldsymbol{\beta }}$ and ${\boldsymbol{\gamma }}$ are *p*-dimensional vectors of regression parameters, and both $g_1$ and $g_2$ are unknown *r*-variate functions. Hence, our model not only retains the straightforward interpretability of the finite-dimensional parameter in the Cox PH model but also incorporates more complex nonparametric effects of covariates. When interpretation of treatment effects on the hazard is unnecessary, the framework (1) simplifies to a fully nonparametric setting that models all covariates jointly (pooling $\boldsymbol{Z}$ and $\boldsymbol{X}$).

The main contributions of our work are summarized as follows:

(1) For interval-censored data, we introduce a flexible deep generalized accelerated hazards model (DGAHM), including the standard PH model (${\boldsymbol{\beta }}={\boldsymbol{0}}, g_1=g_2=0$), the standard AFT model (${\boldsymbol{\gamma }}={\boldsymbol{0}}, g_1=g_2=0$) as special cases. We further develop a unified estimation procedure for the DGAHM model (1).

(2) Standard gradient-based optimization methods are highly unstable due to the complex entanglement of the baseline cumulative hazard function and nonlinear covariate effects within the likelihood function. To address this, we propose a novel iterative optimization algorithm that effectively decouples these components during training.

(3) We provide a comprehensive theoretical foundation for our hybrid estimator. Specifically, we calculate a nonasymptotic error bound for the full-likelihood sieve estimator, prove the asymptotic normality of the finite-dimensional component, and demonstrate its semiparametric efficiency.

The remainder of this paper is organized as follows. In Section 2, we introduce the log-likelihood function for interval-censored data and outline the estimation procedure using fully connected neural networks and monotone splines. In Section 3, we focus on the theoretical properties of the proposed estimator. In Section 4, we give a detailed implementation of the computation, as well as the estimation method of the information bound. Section 5 presents the results obtained from a simulation study conducted to assess the performance of the proposed method in finite-sample scenarios. In Section 6, we apply the proposed method to the Atherosclerosis Risk in Communities study. Concluding remarks are given in Section 7.

## 2 Methodology

In the following, we focus on the situation where for each study subject, one only collects 2 observation times denoted by *U* and *V* with $U < V$ and censoring indicators $\Delta _{1} = I_ { \lbrace T \le U \rbrace }$, $\Delta _{2} = I_{ \lbrace U < T \le V \rbrace }$ and $\Delta _{3} = 1 - \Delta _{1} - \Delta _{2}$, where $I(\cdot )$ denotes the indicator function taking the value of 1 if the condition holds and 0 otherwise. Assume that the supports of *U* and *V* are intervals within $[0, \tau ]$, where $\tau$ is the end time of the study. Also assume that conditional on $\boldsymbol{Z}$ and $\boldsymbol{X}$, *T* is independent of $(U, V)$, the so-called conditionally independent censoring mechanism. Under the model (1), given the covariates $(\boldsymbol{Z}, \boldsymbol{X})$, one can express the conditional survival function of the failure time as $S(t|\boldsymbol{Z},\boldsymbol{X})=\exp \left\lbrace -\Lambda (t|\boldsymbol{Z},\boldsymbol{X})\right\rbrace .$ For the convenience of statistical inference, we denote $\phi = \log \Lambda .$ Then, the model (1) can be rewritten as


(2) \begin{eqnarray*}
\Lambda (t| \boldsymbol{Z}, \boldsymbol{X})=\exp \left\lbrace {\boldsymbol{\gamma }}^{\top } \boldsymbol{Z}+g_2\left(\boldsymbol{X}\right)+\phi \left(t \exp \left\lbrace {\boldsymbol{\beta }}^{\top } \boldsymbol{Z}+g_1\left(\boldsymbol{X}\right)\right\rbrace \right)\right\rbrace .
\end{eqnarray*}

The observed data consist of $\lbrace (\Delta _{1i}, \Delta _{2i}, U_i, V_i, \boldsymbol{Z}_i, \boldsymbol{X}_i): i = 1,2,\ldots ,n\rbrace$, which are independent and identically distributed copies of $(\Delta _1, \Delta _2, U, V, \boldsymbol{Z}, \boldsymbol{X})$. Under the model (2), the log-likelihood function is


(3) \begin{eqnarray*}
&&\ell _n\left({\boldsymbol{\beta }}, {\boldsymbol{\gamma }}, g_1, g_2, \phi \right)\\&&= \sum _{i=1}^n\left[\Delta _{1i} \log \left\lbrace 1-\exp \left[- \exp \left\lbrace {\boldsymbol{\gamma }}^{\top } \boldsymbol{Z}_i+g_2\left(\boldsymbol{X}_i\right)+\phi \left(U_i \exp \left\lbrace {\boldsymbol{\beta }}^{\top } \boldsymbol{Z}_i+g_1\left(\boldsymbol{X}_i\right)\right\rbrace \right)\right\rbrace \right]\right\rbrace \right.\\&&\qquad \quad +\Delta _{2 i} \log \left\lbrace \exp \left[- \exp \left\lbrace {\boldsymbol{\gamma }}^{\top } \boldsymbol{Z}_i+g_2\left(\boldsymbol{X}_i\right)+\phi \left(U_i \exp \left\lbrace {\boldsymbol{\beta }}^{\top } \boldsymbol{Z}_i+g_1\left(\boldsymbol{X}_i\right)\right\rbrace \right)\right\rbrace \right]\right.\\&&\qquad \quad \left.- \exp \left[- \exp \left\lbrace {\boldsymbol{\gamma }}^{\top } \boldsymbol{Z}_i+g_2\left(\boldsymbol{X}_i\right)+\phi \left(V_i \exp \left\lbrace {\boldsymbol{\beta }}^{\top } \boldsymbol{Z}_i+g_1\left(\boldsymbol{X}_i\right)\right\rbrace \right)\right\rbrace \right]\right\rbrace \\&&\left.\qquad \quad -(1-\Delta _{1i}-\Delta _{2i})\exp \left\lbrace {\boldsymbol{\gamma }}^{\top } \boldsymbol{Z}_i+g_2\left(\boldsymbol{X}_i\right)+\phi \left(V_i \exp \left\lbrace {\boldsymbol{\beta }}^{\top } \boldsymbol{Z}_i+g_1\left(\boldsymbol{X}_i\right)\right\rbrace \right)\right\rbrace \right].
\end{eqnarray*}

For the estimation in model (2), a natural approach is to maximize (3) concerning ${\boldsymbol{\beta }}$, ${\boldsymbol{\gamma }}$, $g_1$, $g_2$, and $\phi$. It becomes apparent that direct maximization of the objective function would be challenging or even infeasible due to the involvement of unknown functions $g_1$, $g_2$, and $\phi$. To overcome this difficulty, we propose to approximate the function $\phi$ using B-spline functions and the functions $g_1$ and $g_2$ applying DNNs. The choice of B-splines for $\phi$ strictly enforces the monotonicity of the baseline cumulative hazard function. Enforcing such shape constraints is straightforward with spline coefficients but computationally non-trivial with DNNs. Furthermore, since $\phi$ is a univariate function, B-splines offer a computationally efficient and parsimonious approximation, avoiding the severe risks of overparameterization and overfitting associated with applying a highly parameterized neural network to a one-dimensional input.

First, we discuss the approximation procedure of the function $\phi$. Denote


\begin{eqnarray*}
&& M_1=\inf _{U, \boldsymbol{Z}, \boldsymbol{X}}U \exp \lbrace {\boldsymbol{\beta }}_0^{\top } \boldsymbol{Z}+g_{10}(\boldsymbol{X})\rbrace \mbox{and} M_2=\sup _{V, \boldsymbol{Z}, \boldsymbol{X}}V \exp\\&& \lbrace {\boldsymbol{\beta }}_0^{\top } \boldsymbol{Z}+g_{10}(\boldsymbol{X})\rbrace .
\end{eqnarray*}

To approximate the function $\phi$ using B-spline functions on $[M_1, M_2]$, we start with a partition $M_1=d_0< d_1< \cdots < d_{k_n}< d_{k_n+1}=M_2$, which divides the interval $\left[M_1, M_2\right]$ into $k_n+1$ identical sub-intervals, where $k_n$ is a positive integer with $k_n=O\left(n^v\right)$ for $0< v< \frac{1}{2}$ and $\max _{1 \le j \le k_n+1}\left|d_j-d_{j-1}\right|=O\left(n^{-v}\right)$. Let $D_{k_n}=\lbrace d_1, d_2, \ldots , d_{k_n}\rbrace$ be the interior knot set of the splines. The spline space, denoted as $\mathcal {M}\left(D_{k_n}, m\right)$, can then be defined by


\begin{eqnarray*}
\mathcal {M}\left(D_{k_n}, m\right)=\left\lbrace \phi _n: \phi _n(t)=\boldsymbol{c}^{\top }\boldsymbol{B}_m(t)=\sum _{j=1}^{q_n} c_j B_{j,m}(t), \boldsymbol{c} \in \mathcal {C}_{q_n}\right\rbrace ,
\end{eqnarray*}

where *m* denotes the degree of the B-spline functions and $\boldsymbol{B}_m=(B_{1, m}, B_{2, m}, \ldots , B_{q_n, m})^{\top }$ is the vector of spline basis functions of degree *m*, and $q_n=m+k_n+1$ is the number of spline functions, and $\mathcal {C}_{q_n}=\left\lbrace \boldsymbol{c}=\left(c_1, \ldots , c_{q_n}\right)^{\top }: -\infty < -\mathcal {C} \le c_1 \le c_2 \le \cdots \le c_{q_n} \le \mathcal {C} < \infty \right\rbrace$ is the set of spline coefficients.

Second, we discuss the approximation to the functions $g_1$ and $g_2$. For this, we first briefly review the basic structure of DNNs. Let $\mathcal {L}$ be a positive integer and $\boldsymbol{p}=\left(p_{0}, \ldots , p_{\mathcal {L}}, p_{\mathcal {L}+1}\right)^{\top }$ be some positive integer sequence. A $(\mathcal {L}+1)$-layer DNN with layer-width $\boldsymbol{p}$ is a composite function $g: \mathbb {R}^{p_{0}} \rightarrow \mathbb {R}^{p_{\mathcal {L}+1}}$ recursively defined as


(4) \begin{eqnarray*}
& g(\boldsymbol{x})=W_{\mathcal {L}} g_{\mathcal {L}}(\boldsymbol{x})+v_{\mathcal {L}}, g_{\mathcal {L}}(\boldsymbol{x})=\sigma \left(W_{\mathcal {L}-1} g_{\mathcal {L}-1}(\boldsymbol{x})+v_{\mathcal {L}-1}\right),\\& \ldots , g_{1}(\boldsymbol{x})=\sigma \left(W_{0} \boldsymbol{x}+v_{0}\right).
\end{eqnarray*}

Here, the matrices $W_{k} \in \mathbb {R}^{p_{k+1} \times p_{k}}$ and vectors $v_{k} \in \mathbb {R}^{p_{k+1}}$ ($k=0, \ldots , \mathcal {L}$) are the parameters of this DNN *g*. We consider the neural networks with rectified linear unit activation functions in the following. For the DNN in (4), $\mathcal {L}$ denotes the depth of the network and vector $\boldsymbol{p}$ lists the width of each layer; particularly, $p_0=r$ denotes the dimension of the input layer and $p_{\mathcal {L}+1}=1$ is the dimension of the output layer. Let $\mathcal {W}$ denote the maximum width of hidden layers, that is, $\mathcal {W}=\max \lbrace p_1,\ldots ,p_{\mathcal {L}}\rbrace$. Note that the total number of parameters in (4) is $\sum _{j=0}^\mathcal {L}p_{j+1}(p_j+1),$ denoted as $\mathcal {S}$. In this paper, we consider a class of DNN functions:


\begin{eqnarray*}
\mathcal {G}_{\boldsymbol{p},\mathcal {L},\mathcal {B},\mathcal {W},\mathcal {S}}=\lbrace g: g \mbox{can be expressed as in} (4) \mbox{and} \Vert g\Vert _{\infty }\le \mathcal {B}\rbrace ,
\end{eqnarray*}

where $\Vert g\Vert _{\infty }$ is the sup-norm of the function *g*, and $0< \mathcal {B}< \infty$.

The network parameters can vary depending on the sample size *n*, so for simplicity, we define $\mathcal {G}_n=\mathcal {G}_{\boldsymbol{p},\mathcal {L},\mathcal {B},\mathcal {W},\mathcal {S}}.$ Let $\mathfrak {B}_1 \subset \mathbb {R}^p$ and $\mathfrak {B}_2 \subset \mathbb {R}^p$ denote the parameter spaces of ${\boldsymbol{\beta }}$ and ${\boldsymbol{\gamma }}$, respectively, and define $\mathcal {M}_n = \mathcal {M}\left(D_{k_n}, m\right)$. Let ${\boldsymbol{\eta }}=({\boldsymbol{\beta }}, {\boldsymbol{\gamma }}, g_1, g_2, \phi )$ and denote the true value of ${\boldsymbol{\eta }}$ by ${\boldsymbol{\eta }}_0=({\boldsymbol{\beta }}_0, {\boldsymbol{\gamma }}_0, g_{10}, g_{20}, \phi _0).$ Then, we estimate ${\boldsymbol{\eta }}_0$ by maximizing the log-likelihood function:


(5) \begin{eqnarray*}
&& \widehat{{\boldsymbol{\eta }}}_n=(\widehat{{\boldsymbol{\beta }}}_n,\widehat{{\boldsymbol{\gamma }}}_n, {\widehat{g}}_{1n}, {\widehat{g}}_{2n}, {\widehat{\phi }_n})=\mathop {\arg \max }_{\left({\boldsymbol{\beta }}, {\boldsymbol{\gamma }}, g_1, g_2, \phi \right)\in \mathfrak {B}_1\times \mathfrak {B}_2\times \mathcal {G}_n\times \mathcal {G}_n\times \mathcal {M}_n}\\&& \ell _n\left({\boldsymbol{\beta }}, {\boldsymbol{\gamma }}, g_1, g_2, \phi \right),
\end{eqnarray*}

where $\ell _n\left({\boldsymbol{\beta }}, {\boldsymbol{\gamma }}, g_1, g_2, \phi \right)$ is defined in (3).

## 3 Theoretical properties

### 3.1 Semiparametric properties

We first introduce some notations. For any vector, $v=(v_1,\ldots ,v_p)^\top \in \mathbb {R}^p$, $\Vert v\Vert =(\sum _{i=1}^pv_i^2)^{1/2}$, and $\Vert v\Vert _{\infty }=\max _i|v_i|$, and for any matrix, $W=(w_{ij})\in \mathbb {R}^{m\times n},$  $\Vert W\Vert _{\infty }=\max _{i,j}|w_{ij}|.$ For any function $f(x),$  $\Vert f\Vert _{\infty }=\sup _x|f(x)|$. In this paper, without loss of generality, we assume that the covariate vector $\boldsymbol{X}$ takes values in $[0,1]^r$. Denote $a_n \lesssim b_n$ if there exists a constant $c > 0$ such that $a_n \le c, b_n$. We write $a_n \asymp b_n$ when both $a_n \lesssim b_n$ and $b_n \lesssim a_n$ hold. Throughout the context, $\mathbb {P}_n$ and *P* denote the empirical and probability measures, respectively. For example, for a function $f:\mathcal {X}\rightarrow \mathbb {R}$, we write $\mathbb {P}_n(f):=\frac{1}{n} \sum _{i=1}^nf(X)$ and $P (f):=\mathbb {E}f(X).$ Denote $\Theta =\mathfrak {B}_1\times \mathfrak {B}_2\times \mathcal {G}_n\times \mathcal {G}_n\times \mathcal {M}_n$. For any ${\boldsymbol{\eta }}_1=({\boldsymbol{\beta }}_1, {\boldsymbol{\gamma }}_1, g_{11}, g_{21}, \phi _1)$ and ${\boldsymbol{\eta }}_2=({\boldsymbol{\beta }}_2, {\boldsymbol{\gamma }}_2, g_{12}, g_{22}, \phi _2)\in \Theta$, define


\begin{eqnarray*}
\begin{aligned} d\left({\boldsymbol{\eta }}_1,{\boldsymbol{\eta }}_2\right) =& \left\lbrace \Vert {\boldsymbol{\beta }}_1-{\boldsymbol{\beta }}_2 \Vert ^2+ \Vert {\boldsymbol{\gamma }}_1-{\boldsymbol{\gamma }}_2 \Vert ^2+\left\Vert g_{11}-g_{12}\right\Vert _{L^2\left([0,1]^r\right)}^2 \right. \\& \left.+\left\Vert g_{21}-g_{22}\right\Vert _{L^2\left([0,1]^r\right)}^2+\left\Vert \phi _1-\phi _2\right\Vert _{\Phi }^2\right\rbrace ^{1 / 2}, \end{aligned}
\end{eqnarray*}

where $\Vert g_{j1}-g_{j2}\Vert ^2_{L^2([0,1]^r)}=\mathbb {E}\lbrace g_{j1}(\boldsymbol{X})-g_{j2}(\boldsymbol{X})\rbrace ^2 (\mbox{for} j=1,2),$ and


\begin{eqnarray*}
\begin{aligned} \left\Vert \phi _1-\phi _2\right\Vert _{\Phi }^2=\mathbb {E}& \left[\left\lbrace \phi _1\left(U \exp \lbrace {\boldsymbol{\beta }}_0^{\top } \boldsymbol{Z}+g_{10}(\boldsymbol{X})\rbrace \right)-\phi _2\left(U \exp \lbrace {\boldsymbol{\beta }}_0^{\top } \boldsymbol{Z}+g_{10}(\boldsymbol{X})\rbrace \right)\right\rbrace ^2 \right.\\& \left.+\left\lbrace \phi _1\left(V \exp \lbrace {\boldsymbol{\beta }}_0^{\top } \boldsymbol{Z}+g_{10}(\boldsymbol{X})\rbrace \right)-\phi _2\left(V \exp \lbrace {\boldsymbol{\beta }}_0^{\top } \boldsymbol{Z}+g_{10}(\boldsymbol{X})\rbrace \right)\right\rbrace ^2\right]. \end{aligned}
\end{eqnarray*}

For a positive constant $B_0$, the Hölder class of smooth functions $\mathcal {H}^\xi ([0,1]^r, B_0)$ is defined as


(6) \begin{eqnarray*}
&&\mathcal {H}^\xi ([0,1]^r, B_0) \\&&=\left\lbrace g: [0,1]^r \rightarrow \mathbb {R}: \sum _{{\boldsymbol{\alpha }}:|{\boldsymbol{\alpha }}|< \xi }\left\Vert \partial ^{{\boldsymbol{\alpha }}} g\right\Vert _{\infty }+\sum _{{\boldsymbol{\alpha }}:|{\boldsymbol{\alpha }}|=\lfloor \xi \rfloor } \sup _{x \ne y} \frac{\left|\partial ^{{\boldsymbol{\alpha }}} g(x)-\partial ^{{\boldsymbol{\alpha }}} g(y)\right|}{\Vert x-y\Vert ^{\xi -\lfloor \xi \rfloor } }\le B_0\right\rbrace ,
\end{eqnarray*}

where $\lfloor \xi \rfloor$ denotes the largest integer strictly smaller than $\xi$, $\partial ^{\boldsymbol{\alpha }}:=\partial ^{\alpha _1} \cdots \partial ^{\alpha _r}$ with ${\boldsymbol{\alpha }}=\left(\alpha _1, \ldots , \alpha _r\right)^{\top }\in \mathbb {N}^r$, and $|{\boldsymbol{\alpha }}|=\sum _{k=1}^r \alpha _k$.

Our theoretical analysis needs the following regularity conditions:

(C1) The functions $g_{10}$ and $g_{20}$ belong to the Hölder class $\mathcal {H}^\xi ([0,1]^r, B_0)$ defined in (6).

(C2) The covariates $(\boldsymbol{Z}, \boldsymbol{X})$ take values in a bounded subset of $\mathbb {R}^{p+r}$. Without loss of generality, we assume $(\boldsymbol{Z}, \boldsymbol{X})\in [0,1]^{p+r}.$ And suppose that $\mathfrak {B}_1$ and $\mathfrak {B}_2$ are both compact and contain the true parameter ${\boldsymbol{\beta }}_0$ and ${\boldsymbol{\gamma }}_0$ as their interior points, respectively. Assume $\Vert {\boldsymbol{\beta }}\Vert _{\infty }< A$ and $\Vert {\boldsymbol{\gamma }}\Vert _{\infty }< A$ with *A* being a finite positive constant.

(C3) Denote $\Lambda _0$ as the true value of $\Lambda$, and let $\phi _0=\log \Lambda _0$. The $u^{\rm {th}}$ derivatives of $\phi _0$ strictly satisfy Lipschitz conditions with $u\ge 1$ and $\phi _0$ is strictly increasing on $[M_1, M_2].$

(C4) There exists a positive number $\kappa$ such that $P(V-U \ge \kappa )=1$, and $0< \Lambda _0(M_1)< \Lambda _0(M_2)< \infty$.

(C5) For any ${\boldsymbol{\beta }}\ne {\boldsymbol{\beta }}_0$ and ${\boldsymbol{\gamma }}\ne {\boldsymbol{\gamma }}_0$, $P\left({\boldsymbol{\beta }}^{\top } \boldsymbol{Z} \ne {\boldsymbol{\beta }}_0^{\top } \boldsymbol{Z}\right)> 0$ and $P\left({\boldsymbol{\gamma }}^{\top }\boldsymbol{Z} \ne {\boldsymbol{\gamma }}_0^{\top } \boldsymbol{Z}\right)> 0$. The nonparametric function $g_{10}$ and $g_{20}$ satisfy $\mathbb {E}g_{10}(\boldsymbol{X})=0$ and $\mathbb {E}g_{20}(\boldsymbol{X})=0$.

(C6) For some $\epsilon \in (0,1)$, $a^{\top } \operatorname{var}(\boldsymbol{Z}| U, \boldsymbol{X}) a \ge \epsilon a^{\top } \mathbb {E}(\boldsymbol{Z} \boldsymbol{Z}^{\top }| U, \boldsymbol{X}) a$ and $a^{\top } \operatorname{var}(\boldsymbol{Z}| V, \boldsymbol{X}) a \ge \epsilon a^{\top } \mathbb {E}(\boldsymbol{Z} \boldsymbol{Z}^{\top }| V, \boldsymbol{X}) a$ a.s. for all $a \in {\mathbb {R}}^p$.

(C7) For some $k> 1,$ the $k^{\rm {th}}$ partial derivatives of the joint density $f(\delta _1,\delta _2, u, v,\boldsymbol{z},\boldsymbol{x})$ of $(\Delta _1,\Delta _2,U, V, \boldsymbol{Z}, \boldsymbol{X})$ with respect to $U, V, \boldsymbol{Z},$ or $\boldsymbol{X}$ exist and are bounded.

Condition (C1) assumes the smoothness of $g_{10}$ and $g_{20}$, ensuring the existence of all partial derivatives up to the $\lfloor \xi \rfloor$ -th order, which is a technical assumption and is satisfied in many applications [see Assumption 2 of Jiao et al. (2023)]. Conditions (C2) and (C4) are standard assumptions in survival analysis. The smoothness and monotonicity assumptions of $\phi _0$ in condition (C3) guarantee that the spline approximation accurately captures the underlying behavior of $\phi _0$. Condition (C5) ensures that the model (1) or (2) is identifiable. By applying Markov’s inequality, it becomes apparent that condition (C6) implies that $\mathbb {E}(\boldsymbol{Z}\boldsymbol{Z}^{\top })$ is a positive definite matrix. Condition (C7) is required to establish the asymptotic normality of the estimator of $({\boldsymbol{\beta }}_0^\top ,{\boldsymbol{\gamma }}_0^\top )^\top$.

Denote $\boldsymbol{O}=(\Delta _1,\Delta _2,U,V,\boldsymbol{Z},\boldsymbol{X})$, and $\boldsymbol{S}=\lbrace \boldsymbol{O}_i=(\Delta _{1i},\Delta _{2i},U_i,V_i,\boldsymbol{Z}_i,\boldsymbol{X}_i)\rbrace _{i=1}^n$ as a random sample from the distribution of $\boldsymbol{O}$, and $\boldsymbol{S}^{\prime }=\lbrace \boldsymbol{O}_i^{\prime }=(\Delta _{1i}^{\prime },\Delta _{2i}^{\prime },U_i^{\prime },V_i^{\prime },\boldsymbol{Z}_i^{\prime },\boldsymbol{X}_i^{\prime })\rbrace _{i=1}^n$ is the sample independent of $\boldsymbol{S}$. Let $f_{{\boldsymbol{\eta }}}(\boldsymbol{Z},\boldsymbol{X},t)=\exp \left\lbrace {{\boldsymbol{\gamma }}}^{\top } \boldsymbol{Z}+g_2(\boldsymbol{X})+\phi \left(t\exp \lbrace {\boldsymbol{\beta }}^{\top }\boldsymbol{Z}+g_1(\boldsymbol{X})\rbrace \right)\right\rbrace$ and $f_{{\boldsymbol{\eta }}_0}(\boldsymbol{Z},\boldsymbol{X},t)=\exp \left\lbrace {{\boldsymbol{\gamma }}_0}^{\top } \boldsymbol{Z}+g_{20}(\boldsymbol{X})+\phi _0\left(t\exp \lbrace {\boldsymbol{\beta }}_0^{\top }\boldsymbol{Z}+g_{10}(\boldsymbol{X})\rbrace \right)\right\rbrace .$ Define


\begin{eqnarray*}
m_{{\boldsymbol{\eta }}}(\boldsymbol{O})= m_{({\boldsymbol{\beta }},{\boldsymbol{\gamma }},g_1,g_2,\phi )}(\boldsymbol{O}) & =&\Delta _1\log [1-\exp \lbrace -f_{{\boldsymbol{\eta }}}(\boldsymbol{Z},\boldsymbol{X},U)\rbrace ]\\&& +\Delta _2\log [\exp \lbrace -f_{{\boldsymbol{\eta }}}(\boldsymbol{Z},\boldsymbol{X},U)\rbrace \\&&\qquad \qquad -\exp \lbrace -f_{{\boldsymbol{\eta }}}(\boldsymbol{Z},\boldsymbol{X},V)\rbrace ] \\&& -(1-\Delta _1-\Delta _2)f_{{\boldsymbol{\eta }}}(\boldsymbol{Z},\boldsymbol{X},V),
\end{eqnarray*}

and $h_{{\boldsymbol{\eta }}}(\boldsymbol{O})=m_{{\boldsymbol{\eta }}_0}(\boldsymbol{O})-m_{{\boldsymbol{\eta }}}(\boldsymbol{O})$. It follows from the above conditions that $\exp \lbrace -pA-\mathcal {B}-\mathcal {C}\rbrace \le f_{{\boldsymbol{\eta }}}(\boldsymbol{Z},\boldsymbol{X},t)\le \exp \lbrace pA+\mathcal {B}+\mathcal {C}\rbrace \mbox{and} \Lambda _0(M_1)\exp \lbrace -pA-B_0\rbrace \le f_{{\boldsymbol{\eta }}_0}(\boldsymbol{Z},\boldsymbol{X},t)\le \Lambda _0(M_2)\exp \lbrace pA+B_0\rbrace a.s.$ for all $(\boldsymbol{Z}, \boldsymbol{X})\in [0,1]^{p+r}, t\in [M_1, M_2].$ Then, we have $|h_{{\boldsymbol{\eta }}}(\boldsymbol{O})|\le B a.s.,$ where


\begin{eqnarray*}
B &=& \exp \lbrace pA+\mathcal {B}+\mathcal {C}\rbrace +\Lambda _0(M_2)\exp (pA+B_0) -\\&& \log \left[1-\exp \left\lbrace -\exp (-pA-\mathcal {B}-\mathcal {C})\right\rbrace \right]\\&&-\log \left[1-\exp \left\lbrace -\Lambda _0(M_1)\exp (-pA-B_0)\right\rbrace \right].
\end{eqnarray*}

Next, we present the nonasymptotic error bound.

Theorem 1: (Nonasymptotic error bound) Let $\mathrm{Pdim}(\mathcal {G}_n)$ denote the pseudo dimension of $\mathcal {G}_n$. Assume that the conditions $\rm (C1)$–$\rm (C6)$ hold, $1/(2\mu +1)< \nu < 1/(2\mu )$ with $\mu \ge 1$. Then, for any $N, M \in \mathbb {N}^+$, the function class $\mathcal {G}_n=\mathcal {G}_{\boldsymbol{p},\mathcal {L},\mathcal {B},\mathcal {W},\mathcal {S}}$ with depth $\mathcal {L}=21(\lfloor \xi \rfloor +1)^2M\lceil \log _2(8M)\rceil + 2r$ and width $\mathcal {W}=38(\lfloor \xi \rfloor +1)^23^{r}r^{\lfloor \xi \rfloor +1} N \lceil \log _2(8N)\rceil$, for $n> \mathrm{Pdim}(\mathcal {G}_n)$,

\begin{eqnarray*}
&&\mathbb {E}d^2(\widehat{{\boldsymbol{\eta }}}_n, {\boldsymbol{\eta }}_0)\\&&\le C\left\lbrace \frac{B^2C_0\lbrace p+q_n+\mathcal {S}\mathcal {L}\log (\mathcal {S})\rbrace \log n}{n} + n^{-2\mu \nu }\right.\\&& \left. +B_0^2(\lfloor \xi \rfloor +1)^4 r^{2\lfloor \xi \rfloor +\xi \vee 1}(NM)^{-4\xi /r}\right\rbrace ,
\end{eqnarray*}

where $C> 0$ is a constant not depending on $n,p,r,q_n,\mu ,\nu ,\xi ,B,B_0,C_0,\mathcal {S},\mathcal {L},N$, and *M*.

Remark 1: By appropriately selecting $\nu$, *M*, and *N* to balance the stochastic error and the approximation error, one can attain the optimal convergence rate. Choosing $\nu =\frac{\xi }{\mu (2\xi +r)}$ and $N \asymp \left(\frac{n}{\log n}\right)^{\frac{r}{2r+4\xi }}$, and taking *M* to be a fixed positive constant independent of *n*, we obtain

(7) \begin{eqnarray*}
\mathbb {E}d(\widehat{{\boldsymbol{\eta }}}_n, {\boldsymbol{\eta }}_0)\lesssim \left(\frac{\log n}{n}\right)^{\frac{\xi }{2\xi +r}}.
\end{eqnarray*}

For a fixed dimension *p*, (7) implies that as the sample size $n \rightarrow \infty$, the estimate $\widehat{{\boldsymbol{\eta }}}_n$ converges to the true function ${\boldsymbol{\eta }}_0$ at the rate $\left(\frac{\log n}{n}\right)^{\xi /(2\xi +r)}.$

Denote ${\boldsymbol{\theta }}=({\boldsymbol{\beta }}^\top ,{\boldsymbol{\gamma }}^\top )^\top$ and ${\boldsymbol{\theta }}_0=({\boldsymbol{\beta }}_0^\top ,{\boldsymbol{\gamma }}_0^\top )^\top$. Next, we establish the efficient score and information bound for estimating ${\boldsymbol{\theta }}_0.$ Let $\Omega _0=\lbrace \phi \in L^2([0,\tau ]):-\infty < \phi (\tau )< \infty \rbrace$ and $\mathcal {H}_0=\lbrace g\in \mathcal {H}^\xi ([0,1]^r, B_0):\mathbb {E} g(\boldsymbol{X}) =0\rbrace .$ Let $\mathcal {H}_{g_0}$ denote the collection of all subfamilies $\lbrace g_s\in L^2([0,1]^r): s\in (-1,1)\rbrace \subset \mathcal {H}_0$ such that $\lim _{s\rightarrow 0}\Vert s^{-1}(g_s-g_0)-a\Vert _{L^2([0,1]^r)}=0,$ where $a\in L^2([0,1]^r)$, and let


\begin{eqnarray*}
&& \mathbb {S}_{g_0}=\Big \lbrace a\in L^2([0,1]^r):\lim _{s\rightarrow 0}\Vert s^{-1}(g_s-g_0)-a\Vert _{L^2([0,1]^r)}=0\\&& \mbox{for some subfamily} \\&& \lbrace g_s:s\in (-1,1)\rbrace \in \mathcal {H}_{g_0},\,\,\mbox{and}\,\, \mathbb {E}a(\boldsymbol{X})=0\Big \rbrace.
\end{eqnarray*}

Likewise, let $\Omega _{\phi _0}$ be the collection of all subfamilies $\lbrace \phi _q:q\in (-1,1)\rbrace \subset \Omega _0$ such that $\lim _{q\rightarrow 0}\Vert q^{-1}(\phi _q-\phi _0)-c\Vert _{L^2([0,\tau ])}=0$, where $h\in L^2([0,\tau ]),$ and let


\begin{eqnarray*}
\begin{split} \mathbb {S}_{\phi _0}=\Big \lbrace h\in L^2([0,\tau ]):\lim _{q\rightarrow 0}\Vert q^{-1}(\phi _q-\phi _0)-h\Vert _{L^2([0,\tau ])}=0 \,\, \mbox{for } \\\mbox{some subfamily} \,\,\lbrace \phi _q:q\in (-1,1)\rbrace \in \Omega _{\phi _0}\Big \rbrace . \end{split}
\end{eqnarray*}

Let ${\overline {\mathbb{S}}}_{g_0}$ and ${\overline {\mathbb{S}}}_{\phi _0}$ be the closed linear span of ${\mathbb {S}}_{g_0}$ and ${\mathbb {S}}_{\Lambda _0}$, respectively. $\dot{\ell }_{{\boldsymbol{\theta }}}$ denotes the first-order partial derivative of $m_{{\boldsymbol{\eta }}}$ with respect to parameter ${\boldsymbol{\theta }}$. $\dot{\ell }_{g_1},\dot{\ell }_{g_2}$, and $\dot{\ell }_{\phi }$ represent the directional derivatives of $m_{{\boldsymbol{\eta }}}$ with respect to functions $g_1, g_2$, and $\phi$, respectively. Their detailed definitions are provided in the supplementary materials.

Theorem 2: (Efficient score and information bound) Assume that the conditions $\rm (C1)$–$\rm (C7)$ hold. Then, the efficient score for ${\boldsymbol{\theta }}_0=({\boldsymbol{\beta }}_0^{\top }, {\boldsymbol{\gamma }}_0^{\top })^{\top }$ is

\begin{eqnarray*}
\ell ^{*}_{{\boldsymbol{\theta }}}({\boldsymbol{\eta }}_0) =\dot{\ell }_{{\boldsymbol{\theta }}}({\boldsymbol{\eta }}_0)-\dot{\ell }_{g_1}({\boldsymbol{\eta }}_0)[\boldsymbol{a}^{*}]-\dot{\ell }_{g_2}({\boldsymbol{\eta }}_0)[\boldsymbol{b}^{*}]-\dot{\ell }_{\phi }({\boldsymbol{\eta }}_0)[\boldsymbol{h}^{*}].
\end{eqnarray*}

Here, $(\boldsymbol{a}^{*\top },\boldsymbol{b}^{*\top }, \boldsymbol{h}^{*\top })^\top \in {\overline {\mathbb{S}}}_{g_0}^{2p} \times {\overline {\mathbb{S}}}_{g_0}^{2p} \times {\overline {\mathbb{S}}}_{\phi _0}^{2p}$ is the least favorable direction that minimizes the distance $\mathbb {E}\Vert \dot{\ell }_{{\boldsymbol{\theta }}}({\boldsymbol{\eta }}_0)-\dot{\ell }_{g_1}({\boldsymbol{\eta }}_0)[\boldsymbol{a}]-\dot{\ell }_{g_2}({\boldsymbol{\eta }}_0)[\boldsymbol{b}]-\dot{\ell }_{\phi }({\boldsymbol{\eta }}_0)[\boldsymbol{h}] \Vert _c^2$, where the notation $\Vert \boldsymbol{v}\Vert _c^2=(v_1^2,\ldots ,v_p^2)^\top$ for a vector $\boldsymbol{v}=(v_1,\ldots ,v_p)^\top$ and the minimization operates component-wise on the vector. Moreover, the information bound of ${\boldsymbol{\theta }}_0$ is

\begin{eqnarray*}
&& {\boldsymbol{I}}({\boldsymbol{\theta }}_0)=\mathbb {E}\lbrace \ell ^{*}_{{\boldsymbol{\theta }}}({\boldsymbol{\eta }}_0)^{\otimes 2}\rbrace =\mathbb {E}\left\lbrace \dot{\ell }_{{\boldsymbol{\theta }}}({\boldsymbol{\eta }}_0)-\dot{\ell }_{g_1}({\boldsymbol{\eta }}_0)[\boldsymbol{a}^{*}]-\dot{\ell }_{g_2}({\boldsymbol{\eta }}_0)[\boldsymbol{b}^{*}]\right.\\&& \left.-\dot{\ell }_{\phi }({\boldsymbol{\eta }}_0)[\boldsymbol{h}^{*}]\right\rbrace ^{\otimes 2}.
\end{eqnarray*}

The next theorem gives the asymptotic normality of $\widehat{{\boldsymbol{\theta }}}_n=(\widehat{{\boldsymbol{\beta }}}_n^\top ,\widehat{{\boldsymbol{\gamma }}}_n^\top )^\top$ with $\sqrt{n}$-consistency.

Theorem 3: (Asymptotic normality) Assume that the conditions $\rm (C1)$–$\rm (C7)$ hold and the information matrix ${\boldsymbol{I}}\left({\boldsymbol{\theta }}_0\right)$ is nonsingular. Suppose $N \asymp \left(\frac{n}{\log n}\right)^{\frac{p+1}{2(p+1)+4\xi }},$  *M* and *p* be fixed constants independent of *n*, and $\nu =\xi /(\mu (2\xi +r))$ with $\mu \ge 1$ and $\xi > r/2$, then $\sqrt{n}\left(\widehat{{\boldsymbol{\theta }}}_n-{\boldsymbol{\theta }}_0\right) \stackrel{d}{\longrightarrow } \mathcal {N}\left(0, {\boldsymbol{I}}^{-1}\left({\boldsymbol{\theta }}_{0}\right)\right), \,\, \mbox{as} \,\, n \rightarrow \infty .$

Under the assumptions in Theorem 3, the estimate $\widehat{{\boldsymbol{\eta }}}_n$ converges to ${\boldsymbol{\eta }}_0$ at a rate faster than $n^{-1/4}$, which is required for the validity of the theorem. The proofs of Theorems 1–3 are shown in Web Appendix A.

Remark 2: Unlike partial likelihood approaches that bypass the baseline hazard, establishing our nonasymptotic bounds required bounding the product space of DNNs and monotonic splines $\mathcal {G}_n \times \mathcal {M}_n$. Furthermore, to prove asymptotic normality and semiparametric efficiency, we explicitly computed the score operators and projected the finite-dimensional parameter score onto the joint tangent space of all infinite-dimensional nuisance parameters.

### 3.2 Nonparametric extension

When the objective is prediction rather than interpretability, one can pool all covariates into a single feature vector $\boldsymbol{W} = (\boldsymbol{Z}^\top , \boldsymbol{X}^\top )^\top \in \mathbb {R}^{p+r}$. Thus, model (1) degenerates to


(8) \begin{eqnarray*}
\Lambda (t| \boldsymbol{W})=\Lambda \left(t \exp \left\lbrace g_1(\boldsymbol{W})\right\rbrace \right)\exp \left\lbrace g_2(\boldsymbol{W})\right\rbrace .
\end{eqnarray*}

At this point, the log-likelihood function degenerates into


\begin{eqnarray*}
\begin{aligned} &\ell _n\left(g_1, g_2, \phi \right) = \sum _{i=1}^n\left[\Delta _{1i} \log \left\lbrace 1-\exp \left[- \exp \left\lbrace g_2\left(\boldsymbol{W}_i\right)+\phi \left(U_i \exp \left\lbrace g_1\left(\boldsymbol{W}_i\right)\right\rbrace \right)\right\rbrace \right]\right\rbrace \right.\\&\qquad \qquad \qquad \qquad +\Delta _{2 i} \log \left\lbrace \exp \left[- \exp \left\lbrace g_2\left(\boldsymbol{W}_i\right)+\phi \left(U_i \exp \left\lbrace g_1\left(\boldsymbol{W}_i\right)\right\rbrace \right)\right\rbrace \right]\right.\\&\qquad \qquad \qquad \qquad \qquad \qquad \left.- \exp \left[- \exp \left\lbrace g_2\left(\boldsymbol{W}_i\right)+\phi \left(V_i \exp \left\lbrace g_1\left(\boldsymbol{W}_i\right)\right\rbrace \right)\right\rbrace \right]\right\rbrace \\&\left.\qquad \qquad \qquad \qquad -(1-\Delta _{1i}-\Delta _{2i})\exp \left\lbrace g_2\left(\boldsymbol{W}_i\right)+\phi \left(V_i \exp \left\lbrace g_1\left(\boldsymbol{W}_i\right)\right\rbrace \right)\right\rbrace \right]. \end{aligned}
\end{eqnarray*}

Similarly, we use DNNs to estimate $g_1(\cdot )$ and $g_2(\cdot )$, and monotone splines to estimate $\phi (\cdot )$. This method is called nonparametric deep generalized accelerated hazards model (DGAHM-Non). Denote $\zeta = (g_1, g_2, \phi )$, and let $\zeta _0 = (g_{10}, g_{20}, \phi _0)$ be the true value of $\zeta$. Meanwhile, we introduce the corresponding metric:


\begin{eqnarray*}
&& d\left(\zeta _1,\zeta _2\right) = \left\lbrace \left\Vert g_{11}-g_{12}\right\Vert _{L^2\left([0,1]^{p+r}\right)}^2 +\left\Vert g_{21}-g_{22}\right\Vert _{L^2\left([0,1]^{p+r}\right)}^2\right.\\&& \left.+\left\Vert \phi _1-\phi _2\right\Vert _{\Phi }^2\right\rbrace ^{1 / 2}.
\end{eqnarray*}

We give the corresponding regularity assumptions: (C1$^{\prime }$) The functions $g_{10}$ and $g_{20}$ belong to the Hölder class $\mathcal {H}^\xi ([0,1]^{p+r}, B_0)$ defined in (6). (C2$^{\prime }$) The covariates $\boldsymbol{W}$ take values in a bounded subset of $\mathbb {R}^{p+r}$. Without loss of generality, we assume $\boldsymbol{W}\in [0,1]^{p+r}.$ (C3$^{\prime }$) Same as (C3). (C4$^{\prime }$) Same as (C4). (C5$^{\prime }$) The nonparametric function $g_{10}$ and $g_{20}$ satisfy $\mathbb {E}g_{10}(\boldsymbol{W})=0$ and $\mathbb {E}g_{20}(\boldsymbol{W})=0$.

Being a special case of the semiparametric framework, this allows us to readily derive the nonparametric error bound for the estimator $\hat{\zeta }_n$ via Theorem 1.

Corollary 1: Let $\mathrm{Pdim}(\mathcal {G}_n)$ denote the pseudo dimension of $\mathcal {G}_n$. Assume that the conditions $\rm (C1^{\prime })$–$\rm (C5^{\prime })$ hold, $1/(2\mu +1)< \nu < 1/(2\mu )$ with $\mu \ge 1$. Then, for any $N, M \in \mathbb {N}^+$, the function class $\mathcal {G}_n=\mathcal {G}_{\boldsymbol{p},\mathcal {L},\mathcal {B},\mathcal {W},\mathcal {S}}$ with depth $\mathcal {L}=21(\lfloor \xi \rfloor +1)^2M\lceil \log _2(8M)\rceil +2(p+r)$ and width $\mathcal {W}=38(\lfloor \xi \rfloor +1)^2(p+r)^{\lfloor \xi \rfloor +1} N \lceil \log _2(8N)\rceil$, for $n> \mathrm{Pdim}(\mathcal {G}_n)$,

\begin{eqnarray*}
&&\mathbb {E}d^2(\widehat{\zeta }_n, \zeta _0)\\&&\le C\left\lbrace \frac{B^2C_0\lbrace q_n+\mathcal {S}\mathcal {L}\log (\mathcal {S})\rbrace \log n}{n} + n^{-2\mu \nu }\right.\\&& \left. +B_0^2(\lfloor \xi \rfloor +1)^4(p+r)^{2\lfloor \xi \rfloor +\xi \vee 1}(NM)^{-4\xi /(p+r)}\right\rbrace ,
\end{eqnarray*}

where $C> 0$ is a constant not depending on $n,p,r,q_n,\mu ,\nu ,\xi ,B,B_0,C_0,\mathcal {S},\mathcal {L},N$, and *M*.

## 4 Computation

### 4.1 Implementation details

In the following, we provide a detailed description of the computation. First, we describe the libraries and functions utilized to implement our proposed DNN-based methodology. For DNN estimation, we use the Pytorch library (Paszke et al., 2019) to minimize the negative log-likelihood function $L({\boldsymbol{\beta }}, {\boldsymbol{\gamma }}, g_1,g_2,\boldsymbol{c}^{\top }\boldsymbol{B}_m(t)) = -\ell _n({\boldsymbol{\beta }}, {\boldsymbol{\gamma }}, g_1,g_2,\boldsymbol{c}^{\top }\boldsymbol{B}_m(t))$ and estimate the neural network parameters with the widely adopted Adam optimizer (Kingma and Ba, 2014). For the estimation of ${\boldsymbol{\beta }}$, ${\boldsymbol{\gamma }}$, and $\boldsymbol{c}$, we can use the minimize function from the optimize module in the scipy library. This function supports the optimization of parameters with constraints, making it suitable for optimizing these parameters.

Interval-censored data necessitates maximizing the full log-likelihood, which highly couples the baseline cumulative hazard with nonlinear covariate effects and destabilizes traditional simultaneous gradient updates. To address this, we introduce an alternating optimization-based sieve maximum likelihood method to decouple and solve for the parameters, with detailed steps provided in Algorithm 1 in Web Appendix C.

The neural network parameters are initialized using default random initialization. The hyperparameters include the number of hidden layers $\mathcal {L}$, the number of neurons $\mathcal {W}$ in each hidden layer, the dropout rate (Srivastava et al., 2014), and the learning rate (Goodfellow et al., 2016) in deep learning. These hyperparameters need to be specified for implementing $g_{10}$ and $g_{20}$. In our approach, we consider the same number of neurons in each hidden layer. Candidate hyperparameters for DNNs in simulations and data applications are shown in Table S1 in Web Appendix B. After each training trial, we can obtain the loss function over a validation set. By comparing the sizes of the loss functions in the validation set, the candidates corresponding to the smallest loss function are the best hyperparameters.

### 4.2 Estimation of the information bound

Performing inference on the parameter $\theta _0$ requires estimating the asymptotic covariance matrix $\boldsymbol{I}^{-1}(\theta _0)$ as described in Theorem 3. Recall that the information bound


\begin{eqnarray*}
{\boldsymbol{I}}({\boldsymbol{\theta }}_0)=\mathbb {E}\left\lbrace \dot{\ell }_{{\boldsymbol{\theta }}}({\boldsymbol{\eta }}_0)-\dot{\ell }_{g_1}({\boldsymbol{\eta }}_0)[\boldsymbol{a}^{*}]-\dot{\ell }_{g_2}({\boldsymbol{\eta }}_0)[\boldsymbol{b}^{*}]-\dot{\ell }_{\phi }({\boldsymbol{\eta }}_0)[\boldsymbol{h}^{*}]\right\rbrace ^{\otimes 2},
\end{eqnarray*}

where $\dot{\ell }_{{\boldsymbol{\theta }}}({\boldsymbol{\eta }}_0)$, $\dot{\ell }_{g_1}({\boldsymbol{\eta }}_0)[\boldsymbol{a}^{*}]$, $\dot{\ell }_{g_2}({\boldsymbol{\eta }}_0)[\boldsymbol{b}^{*}]$ and $\dot{\ell }_{\phi }({\boldsymbol{\eta }}_0)[\boldsymbol{h}^{*}]$ are defined in the supplementary materials and $(\boldsymbol{a}^{*},\boldsymbol{b}^{*},\boldsymbol{h}^{*})$ is the minimizer defined in Theorem 2. In practice, the estimate of $(\mathbf {\boldsymbol{a}}^{*}, \mathbf {\boldsymbol{b}}^{*}, \mathbf {\boldsymbol{h}}^{*})$ is obtained by minimizing an empirical objective function, as shown below


\begin{eqnarray*}
(\widehat{\boldsymbol{a}}^{*},\widehat{\boldsymbol{b}}^{*},\widehat{\boldsymbol{h}}^{*})=\mathop {\arg \min }_{(\boldsymbol{a},\boldsymbol{b},\boldsymbol{h})} \mathbb {E}_n\Vert \dot{\ell }_{{\boldsymbol{\theta }}}(\widehat{{\boldsymbol{\eta }}})-\dot{\ell }_{g_1}(\widehat{{\boldsymbol{\eta }}})[\boldsymbol{a}]-\dot{\ell }_{g_2}(\widehat{{\boldsymbol{\eta }}})[\boldsymbol{b}]-\dot{\ell }_{\phi }(\widehat{{\boldsymbol{\eta }}})[\boldsymbol{h}]\Vert _c^2,
\end{eqnarray*}

where $\mathbb {E}_n$ denotes the sample average. However, the high dimensionality of the functions $\boldsymbol{a},\boldsymbol{b}$, and $\boldsymbol{h}$ makes it difficult to obtain convincing solutions using classical nonparametric methods such as kernel regression and spline regression. As a result, we utilize a DNN to approximate $(\boldsymbol{a}^{*},\boldsymbol{b}^{*},\boldsymbol{h}^{*}).$ In this DNN, the inputs and outputs are $(U,V,\boldsymbol{Z},\boldsymbol{X})$ and


\begin{eqnarray*}
\left(\boldsymbol{a}(\boldsymbol{X}),\boldsymbol{b}(\boldsymbol{X}),\boldsymbol{h}\left(U\exp \left\lbrace \widehat{{\boldsymbol{\beta }}}^\top \boldsymbol{Z}+\widehat{g}_{1}(\boldsymbol{X})\right\rbrace \right),\boldsymbol{h}\left(V\exp \left\lbrace \widehat{{\boldsymbol{\beta }}}^\top \boldsymbol{Z}+\widehat{g}_{1}(\boldsymbol{X})\right\rbrace \right)\right),
\end{eqnarray*}

respectively. The details of the implementation of this DNN resemble the network used to maximize the log-likelihood function in Equation (5). Armed with the resulting estimates


\begin{eqnarray*}
&& \left(\widehat{\boldsymbol{a}}^{*}(\boldsymbol{X}_i),\widehat{\boldsymbol{b}}^{*}(\boldsymbol{X}_i),\widehat{\boldsymbol{h}}^{*}\left(U_i\exp \left\lbrace \widehat{{\boldsymbol{\beta }}}^\top \boldsymbol{Z_i}+\widehat{g}_{1}(\boldsymbol{X_i})\right\rbrace \right),\widehat{\boldsymbol{h}}^{*}\right.\\&& \left.\left(V_i\exp \left\lbrace \widehat{{\boldsymbol{\beta }}}^\top \boldsymbol{Z_i}+\widehat{g}_{1}(\boldsymbol{X_i})\right\rbrace \right)\right),
\end{eqnarray*}

the information bound can be estimated by


\begin{eqnarray*}
{\widehat{\boldsymbol{I}}}({\boldsymbol{\theta }}_0)=\mathbb {E}_n\left\lbrace \dot{\ell }_{{\boldsymbol{\theta }}}(\widehat{{\boldsymbol{\eta }}})-\dot{\ell }_{g_1}(\widehat{{\boldsymbol{\eta }}})[\widehat{\boldsymbol{a}}^{*}]-\dot{\ell }_{g_2}(\widehat{{\boldsymbol{\eta }}})[\widehat{\boldsymbol{b}}^{*}]-\dot{\ell }_{\phi }(\widehat{{\boldsymbol{\eta }}})[\widehat{\boldsymbol{h}}^{*}]\right\rbrace ^{\otimes 2}.
\end{eqnarray*}

## 5 Simulations

### 5.1 Semiparametric results

Simulation studies are conducted to evaluate the finite-sample performance of the proposed DGAHM method. Furthermore, the numerical results of the extended hazard model (EHM) (Chen and Jewell, 2001) are displayed for comparison. In EHM, the cumulative hazard function, given the covariates $(\boldsymbol{Z}, \boldsymbol{X})$, is assumed to take the following form:


\begin{eqnarray*}
\Lambda (t| \boldsymbol{Z}, \boldsymbol{X})=\Lambda \left(t \exp ({\boldsymbol{\beta }}^{\top } \boldsymbol{Z}+{\boldsymbol{\alpha }}^{\top }\boldsymbol{X}+\nu _1)\right)\exp \left({\boldsymbol{\gamma }}^{\top } \boldsymbol{Z}+{\boldsymbol{\xi }}^{\top } \boldsymbol{X}+\nu _2\right),
\end{eqnarray*}

where $\nu _1$ and $\nu _2$ are 2 intercept parameters.

To thoroughly explore alternative formulations, we also implement an intermediate variant, termed DGAHM-Mid. Specifically, the characteristic of interval-censored data is that the event time cannot be accurately observed, and we can only determine whether it occurs within a specified interval. Lindsey (1998) proposed a simpler midpoint imputation, replacing each true survival time $T_i$ by the midpoint of its censoring interval. Incorporating this into our proposed model, we approximate the true survival time $T_i$ by the midpoint of the interval whenever an event occurs, treating it as a precise observation. Conditional on covariates $\boldsymbol{W}_i = (\boldsymbol{Z}_i^\top , \boldsymbol{X}_i^\top )^\top$, the log-likelihood function derived from this DGAHM-Mid approach is


\begin{eqnarray*}
&& \sum _{i=1}^n\left\lbrace \Delta _{1i}\log f\left(\frac{U_i}{2}\Big |\boldsymbol{W}_i\right) + \Delta _{2i}\log f\left(\frac{U_i+V_i}{2}\Big |\boldsymbol{W}_i\right)\right.\\&& \left.+(1-\Delta _{1i}-\Delta _{2i})\log S(V_i|\boldsymbol{W}_i)\right\rbrace ,
\end{eqnarray*}

where $f(t| \boldsymbol{W}_i) = \lambda \big (t \exp \lbrace {\boldsymbol{\beta }}^{\top }\boldsymbol{Z}_i + g_1(\boldsymbol{X}_i)\rbrace \big )\, \exp \lbrace {\boldsymbol{\beta }}^{\top }\boldsymbol{Z}_i + g_1(\boldsymbol{X}_i)+{\boldsymbol{\gamma }}^{\top }\boldsymbol{Z}_i+g_2(\boldsymbol{X}_i)\rbrace \, \exp \big \lbrace -\Lambda (t| \boldsymbol{Z}_i, \boldsymbol{X}_i)\big \rbrace$, and $\lambda (t) = d\Lambda (t)/dt$.

The simulation settings are designed as follows. For all simulations, *Z* was generated from a Bernoulli distribution with a success probability of 0.5, and $\boldsymbol{X}=(X_1,X_2,X_3,X_4)^{\top }$ was generated from a multivariate normal distribution (with a correlation of 0.5) truncated to the interval [0,1]. We set the true parameters as $(\beta _0, \gamma _0)=(2, 1)$. We considered 4 different scenarios for $g_{10}(\boldsymbol{x})$ and $g_{20}(\boldsymbol{x})$, where $\boldsymbol{x}=(x_1,x_2,x_3,x_4)^{\top }\in [0,1]^4$ for each case:

- Case 1 (Linear): $g_{10}(\boldsymbol{x})=x_1/4+x_2/3+x_3/2+x_4/5-0.4, g_{20}(\boldsymbol{x})=x_1/5+x_2/2+x_3/3+x_4/2-0.48;$
- Case 2 (Additive): $g_{10}(\boldsymbol{x})=\log (x_1+1)/3+\exp (x_2)/5+x_3^2/4+x_4/2-0.6, g_{20}(\boldsymbol{x})=x_1+\sqrt{x_2}/2+\log (x_3+1)/3+x_4^2/4-0.65;$
- Case 3 (Deep 1): $g_{10}(\boldsymbol{x})=\sqrt{x_1x_2}/2+\log (x_2x_3+1)/3+\exp (x_4)/4-0.52, g_{20}(\boldsymbol{x})=x_1x_2/2+x_3^2x_4/3+\log (x_4+1)-0.36;$
- Case 4 (Deep 2): $g_{10}(\boldsymbol{x})=\left\lbrace \sqrt{x_1x_2}/2+\log (x_2x_3+1)/3+\exp (x_4)/4\right\rbrace ^2/2-0.19, g_{20}(\boldsymbol{x})=\left\lbrace x_1x_2/2+x_3^2x_4/3+\log (x_4+1)\right\rbrace ^2-0.16.$

The various intercept terms were added to $g_{10}$ and $g_{20}$ to guarantee all 4 cases satisfy $\mathbb {E}g_{10}(\boldsymbol{X})=0$ and $\mathbb {E}g_{20}(\boldsymbol{X})=0.$ To get interval-censored observations, we randomly generated 2 observation times under 2 different baseline cumulative hazard functions in each case:

-
$\Lambda _0(t)=t/8$ ,

1. Cases 1–3: $U\sim {\rm Uniform}(0, \tau /5)$ and $V\sim \min \lbrace \tau /3+U+\tau \cdot {\rm Exponential}(1)/5, \tau \rbrace$;
2. Case 4: $U\sim {\rm Uniform}(0, 2\tau /11)$ and $V\sim \min \lbrace \tau /3+U+\tau \cdot {\rm Exponential}(1)/4, \tau \rbrace$;

-
$\Lambda _0(t)=t^{3/4}/6$ ,

1. Case 1: $U\sim {\rm Uniform}(0, \tau /7)$ and $V\sim \min \lbrace \tau /2+U+\tau \cdot {\rm Exponential}(1)/2, \tau \rbrace$;
2. Case 2: $U\sim {\rm Uniform}(0, \tau /7)$ and $V\sim \min \lbrace \tau /3+U+\tau \cdot {\rm Exponential}(1)/2, \tau \rbrace$;
3. Case 3: $U\sim {\rm Uniform}(0, \tau /6)$ and $V\sim \min \lbrace 2\tau /5+U+\tau \cdot {\rm Exponential}(1)/3, \tau \rbrace$;
4. Case 4: $U\sim {\rm Uniform}(0, \tau /6)$ and $V\sim \min \lbrace 2\tau /5+U+\tau \cdot {\rm Exponential}(1)/4, \tau \rbrace$.

Given the covariates $(Z,\boldsymbol{X})$, the event time *T* was generated via the model $\Lambda (t|Z,\boldsymbol{X})=\Lambda _0(t\exp \lbrace \beta _0 Z+ g_{10}(\boldsymbol{X})\rbrace )\exp \lbrace \gamma _0 Z+ g_{20}(\boldsymbol{X})\rbrace$. We set $\tau =5$, which yielded an average censoring rate of 30%–35% for both left- and right-censored observations across all simulation studies. In all simulation cases, we conducted 200 replications with sample sizes $n = 500$ or 1000. With $\Delta _{1i}=I_{[T_i\le U_i]}$, $\Delta _{2i}=I_{[U_i< T_i\le V_i]}$, and $\Delta _{3i}=1-\Delta _{1i}-\Delta _{2i}$, a sample of interval-censored data consists of $\lbrace (\Delta _{1i},\Delta _{2i},U_i,V_i,Z_i,\boldsymbol{X}_i): i=1,\ldots ,n\rbrace .$ In addition, we randomly generated 200 samples as a validation set to choose hyperparameters.

Table 1 shows the simulation results of the estimated $(\widehat{\beta }_n, \widehat{\gamma }_n)$ via the DGAHM, DGAHM-Mid, and EHM methods over 200 independent runs for each case. The results consist of the estimated biases (Bias), the sampling standard errors (SSE), the sampling means of the estimated standard error (ESE), and the 95% empirical coverage probabilities (CP). Results of the DGAHM method under each simulation setting demonstrate that the estimates of $(\beta _0, \gamma _0)$ seem to be asymptotically unbiased and the proposed variance estimation procedure provides reasonable estimates. The performance of the proposed estimator becomes better when the sample size increases from 500 to 1000 in all simulations. In contrast, the DGAHM-Mid method exhibits substantial biases across all scenarios, demonstrating its overall inadequacy. The DGAHM greatly outperforms the EHM method in the settings of Case 2, Case 3, and Case 4, where the overly restrictive EHM method causes large biases. Under the simpler Case 1 (which meets EHM assumptions), the DGAHM method remains strongly competitive with little loss of efficiency. In addition, the coverage rate is generally near 95% for our proposed DGAHM method. Under the last 3 cases, the poor coverage of EHM confidence intervals, along with the severe undercoverage of the DGAHM-Mid method across all cases, indicates that the uncertainty quantification produced by these alternative methods may be unreliable in practice.

**Table 1:** Simulation results of $\widehat{\beta }_n$ and $\widehat{\gamma }_n$ by the DGAHM, DGAHM-Mid, and EHM methods with 200 replications under 4 cases with $\Lambda _0(t)=t/8$ and $\Lambda _0(t)=t^{3/4}/6$.

				DGAHM				DGAHM-Mid				EHM			
$\Lambda _0(t)$		*n*		Bias	SSE	ESE	CP	Bias	SSE	ESE	CP	Bias	SSE	ESE	CP
$\frac{t}{8}$	Case 1	500	$\widehat{\beta }_n$	−0.007	0.141	0.152	0.935	−1.353	0.169	0.067	0.000	0.003	0.148	0.164	0.930
			$\widehat{\gamma }_n$	−0.031	0.097	0.111	0.930	0.820	0.131	0.155	0.010	−0.008	0.121	0.135	0.935
		1000	$\widehat{\beta }_n$	−0.006	0.096	0.122	0.945	−1.368	0.125	0.047	0.000	−0.004	0.093	0.114	0.960
			$\widehat{\gamma }_n$	−0.017	0.049	0.063	0.930	0.819	0.102	0.099	0.000	0.007	0.069	0.087	0.925
	Case 2	500	$\widehat{\beta }_n$	−0.020	0.133	0.157	0.940	−1.357	0.173	0.060	0.000	−0.488	0.893	0.949	0.975
			$\widehat{\gamma }_n$	−0.019	0.087	0.094	0.920	0.725	0.137	0.138	0.030	0.705	1.180	0.101	0.015
		1000	$\widehat{\beta }_n$	−0.017	0.099	0.114	0.930	−1.379	0.122	0.043	0.000	−0.531	0.619	0.638	0.960
			$\widehat{\gamma }_n$	−0.009	0.051	0.064	0.930	0.721	0.106	0.103	0.000	0.603	0.608	0.072	0.000
	Case 3	500	$\widehat{\beta }_n$	−0.029	0.152	0.160	0.930	−1.358	0.182	0.070	0.000	−0.535	0.626	0.414	0.750
			$\widehat{\gamma }_n$	−0.023	0.091	0.107	0.930	0.907	0.105	0.179	0.010	0.528	0.616	0.165	0.135
		1000	$\widehat{\beta }_n$	−0.010	0.093	0.118	0.955	−1.371	0.135	0.050	0.000	−0.480	0.813	0.265	0.425
			$\widehat{\gamma }_n$	−0.016	0.049	0.063	0.920	0.927	0.078	0.129	0.000	0.622	0.910	0.119	0.020
	Case 4	500	$\widehat{\beta }_n$	−0.007	0.141	0.158	0.940	−1.375	0.193	0.093	0.000	−0.409	0.988	0.383	0.710
			$\widehat{\gamma }_n$	−0.038	0.099	0.107	0.930	0.983	0.051	0.195	0.010	0.523	0.342	0.179	0.200
		1000	$\widehat{\beta }_n$	−0.005	0.091	0.105	0.945	−1.408	0.155	0.067	0.000	−0.435	0.599	0.249	0.295
			$\widehat{\gamma }_n$	−0.020	0.044	0.065	0.920	0.994	0.026	0.122	0.000	0.583	0.759	0.127	0.050
$\frac{t^{3/4}}{6}$	Case 1	500	$\widehat{\beta }_n$	−0.043	0.137	0.145	0.940	−1.309	0.228	0.090	0.000	−0.004	0.144	0.133	0.940
			$\widehat{\gamma }_n$	0.007	0.113	0.131	0.955	0.576	0.135	0.180	0.155	−0.004	0.111	0.132	0.925
		1000	$\widehat{\beta }_n$	−0.013	0.100	0.116	0.925	−1.322	0.151	0.061	0.000	0.007	0.114	0.127	0.935
			$\widehat{\gamma }_n$	0.005	0.072	0.083	0.940	0.525	0.113	0.132	0.075	0.002	0.075	0.084	0.935
	Case 2	500	$\widehat{\beta }_n$	0.012	0.140	0.150	0.925	−1.192	0.197	0.082	0.000	−0.758	0.446	0.471	0.590
			$\widehat{\gamma }_n$	−0.034	0.123	0.140	0.930	0.544	0.139	0.163	0.215	0.737	0.860	0.126	0.015
		1000	$\widehat{\beta }_n$	−0.009	0.098	0.111	0.935	−1.206	0.159	0.057	0.000	−0.681	0.942	0.348	0.300
			$\widehat{\gamma }_n$	0.000	0.075	0.084	0.950	0.519	0.135	0.107	0.045	0.785	0.979	0.086	0.005
	Case 3	500	$\widehat{\beta }_n$	−0.010	0.122	0.127	0.945	−1.226	0.191	0.083	0.000	−0.726	0.906	0.465	0.560
			$\widehat{\gamma }_n$	−0.015	0.119	0.139	0.940	0.638	0.131	0.176	0.095	0.745	0.899	0.127	0.015
		1000	$\widehat{\beta }_n$	−0.010	0.097	0.095	0.940	−1.259	0.157	0.054	0.000	−0.798	0.197	0.314	0.220
			$\widehat{\gamma }_n$	0.007	0.072	0.089	0.965	0.613	0.109	0.112	0.005	0.639	0.330	0.091	0.000
	Case 4	500	$\widehat{\beta }_n$	−0.038	0.158	0.158	0.925	−1.233	0.202	0.088	0.000	−0.820	0.252	0.462	0.530
			$\widehat{\gamma }_n$	0.005	0.120	0.132	0.935	0.746	0.171	0.190	0.075	0.631	0.614	0.132	0.030
		1000	$\widehat{\beta }_n$	−0.021	0.122	0.121	0.945	−1.258	0.151	0.059	0.000	−0.763	0.581	0.316	0.215
			$\widehat{\gamma }_n$	0.011	0.083	0.086	0.950	0.720	0.109	0.118	0.005	0.663	0.848	0.089	0.005

**Figure 1:**
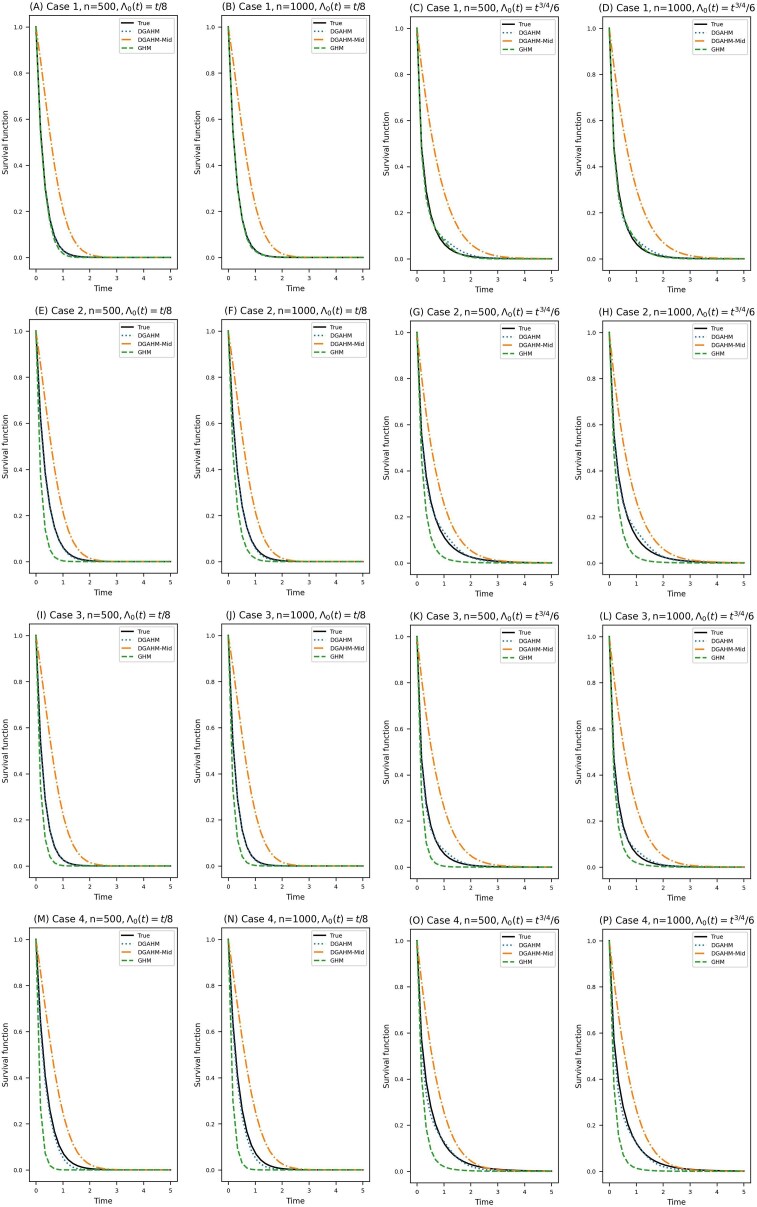
Predictions of survival functions by the DGAHM (dotted line), DGAHM-Mid (dash-dotted line), and EHM (dashed line) methods under 2 kinds of baseline cumulative hazard functions [$\Lambda _0(t)=t/8$ (the 2 leftmost columns) and $\Lambda _0(t)=t^{3/4}/6$ (the 2 rightmost columns)] for the subject with $Z=1$ and $\boldsymbol{X}=(0.1,0.3,0.5,0.7)^{\top }$, along with true values of the survival functions (solid black curve).

To evaluate the performance of our proposed method, we predict the survival functions of 4 subjects, respectively, with covariates: $Z=0$, $\boldsymbol{X}=(0.1,0.3,0.5,0.7)^{\top }$; $Z=1$, $\boldsymbol{X}=(0.1,0.3,0.5,0.7)^{\top }$; $Z=0$, $\boldsymbol{X}=(0.8,0.5,0.2,0)^{\top }$; and $Z=1$, $\boldsymbol{X}=(0.8,0.5,0.2,0)^{\top }$. Figure 1 depicts the prediction of survival functions by the DGAHM (dotted line), DGAHM-Mid (dash-dotted line), and EHM (dashed line) methods under 2 kinds of baseline cumulative hazard functions [$\Lambda _0(t)=t/8$ (the 2 leftmost columns) and $\Lambda _0(t)=t^{3/4}/6$ (the 2 rightmost columns)] for the subject with $Z=1$ and $\boldsymbol{X}=(0.1,0.3,0.5,0.7)^{\top }$, along with the true values of the survival functions (solid black curve). The predicted survival curves for the remaining 3 individuals are shown in Figures S1–S3 in Web Appendix B.

All figures demonstrate that the DGAHM estimates closely approximate the true survival function. In contrast, the DGAHM-Mid method exhibits substantial positive biases, consistently overestimating the survival probabilities across all cases and settings. Furthermore, the EHM estimates for Cases 2–4 also exhibit noticeable biases. These results suggest that the simple midpoint imputation in DGAHM-Mid is inadequate, whereas the proposed DGAHM method performs well and demonstrates robustness in all the situations considered.

### 5.2 Predictive performance

To evaluate the predictive performance of our proposed approaches under various complex settings, we conducted a simulation-based comparative study. Specifically, we compared the predictive accuracy of 4 different methods for handling interval-censored data: our proposed nonparametric approach (DGAHM-Non), the standard DGAHM, the naive midpoint imputation method (DGAHM-Mid) acting as a baseline for imputation strategies, and the existing neural network-based method (NN-IC) proposed by Sun and Ding (2023).

The covariates *Z* and $\boldsymbol{X}$ were generated in the same way as that in Section 5.1. We considered 5 different scenarios for the nonparametric multivariate functions $g_{10}(\boldsymbol{w})$ and $g_{20}(\boldsymbol{w})$, where $\boldsymbol{w}=(z, \boldsymbol{x}^{\top })^{\top }$ and $z \in \lbrace 0, 1\rbrace$:

- Scenario 1: $g_{10}(\boldsymbol{w})=z+x_1/2+x_2/3+x_3/4+x_4/5-0.9, g_{20}(\boldsymbol{w})=(z+x_1+x_2+x_3+x_4)/3-0.58;$
- Scenario 2: $g_{10}(\boldsymbol{w})= z/5+ \sin (\pi x_1/2)+\exp (x_2^2)/2+\log (x_3^3+1)+\cos (x_4) - 2.2, g_{20}(\boldsymbol{w})= z/10 + \exp (\sqrt{x_1}) / 2 + \cos \lbrace \sin (\pi x_2+1)\rbrace /2+\log (x_3^2 +1) + \sqrt{x_4} - 1.88;$
- Scenario 3: $g_{10}(\boldsymbol{w})=\log (2zx_1+2x_2+1)+\exp \lbrace x_3+\sin (\pi x_4/2)\rbrace /3-1.35, g_{20}(\boldsymbol{w})=z \sqrt{x_1+x_2}+ \exp \lbrace \cos (\pi x_3) + x_4\rbrace / 2 - 1.57;$
- Scenario 4: ${g_{10}(\boldsymbol{w})=\left[zx_1 +\cos (x_2)+\exp \lbrace x_3/3+\cos (\pi x_4)\rbrace /3\right]^2/2}$  $-1.6, g_{20}(\boldsymbol{w})=\sqrt{z\lbrace \sin (x_1)+x_2\rbrace + (x_3^2+1)\exp \lbrace \cos (\pi x_4)\rbrace / 2}$  $-1.14.$;
- Scenario 5: $g_{10}(\boldsymbol{w})=0, g_{20}(\boldsymbol{X})= \lbrace \sqrt{x_1^2+x_2^2} + \sin (x_3+x_4)\rbrace / 5 + \sum _{i=5}^{20} \beta _j x_j$, where $\beta _j=0$ for all $j=5,\ldots ,20.$

The various intercept terms were added to $g_{10}$ and $g_{20}$ to guarantee all 4 scenarios satisfy $\mathbb {E}g_{10}(\boldsymbol{W})=0$ and $\mathbb {E}g_{20}(\boldsymbol{W})=0.$ To get interval-censored observations, we randomly generated 2 observation times in each scenario:

- Scenario 1: $U\sim {\rm Uniform}(0, \tau /5)$ and $V\sim \min \lbrace \tau /4+U+\tau \cdot {\rm Exponential}(1)/3, \tau \rbrace$;
- Scenario 2: $U\sim {\rm Uniform}(0, \tau /6)$ and $V\sim \min \lbrace 2\tau /5+U+\tau \cdot {\rm Exponential}(1)/3, \tau \rbrace$;
- Scenario 3: $U\sim {\rm Uniform}(0, \tau /5)$ and $V\sim \min \lbrace \tau /3+U+\tau \cdot {\rm Exponential}(1)/3, \tau \rbrace$;
- Scenario 4: $U\sim {\rm Uniform}(0, \tau /5)$ and $V\sim \min \lbrace \tau /5+U+\tau \cdot {\rm Exponential}(1)/5, \tau \rbrace$;
- Scenario 5: $U\sim {\rm Uniform}(0, \tau /6)$ and $V\sim \min \lbrace \tau /8+U+\tau \cdot {\rm Exponential}(1)/8, \tau \rbrace$.

We set the baseline cumulative hazard function $\Lambda _0(t)=\log \left(t+1\right)$. Given the covariates $\boldsymbol{W}$, the event time *T* was generated via the model $\Lambda (t|\boldsymbol{W})=\Lambda _0(t\exp \lbrace g_{10}(\boldsymbol{W})\rbrace )\exp \lbrace g_{20}(\boldsymbol{W})\rbrace$. We set $\tau =5$, which yielded an average censoring rate of 30%–35% for both left- and right-censored observations across all simulation studies. In each simulation scenario, the training set consisted of $n=500$ or 1000 observations, and an independent validation set of size 200 was generated to facilitate hyperparameter tuning.

For interval-censored data, Sun and Ding (2023) applied the mean squared prediction error (MSPE) as a performance metric. It quantifies the average integrated $L_2$ distance between the true survival function and its estimated counterpart, defined as follows: $L(\hat{S})=\frac{1}{n}\sum _{i=1}^n\frac{1}{T_i}\int _0^{T_i}\lbrace S_0(t|\boldsymbol{W}_i)-{\hat{S}}(t|\boldsymbol{W}_i)\rbrace ^2dt,$ where $S_0(t|\boldsymbol{W}_i) = \exp \lbrace - \Lambda _0(t|\boldsymbol{W}_i)\rbrace$ and ${\hat{S}}(t|\boldsymbol{W}_i) = \exp \lbrace -\hat{\Lambda }(t|\boldsymbol{W}_i)\rbrace$. Obviously, the smaller the value of $L(\hat{S})$ is, the more accurate the estimate is. Table 2 summarizes the MSPE averages and standard deviations for the DGAHM-Non, DGAHM, DGAHM-Mid, and NN-IC methods across all 5 scenarios, computed over 200 replications on a test set. As expected, when the sample size *n* increases from 500 to 1000, both the MSPE and SD values generally decrease across almost all scenarios and methods, indicating the consistency of the predictions.

**Table 2:** The MSPE averages and standard deviations (SD) for the DGAHM-Non, DGAHM, DGAHM-Mid, and NN-IC methods across 5 scenarios, based on 200 replications on the test data ($\times 100$).

		DGAHM-Non		DGAHM		DGAHM-Mid		NN-IC	
	*n*	MSPE	SD	MSPE	SD	MSPE	SD	MSPE	SD
Scenario 1	500	0.131	0.064	0.109	0.098	2.279	0.285	1.875	0.175
	1000	0.100	0.077	0.070	0.048	2.234	0.256	1.881	0.131
Scenario 2	500	0.236	0.065	0.219	0.096	3.561	0.516	2.718	0.323
	1000	0.195	0.050	0.175	0.057	3.503	0.453	2.751	0.234
Scenario 3	500	0.318	0.076	0.663	0.111	2.677	0.445	2.001	0.234
	1000	0.257	0.063	0.592	0.077	2.619	0.409	2.011	0.179
Scenario 4	500	0.371	0.180	0.741	0.157	2.072	0.155	1.213	0.084
	1000	0.277	0.134	0.682	0.139	2.045	0.159	1.215	0.061
Scenario 5	500	0.087	0.039	3.095	0.505	0.221	0.046	0.080	0.019
	1000	0.064	0.030	3.242	0.293	0.209	0.036	0.070	0.009

Comparing the different methods, DGAHM-Non demonstrates highly robust and superior predictive performance across the varying complexities of the 5 scenarios. In Scenarios 1 and 2, DGAHM achieves the lowest MSPE, closely followed by DGAHM-Non, and both significantly outperform DGAHM-Mid and NN-IC. In Scenarios 3 and 4, which involve more complex nonlinear structures, DGAHM-Non yields the best performance with the lowest MSPE, while DGAHM ranks second. In Scenario 5, which incorporates high-dimensional zero-coefficient covariates, NN-IC and DGAHM-Non exhibit the most competitive performances (with DGAHM-Non achieving the lowest MSPE at $n=1000$), whereas DGAHM shows a significant increase in prediction error. Furthermore, the consistently large MSPE values of DGAHM-Mid in Scenarios 1 through 4 further confirm that simple midpoint imputation is inadequate for interval-censored data in these complex settings. Overall, DGAHM-Non provides the most robust and accurate predictions across all considered situations.

## 6 Application to the Atherosclerosis Risk in Communities study

To further demonstrate the performance, our proposed methodology is now applied to the data from the Atherosclerosis Risk in Communities study, an epidemiological follow-up study consisting of participants aged 44–66, recruited from 4 communities in the United States: Forsyth County of North Carolina, Jackson of Mississippi, Minneapolis suburbs of Minnesota, and Washington County of Maryland. Participants underwent clinical examinations approximately every 3 years, with the schedule varying from person to person. The failure event of interest here is the onset of diabetes, which can only be observed between 2 consecutive examinations for each participant; therefore, the interval censoring time for each participant is defined by the time between their last 2 clinical examinations. Next, we will apply the proposed method to estimate the impact of various risk factors on the occurrence of diabetes.

In our analysis, we focus on a cohort of 12 204 participants, excluding individuals with prevalent diabetes, those with unknown baseline status, or missing values for risk factors and covariates. Among the included participants, 27% have interval-censored observations, while approximately 71% have right-censored observations. We randomly split the overall dataset into 2 parts: dataset 1 and dataset 2. The split is performed at an approximate ratio of 9:1, where dataset 1 contains approximately 90% of the overall data, and dataset 2 contains the remaining 10%. Table 3 summarizes the characteristics of the subjects in the overall dataset, dataset 1 and dataset 2. The dataset for each subject includes risk factors: body mass index, glucose level, high-density lipoprotein cholesterol level, total cholesterol level, age, systolic blood pressure, diastolic blood pressure, race, gender, and community.

**Table 3:** Summary of the subjects in the overall dataset, dataset 1 and dataset 2.

	Overall ($n=12\,204$)		Dataset 1 ($n=11\,000$)		Dataset 2 ($n=1204$)	
Censoring type	Number	Rate	Number	Rate	Number	Rate
Left-censored	210	1.7%	189	1.7%	21	1.8%
Interval-censored	3294	27.0%	2971	27.0%	323	26.8%
Right-censored	8700	71.3%	7840	71.3%	860	71.4%

In this study, we aim to investigate the magnitude of the treatment effect of the first 4 risk factors (body mass index, glucose level, high-density lipoprotein cholesterol level, and total cholesterol level) on the risk of diabetes. We designate *Z* to represent the first 4 risk factors and $\boldsymbol{X}$ to represent the remaining risk factors. Next, we consider using the model (1) to fit the data set. The candidates of hyperparameters for DNNs can be found in Table S1 in Web Appendix B. We consider using cubic monotone B-splines (ie, $m=3$) to estimate the baseline cumulative hazard function. The cardinality of the interior knot set $k_n$ is selected as ${\rm ceiling}(n^{1/3})$.

To obtain the size of the effect of the first 4 risk factors on the risk of diabetes, we use dataset 1 as the training set and dataset 2 as the validation set to select hyperparameters. For comparison, we also present the results obtained from the DGAHM-Mid and EHM methods. Table 4 lists the estimated effects along with the ESEs and *P*-values of the estimates for the first 4 risk factors by these 3 methods. Based on the DGAHM and EHM methods, the first 4 risk factors demonstrated a significant impact on diabetes risk. Specifically, a lower body mass index, reduced blood glucose levels, higher high-density lipoprotein cholesterol, and lower total cholesterol were significantly associated with a decreased risk of diabetes. In contrast, the DGAHM-Mid method indicated that the linear effect of total cholesterol on diabetes risk was not statistically significant. Furthermore, the EHM method revealed no significant linear association between diabetes risk and either age or diastolic blood pressure.

**Table 4:** Analysis results (Est: estimate of $\gamma _j$; ESE: estimated standard error; *P*-value: the *P*-value for testing $H_0$: $\gamma _j$ = 0) for the Atherosclerosis Risk in Communities study.

	DGAHM			DGAHM-Mid			EHM		
Covariates	Est	ESE	*P*-value	Est	ESE	*P*-value	Est	ESE	*P*-value
Body mass index	0.219	0.016	$< 0.001$	0.089	0.021	$< 0.001$	0.251	0.027	$< 0.001$
Glucose level	0.530	0.020	$< 0.001$	0.166	0.023	$< 0.001$	0.517	0.028	$< 0.001$
High-density lipoprotein	−0.295	0.024	$< 0.001$	−0.111	0.025	$< 0.001$	−0.284	0.036	$< 0.001$
Total cholesterol	0.062	0.020	0.002	0.032	0.021	0.128	0.071	0.032	0.027
Age	–	–	–	–	–	–	0.015	0.025	0.549
Systolic blood pressure	–	–	–	–	–	–	0.131	0.027	$< 0.001$
Diastolic blood pressure	–	–	–	–	–	–	−0.050	0.032	0.118

**Figure 2:**
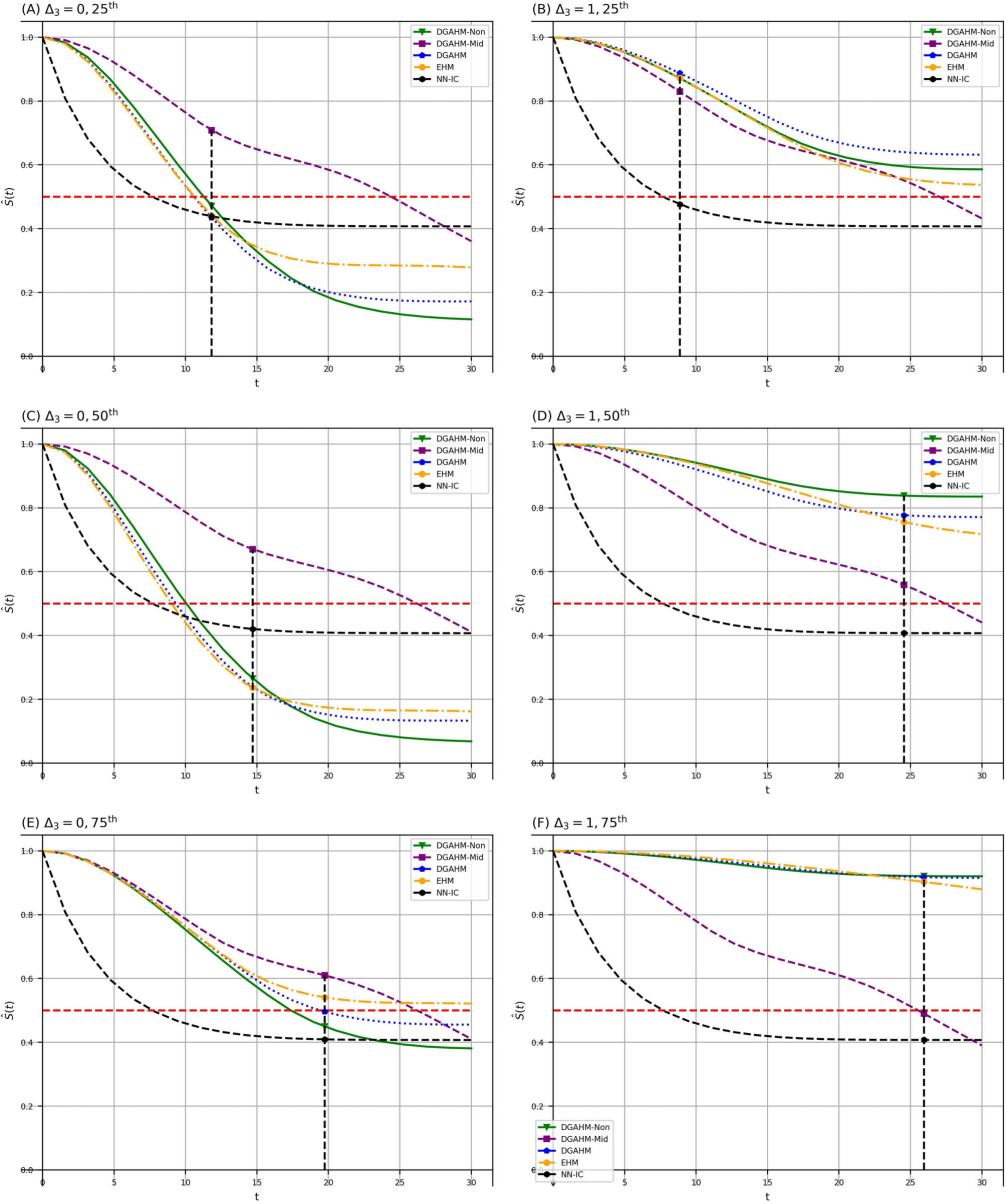
Predicted survival functions obtained by the DGAHM-Non (solid line), DGAHM-Mid (dashed line), DGAHM (dotted line), and EHM (dash-dotted line) methods. The solid horizontal line in all plots represents a survival probability of 0.5. The 3 plots from top to bottom on the left (or right) side represent the survival curves of individuals, where the event occurred (not occurred) in the population of the test set with observation times at the 25th, 50th, and 75th quantiles, respectively.

Furthermore, we evaluated the prediction performance [including survival probability prediction curve and integrated Brier score (IBS; Sun and Ding (2023))] of the DGAHM-Non, DGAHM-Mid, DGAHM, EHM, and NN-IC methods. To derive the survival curves, we employ a 5-fold split on dataset 1 for hyperparameter tuning, randomly allocating 4 folds for training and one for validation. For evaluation, we use dataset 2 as the test set. This test set comprises 2 groups: $\Delta _3=0$ (with 346 observations) and $\Delta _3=1$ (with 858 observations), indicating that the event of interest occurred and did not occur, respectively. In each group, we consider 3 subjects with the second observation time at the 25th, 50th, and 75th quantiles, respectively. Figure 2 shows the predicted survival functions for 6 representative subjects using the DGAHM-Non, DGAHM-Mid, DGAHM, EHM, and NN-IC methods. If an event occurs, its survival probability is lower than 0.5, and vice versa. In Figure 2, the solid horizontal line in all plots represents a survival probability of 0.5. The predicted survival function values obtained by the DGAHM-Non and DGAHM methods are all below (above) 0.5 in the case of $\Delta _3=0$ ($\Delta _3=1$) at all 3 observation times. In contrast, EHM predicts a value above 0.5 at the 75th subject when $\Delta _3=0$, and the DGAHM-Mid method also yields values above 0.5 at 3 observations for $\Delta _3=0$, which may be inconsistent with the actual status. We also calculated the IBS values of the 4 methods on the test set, which were 0.081 (DGAHM-Non), 0.108 (DGAHM-Mid), 0.083 (DGAHM), 0.083 (EHM), and 0.197 (NN-IC). Overall, DGAHM-Non demonstrates the highest predictive accuracy when capturing covariate effects on the hazard function, while the NN-IC method exhibits the worst performance. This implies that the Cox PH model is misspecified for this dataset, pointing to the likely existence of accelerated hazard effects.

## 7 Concluding remarks

In this paper, we present a DGAHM that preserves the interpretability of Cox models while incorporating complex nonparametric covariate effects via $\boldsymbol{X}$. This inclusive approach utilizes fully connected neural networks for nonparametric effects and monotonic splines for the baseline cumulative hazard, enhancing the prediction and analysis of diabetes risk factors. Under certain regularity conditions, we establish nonasymptotic error bounds and asymptotic normality for the parameter estimators, providing solid theoretical guarantees.

Building on this architecture, a key practical advantage of our model is the flexibility to balance interpretability and predictive power by tailoring covariate allocation. Variables requiring explicit clinical interpretation (eg, hazard ratios and acceleration factors) are assigned to the linear component $\boldsymbol{Z}$. Conversely, variables with suspected nonlinearities or complex interactions (including interactions between $\boldsymbol{X}$ and $\boldsymbol{Z}$) are assigned to $\boldsymbol{X}$, allowing the neural network to adjust for them nonparametrically.

Furthermore, our rigorous interval-censored framework addresses a critical clinical challenge: the uncertainty of true survival times, which depends directly on interval length. While naive midpoint imputation may yield acceptable estimates for narrow intervals (frequent visits), it introduces severe bias as intervals widen (infrequent visits). In such cases, our proposed exact interval-censoring approach becomes strictly necessary.

Despite its advantages in modeling flexibility and handling interval censoring, our method has limitations. Like most standard and deep survival models, it assumes conditionally independent censoring. Since real data often involve informative censoring, addressing dependent censoring via copulas, frailty models, or joint modeling within our DNN-spline architecture remains a valuable future direction.

Future work could address high-dimensional variable selection. In our framework, standard penalties (eg, Lasso, SCAD) can induce linear sparsity, while network-specific techniques such as first-layer sparse group Lasso or input gradient penalization identify nonlinear predictors. Nevertheless, theoretically guaranteeing simultaneous deep estimation and variable selection under interval censoring remains a challenging open problem.

## Supplementary Material

ujag140_Supplemental_Files Web Appendices, Tables, and Figures referenced in Sections 3–6 are available with this paper at the Biometrics website on Oxford Academic. The code for reproducing the results is available as a.zip file archive in the online Supplementary Materials.

## Data Availability

The data from the Atherosclerosis Risk in Communities study analyzed in Section 6 are available through the BioLINCC repository at https://biolincc.nhlbi.nih.gov/studies/aric.
