# Supplementary material for: Efficient estimation for deep generalized accelerated hazards models with interval-censored data
Source: Biometrics. 2026 Aug 10;82(3):ujag140. doi: 10.1093/biomtc/ujag140 (PMC13455595; doi:10.1093/biomtc/ujag140)
Supplement: Supplementary file 1 [file ujag140_supplemental_files.zip › SuppBIOM2025628M_final.pdf]

Supplementary Material for "Efficient Estimation for Deep Generalized Accelerated Hazards Models with Interval-censored Data" by Q. Wu, M. Du, S. Ma and X. Zhao

Web Appendix A: Proofs of Theorems 1-3

We take the negative log-likelihood function as the objective function, denoted as $L(\boldsymbol{\eta})$, that is, $L(\boldsymbol{\eta}) = -\mathbb{E}m_{\boldsymbol{\eta}}(\boldsymbol{O})$, and its empirical version is denoted as

$$L_n(\boldsymbol{\eta}) = -\frac{1}{n}\sum_{i=1}^{n}\{\Delta_{1i}\log[1-\exp\{-f_{\boldsymbol{\eta}}(\boldsymbol{Z}_i,\boldsymbol{X}_i,U_i)\}] + \Delta_{2i}\log[\exp\{-f_{\boldsymbol{\eta}}(\boldsymbol{Z}_i,\boldsymbol{X}_i,U_i)\} - \exp\{-f_{\boldsymbol{\eta}}(\boldsymbol{Z}_i,\boldsymbol{X}_i,V_i)\}] - (1-\Delta_{1i}-\Delta_{2i})f_{\boldsymbol{\eta}}(\boldsymbol{Z}_i,\boldsymbol{X}_i,V_i)\}.$$

Then the score function for $\boldsymbol{\beta}_0$ is

$$\begin{aligned}
\dot{\ell}_{\boldsymbol{\beta}}(\boldsymbol{\eta}_0) &= \frac{\partial m_{(\boldsymbol{\beta},\gamma_0,g_{10},g_{20},\phi_0)}(\boldsymbol{O})}{\partial \boldsymbol{\beta}}\Big|_{\boldsymbol{\beta}=\boldsymbol{\beta}_0} \\
&= \Delta_1 U\boldsymbol{Z}\exp\{-f_{\boldsymbol{\eta}_0}(\boldsymbol{Z},\boldsymbol{X},U)\}f_{\boldsymbol{\eta}_0}(\boldsymbol{Z},\boldsymbol{X},U)\phi'\left(U\exp\{\boldsymbol{\beta}_0^\top\boldsymbol{Z}+g_{10}(\boldsymbol{X})\}\right) \\
&\quad \times \frac{\exp\{\boldsymbol{\beta}_0^\top\boldsymbol{Z}+g_{10}(\boldsymbol{X})\}}{1-\exp\{-f_{\boldsymbol{\eta}_0}(\boldsymbol{Z},\boldsymbol{X},U)\}} + \frac{\Delta_2\boldsymbol{Z}\exp\{\boldsymbol{\beta}_0^\top\boldsymbol{Z}+g_{10}(\boldsymbol{X})\}}{\exp\{-f_{\boldsymbol{\eta}_0}(\boldsymbol{Z},\boldsymbol{X},U)\}-\exp\{-f_{\boldsymbol{\eta}_0}(\boldsymbol{Z},\boldsymbol{X},V)\}} \\
&\quad \times \Big[V\exp\{-f_{\boldsymbol{\eta}_0}(\boldsymbol{Z},\boldsymbol{X},V)\}f_{\boldsymbol{\eta}_0}(\boldsymbol{Z},\boldsymbol{X},V)\phi'\left(V\exp\{\boldsymbol{\beta}_0^\top\boldsymbol{Z}+g_{10}(\boldsymbol{X})\}\right) \\
&\qquad - U\exp\{-f_{\boldsymbol{\eta}_0}(\boldsymbol{Z},\boldsymbol{X},U)\}f_{\boldsymbol{\eta}_0}(\boldsymbol{Z},\boldsymbol{X},U)\phi'\left(U\exp\{\boldsymbol{\beta}_0^\top\boldsymbol{Z}+g_{10}(\boldsymbol{X})\}\right)\Big] \\
&\quad - (1-\Delta_1-\Delta_2)V\boldsymbol{Z}f_{\boldsymbol{\eta}_0}(\boldsymbol{Z},\boldsymbol{X},V)\phi'\left(V\exp\{\boldsymbol{\beta}_0^\top\boldsymbol{Z}+g_{10}(\boldsymbol{X})\}\right)\exp\{\boldsymbol{\beta}_0^\top\boldsymbol{Z}+g_{10}(\boldsymbol{X})\}
\end{aligned}$$

$$
\begin{aligned}
&= \Delta_1 U \boldsymbol{Z} Q_1 \phi'\left(U \exp\{\boldsymbol{\beta}_0^\top \boldsymbol{Z} + g_{10}(\boldsymbol{X})\}\right) \exp\{\boldsymbol{\beta}_0^\top \boldsymbol{Z} + g_{10}(\boldsymbol{X})\} \\
&\quad + \Delta_2 \boldsymbol{Z} \exp\{\boldsymbol{\beta}_0^\top \boldsymbol{Z} + g_{10}(\boldsymbol{X})\} \left[V \phi'\left(V \exp\{\boldsymbol{\beta}_0^\top \boldsymbol{Z} + g_{10}(\boldsymbol{X})\}\right) Q_3 \right. \\
&\qquad\qquad \left. - U \phi'\left(U \exp\{\boldsymbol{\beta}_0^\top \boldsymbol{Z} + g_{10}(\boldsymbol{X})\}\right) Q_2\right] \\
&\quad - (1 - \Delta_1 - \Delta_2) V \boldsymbol{Z} f_{\boldsymbol{\eta}_0}(\boldsymbol{Z}, \boldsymbol{X}, V) \phi'\left(V \exp\{\boldsymbol{\beta}_0^\top \boldsymbol{Z} + g_{10}(\boldsymbol{X})\}\right) \exp\{\boldsymbol{\beta}_0^\top \boldsymbol{Z} + g_{10}(\boldsymbol{X})\} \\
&= U \boldsymbol{Z} (\Delta_1 Q_1 - \Delta_2 Q_2) \phi'\left(U \exp\{\boldsymbol{\beta}_0^\top \boldsymbol{Z} + g_{10}(\boldsymbol{X})\}\right) \exp\{\boldsymbol{\beta}_0^\top \boldsymbol{Z} + g_{10}(\boldsymbol{X})\} \\
&\quad + V \boldsymbol{Z} \{\Delta_2 Q_3 - (1 - \Delta_1 - \Delta_2) f_{\boldsymbol{\eta}_0}(\boldsymbol{Z}, \boldsymbol{X}, V)\} \\
&\qquad \times \phi'\left(V \exp\{\boldsymbol{\beta}_0^\top \boldsymbol{Z} + g_{10}(\boldsymbol{X})\}\right) \exp\{\boldsymbol{\beta}_0^\top \boldsymbol{Z} + g_{10}(\boldsymbol{X})\},
\end{aligned}
$$

where ϕ' is the first derivative function of function ϕ,

$$
Q_1 = \frac{\exp\{-f_{\boldsymbol{\eta}_0}(\boldsymbol{Z}, \boldsymbol{X}, U)\} f_{\boldsymbol{\eta}_0}(\boldsymbol{Z}, \boldsymbol{X}, U)}{1 - \exp\{-f_{\boldsymbol{\eta}_0}(\boldsymbol{Z}, \boldsymbol{X}, U)\}},
$$

$$
Q_2 = \frac{\exp\{-f_{\boldsymbol{\eta}_0}(\boldsymbol{Z}, \boldsymbol{X}, U)\} f_{\boldsymbol{\eta}_0}(\boldsymbol{Z}, \boldsymbol{X}, U)}{\exp\{-f_{\boldsymbol{\eta}_0}(\boldsymbol{Z}, \boldsymbol{X}, U)\} - \exp\{-f_{\boldsymbol{\eta}_0}(\boldsymbol{Z}, \boldsymbol{X}, V)\}},
$$

and

$$
Q_3 = \frac{\exp\{-f_{\boldsymbol{\eta}_0}(\boldsymbol{Z}, \boldsymbol{X}, V)\} f_{\boldsymbol{\eta}_0}(\boldsymbol{Z}, \boldsymbol{X}, V)}{\exp\{-f_{\boldsymbol{\eta}_0}(\boldsymbol{Z}, \boldsymbol{X}, U)\} - \exp\{-f_{\boldsymbol{\eta}_0}(\boldsymbol{Z}, \boldsymbol{X}, V)\}}.
$$

The score function for $\boldsymbol{\gamma}_0$ is

$$
\begin{aligned}
\dot{\ell}_{\boldsymbol{\gamma}}(\boldsymbol{\eta}_0) &= \frac{\partial m_{(\boldsymbol{\beta}_0, \boldsymbol{\gamma}, g_{10}, g_{20}, \phi_0)}(\boldsymbol{O})}{\partial \boldsymbol{\gamma}}\Big|_{\boldsymbol{\gamma}=\boldsymbol{\gamma}_0} \\
&= \frac{\Delta_1 \boldsymbol{Z} \exp\{-f_{\boldsymbol{\eta}_0}(\boldsymbol{Z}, \boldsymbol{X}, U)\} f_{\boldsymbol{\eta}_0}(\boldsymbol{Z}, \boldsymbol{X}, U)}{1 - \exp\{-f_{\boldsymbol{\eta}_0}(\boldsymbol{Z}, \boldsymbol{X}, U)\}} \\
&\quad + \frac{\Delta_2 \boldsymbol{Z} [\exp\{-f_{\boldsymbol{\eta}_0}(\boldsymbol{Z}, \boldsymbol{X}, V)\} f_{\boldsymbol{\eta}_0}(\boldsymbol{Z}, \boldsymbol{X}, V) - \exp\{-f_{\boldsymbol{\eta}_0}(\boldsymbol{Z}, \boldsymbol{X}, U)\} f_{\boldsymbol{\eta}_0}(\boldsymbol{Z}, \boldsymbol{X}, U)]}{\exp\{-f_{\boldsymbol{\eta}_0}(\boldsymbol{Z}, \boldsymbol{X}, U)\} - \exp\{-f_{\boldsymbol{\eta}_0}(\boldsymbol{Z}, \boldsymbol{X}, V)\}} \\
&\quad - (1 - \Delta_1 - \Delta_2) \boldsymbol{Z} f_{\boldsymbol{\eta}_0}(\boldsymbol{Z}, \boldsymbol{X}, V) \\
&= \boldsymbol{Z} \{\Delta_1 Q_1 + \Delta_2 (Q_3 - Q_2) - (1 - \Delta_1 - \Delta_2) f_{\boldsymbol{\eta}_0}(\boldsymbol{Z}, \boldsymbol{X}, V)\}.
\end{aligned}
$$

So the score function for $\boldsymbol{\theta}_0$ is $\dot{\ell}_{\boldsymbol{\theta}}(\boldsymbol{\eta}_0) = \left(\dot{\ell}_{\boldsymbol{\beta}}^\top(\boldsymbol{\eta}_0), \dot{\ell}_{\boldsymbol{\gamma}}^\top(\boldsymbol{\eta}_0)\right)^\top$

Consider the family of functions $(g_{s_1}, g_{s_2}, \Lambda_q)$ such that $g_{1s_1}|_{s_1=0} = g_{10}$, $g_{2s_2}|_{s_2=0} = g_{20}$ and $\phi_q|_{q=0} = \phi_0$, with $\frac{\partial g_{1s_1}}{\partial s_1}\Big|_{s_1=0} = a$, $\frac{\partial g_{2s_2}}{\partial s_2}\Big|_{s_2=0} = b$, and $\frac{\partial \phi_q}{\partial q}\Big|_{q=0} = c$. The score operators

for g_{10}, g_{20} and ϕ_0 are defined as

$$
\begin{aligned}
&\dot{\ell}_{g_1}(\boldsymbol{\eta}_0)[a] = \frac{\partial m_{(\boldsymbol{\beta}_0,\gamma_0,g_{1s_1},g_{2s_2},\phi_q)}(\boldsymbol{O})}{\partial s_1}\Big|_{s_1=s_2=q=0} \\
&= \Delta_1 U \exp\{-f_{\boldsymbol{\eta}_0}(\boldsymbol{Z},\boldsymbol{X},U)\} f_{\boldsymbol{\eta}_0}(\boldsymbol{Z},\boldsymbol{X},U)\phi'\left(U\exp\{\boldsymbol{\beta}_0^\top \boldsymbol{Z} + g_{10}(\boldsymbol{X})\}\right) \\
&\quad \times \frac{\exp\{\boldsymbol{\beta}_0^\top \boldsymbol{Z} + g_{10}(\boldsymbol{X})\}a(\boldsymbol{X})}{1-\exp\{-f_{\boldsymbol{\eta}_0}(\boldsymbol{Z},\boldsymbol{X},U)\}} \\
&\quad + \frac{\Delta_2 \exp\{\boldsymbol{\beta}_0^\top \boldsymbol{Z} + g_{10}(\boldsymbol{X})\}a(\boldsymbol{X})}{\exp\{-f_{\boldsymbol{\eta}_0}(\boldsymbol{Z},\boldsymbol{X},U)\} - \exp\{-f_{\boldsymbol{\eta}_0}(\boldsymbol{Z},\boldsymbol{X},V)\}} \\
&\quad \times \Big[V\exp\{-f_{\boldsymbol{\eta}_0}(\boldsymbol{Z},\boldsymbol{X},V)\} f_{\boldsymbol{\eta}_0}(\boldsymbol{Z},\boldsymbol{X},V)\phi'\left(V\exp\{\boldsymbol{\beta}_0^\top \boldsymbol{Z} + g_{10}(\boldsymbol{X})\}\right) \\
&\qquad - U\exp\{-f_{\boldsymbol{\eta}_0}(\boldsymbol{Z},\boldsymbol{X},U)\} f_{\boldsymbol{\eta}_0}(\boldsymbol{Z},\boldsymbol{X},U)\phi'\left(U\exp\{\boldsymbol{\beta}_0^\top \boldsymbol{Z} + g_{10}(\boldsymbol{X})\}\right)\Big] \\
&\quad - (1-\Delta_1-\Delta_2)V f_{\boldsymbol{\eta}_0}(\boldsymbol{Z},\boldsymbol{X},V)\phi'\left(V\exp\{\boldsymbol{\beta}_0^\top \boldsymbol{Z} + g_{10}(\boldsymbol{X})\}\right)\exp\{\boldsymbol{\beta}_0^\top \boldsymbol{Z} + g_{10}(\boldsymbol{X})\}a(\boldsymbol{X}) \\
&= U(\Delta_1 Q_1 - \Delta_2 Q_2)\phi'\left(U\exp\{\boldsymbol{\beta}_0^\top \boldsymbol{Z} + g_{10}(\boldsymbol{X})\}\right)\exp\{\boldsymbol{\beta}_0^\top \boldsymbol{Z} + g_{10}(\boldsymbol{X})\}a(\boldsymbol{X}) \\
&\quad + V\{\Delta_2 Q_3 - (1-\Delta_1-\Delta_2) f_{\boldsymbol{\eta}_0}(\boldsymbol{Z},\boldsymbol{X},V)\} \\
&\quad \times \phi'\left(V\exp\{\boldsymbol{\beta}_0^\top \boldsymbol{Z} + g_{10}(\boldsymbol{X})\}\right)\exp\{\boldsymbol{\beta}_0^\top \boldsymbol{Z} + g_{10}(\boldsymbol{X})\}a(\boldsymbol{X}),
\end{aligned}
$$

$$
\begin{aligned}
\dot{\ell}_{g_2}(\boldsymbol{\eta}_0)[b] &= \frac{\partial m_{(\boldsymbol{\beta}_0,\gamma_0,g_{1s_1},g_{2s_2},\phi_q)}(\boldsymbol{O})}{\partial s_2}\Big|_{s_1=s_2=q=0} \\
&= \frac{\Delta_1 \exp\{-f_{\boldsymbol{\eta}_0}(\boldsymbol{Z},\boldsymbol{X},U)\} f_{\boldsymbol{\eta}_0}(\boldsymbol{Z},\boldsymbol{X},U) b(\boldsymbol{X})}{1-\exp\{-f_{\boldsymbol{\eta}_0}(\boldsymbol{Z},\boldsymbol{X},U)\}} \\
&\quad + \frac{\Delta_2 b(\boldsymbol{X})}{\exp\{-f_{\boldsymbol{\eta}_0}(\boldsymbol{Z},\boldsymbol{X},U)\} - \exp\{-f_{\boldsymbol{\eta}_0}(\boldsymbol{Z},\boldsymbol{X},V)\}} \\
&\qquad \times [\exp\{-f_{\boldsymbol{\eta}_0}(\boldsymbol{Z},\boldsymbol{X},V)\} f_{\boldsymbol{\eta}_0}(\boldsymbol{Z},\boldsymbol{X},V) - \exp\{-f_{\boldsymbol{\eta}_0}(\boldsymbol{Z},\boldsymbol{X},U)\} f_{\boldsymbol{\eta}_0}(\boldsymbol{Z},\boldsymbol{X},U)] \\
&\quad - (1-\Delta_1-\Delta_2) f_{\boldsymbol{\eta}_0}(\boldsymbol{Z},\boldsymbol{X},V) b(\boldsymbol{X}) \\
&= b(\boldsymbol{X})\{\Delta_1 Q_1 + \Delta_2(Q_3 - Q_2) - (1-\Delta_1-\Delta_2) f_{\boldsymbol{\eta}_0}(\boldsymbol{Z},\boldsymbol{X},V)\},
\end{aligned}
$$

and

$$
\begin{aligned}
\dot{\ell}_{\phi}(\boldsymbol{\eta}_0)[c] &= \frac{\partial m_{(\boldsymbol{\beta}_0,\gamma_0,g_{1s_1},g_{2s_2},\phi_q)}(\boldsymbol{O})}{\partial q}\Big|_{s_1=s_2=q=0} \\
&= \frac{\Delta_1 \exp\{-f_{\boldsymbol{\eta}_0}(\boldsymbol{Z},\boldsymbol{X},U)\} f_{\boldsymbol{\eta}_0}(\boldsymbol{Z},\boldsymbol{X},U) c\left(U\exp\{\boldsymbol{\beta}_0^\top \boldsymbol{Z} + g_{10}(\boldsymbol{X})\}\right)}{1-\exp\{-f_{\boldsymbol{\eta}_0}(\boldsymbol{Z},\boldsymbol{X},U)\}} \\
&\quad + \frac{\Delta_2 \exp\{-f_{\boldsymbol{\eta}_0}(\boldsymbol{Z},\boldsymbol{X},V)\} f_{\boldsymbol{\eta}_0}(\boldsymbol{Z},\boldsymbol{X},V) c\left(V\exp\{\boldsymbol{\beta}_0^\top \boldsymbol{Z} + g_{10}(\boldsymbol{X})\}\right)}{\exp\{-f_{\boldsymbol{\eta}_0}(\boldsymbol{Z},\boldsymbol{X},U)\}-\exp\{-f_{\boldsymbol{\eta}_0}(\boldsymbol{Z},\boldsymbol{X},V)\}} \\
&\quad - \frac{\Delta_2 \exp\{-f_{\boldsymbol{\eta}_0}(\boldsymbol{Z},\boldsymbol{X},U)\} f_{\boldsymbol{\eta}_0}(\boldsymbol{Z},\boldsymbol{X},U) c\left(U\exp\{\boldsymbol{\beta}_0^\top \boldsymbol{Z} + g_{10}(\boldsymbol{X})\}\right)}{\exp\{-f_{\boldsymbol{\eta}_0}(\boldsymbol{Z},\boldsymbol{X},U)\}-\exp\{-f_{\boldsymbol{\eta}_0}(\boldsymbol{Z},\boldsymbol{X},V)\}} \\
&\quad - (1-\Delta_1-\Delta_2) f_{\boldsymbol{\eta}_0}(\boldsymbol{Z},\boldsymbol{X},V) c\left(U\exp\{\boldsymbol{\beta}_0^\top \boldsymbol{Z} + g_{10}(\boldsymbol{X})\}\right) \\
&= (\Delta_1 Q_1 - \Delta_2 Q_2) c\left(U\exp\{\boldsymbol{\beta}_0^\top \boldsymbol{Z} + g_{10}(\boldsymbol{X})\}\right) \\
&\quad + \{\Delta_2 Q_3 - (1-\Delta_1-\Delta_2) f_{\boldsymbol{\eta}_0}(\boldsymbol{Z},\boldsymbol{X},V)\} c\left(V\exp\{\boldsymbol{\beta}_0^\top \boldsymbol{Z} + g_{10}(\boldsymbol{X})\}\right),
\end{aligned}
$$

For $\boldsymbol{a} = (a_1,\ldots,a_{2p})^\top$, $\boldsymbol{b} = (b_1,\ldots,b_{2p})^\top \in \overline{\mathbb{S}}_{g_0}^{2p}$, define $\dot{\ell}_{g_1}(\boldsymbol{\eta}_0)[\boldsymbol{a}] = (\dot{\ell}_{g_1}(\boldsymbol{\eta}_0)[a_1],\ldots,\dot{\ell}_{g_1}(\boldsymbol{\eta}_0)[a_{2p}])^\top$ and $\dot{\ell}_{g_2}(\boldsymbol{\eta}_0)[\boldsymbol{b}] = (\dot{\ell}_{g_2}(\boldsymbol{\eta}_0)[b_1],\ldots,\dot{\ell}_{g_2}(\boldsymbol{\eta}_0)[b_{2p}])^\top$. Similarly, for $\boldsymbol{c} = (c_1,\ldots,c_{2p})^\top \in \overline{\mathbb{S}}_{\phi_0}^{2p}$, define $\dot{\ell}_{\phi}(\boldsymbol{\eta}_0)[\boldsymbol{c}] = (\dot{\ell}_{\phi}(\boldsymbol{\eta}_0)[c_1],\ldots,\dot{\ell}_{\phi}(\boldsymbol{\eta}_0)[c_{2p}])^\top$. We write $a_n \lesssim b_n$ or $b_n \gtrsim a_n$ if $\limsup_{n\to\infty} a_n/b_n \le c_0$ for some positive c_0, and denote $a_n \asymp b_n$ if $a_n \lesssim b_n$ and $a_n \gtrsim b_n$ hold.

To prove the theorems, we need some lemmas.

Lemma 1. *Under conditions* (C1)-(C6), *there exist two positive constants* C_1 *and* C_2 *with* $C_1 \le C_2$ *such that*

$$
C_1 d^2(\boldsymbol{\eta},\boldsymbol{\eta}_0) \le L(\boldsymbol{\eta}) - L(\boldsymbol{\eta}_0) \le C_2 d^2(\boldsymbol{\eta},\boldsymbol{\eta}_0)
$$

for all $\boldsymbol{\eta} \in \{\boldsymbol{\eta} : \ d(\boldsymbol{\eta},\boldsymbol{\eta}_0) < C^*\}$ *with some finite positive constant* C^*.

Lemma 2. *Assume that* $|h_{\boldsymbol{\eta}}(\boldsymbol{O})| \le B$ *a.s. and* $B \ge 1$. *Let* $C_0 = \max\{C_2 C^{*2}/C_1, B\}$. *Then, for each* $n \ge 1$,

$$
\begin{aligned}
&P\left\{\exists \boldsymbol{\eta} \in \Theta : \mathbb{E}\{h_{\boldsymbol{\eta}}(\boldsymbol{O})\} - \frac{1}{n}\sum_{i=1}^{n} h_{\boldsymbol{\eta}}(\boldsymbol{O}_i) \ge \epsilon(\alpha + \beta + \mathbb{E}\{h_{\boldsymbol{\eta}}(\boldsymbol{O})\}\right\} \\
&\le 14 \sup_{\boldsymbol{o}_1^n} \mathcal{N}_1\left(\frac{\epsilon\beta}{8}, \mathcal{H}_\Theta, \boldsymbol{o}_1^n\right) \exp\left(-\frac{\epsilon^2(1-\epsilon)\alpha n}{40 B^2 C_0 (1+\epsilon)}\right),
\end{aligned} \tag{1}
$$

where $\mathcal{H}_\Theta = \{h_{\boldsymbol{\eta}} : \boldsymbol{\eta} \in \Theta\}$, $\boldsymbol{o}_1^n = (\boldsymbol{o}_1,\ldots,\boldsymbol{o}_n)^T$, $\boldsymbol{o}_i \in \mathbb{R}^{p+r+4}$ $(i = 1,\ldots,n)$, $\alpha > 0$, $\beta > 0$ *and* $0 < \epsilon \le 1/2$.

Proof of Lemma 1.

With some simple algebra, we get

$$
\begin{aligned}
L(\boldsymbol{\eta}) - L(\boldsymbol{\eta}_0) &= \mathbb{E}\left[\Delta_1 \log \frac{1-\exp\{-f_{\boldsymbol{\eta}_0}(\boldsymbol{Z},\boldsymbol{X},U)\}}{1-\exp\{-f_{\boldsymbol{\eta}}(\boldsymbol{Z},\boldsymbol{X},U)\}}\right. \\
&\quad +\Delta_2 \log \frac{\exp\{-f_{\boldsymbol{\eta}_0}(\boldsymbol{Z},\boldsymbol{X},U)\}-\exp\{-f_{\boldsymbol{\eta}_0}(\boldsymbol{Z},\boldsymbol{X},V)\}}{\exp\{-f_{\boldsymbol{\eta}}(\boldsymbol{Z},\boldsymbol{X},U)\}-\exp\{-f_{\boldsymbol{\eta}}(\boldsymbol{Z},\boldsymbol{X},V)\}} \\
&\quad \left.+\Delta_3 \log \frac{\exp\{-f_{\boldsymbol{\eta}_0}(\boldsymbol{Z},\boldsymbol{X},V)\}}{\exp\{-f_{\boldsymbol{\eta}}(\boldsymbol{Z},\boldsymbol{X},V)\}}\right] \\
&= \mathbb{E}\left\{[1-\exp\{-f_{\boldsymbol{\eta}_0}(\boldsymbol{Z},\boldsymbol{X},U)\}] \log \frac{1-\exp\{-f_{\boldsymbol{\eta}_0}(\boldsymbol{Z},\boldsymbol{X},U)\}}{1-\exp\{-f_{\boldsymbol{\eta}}(\boldsymbol{Z},\boldsymbol{X},U)\}}\right. \\
&\quad +[\exp\{-f_{\boldsymbol{\eta}_0}(\boldsymbol{Z},\boldsymbol{X},U)\}-\exp\{-f_{\boldsymbol{\eta}_0}(\boldsymbol{Z},\boldsymbol{X},V)\}] \\
&\qquad \times \log \frac{\exp\{-f_{\boldsymbol{\eta}_0}(\boldsymbol{Z},\boldsymbol{X},U)\}-\exp\{-f_{\boldsymbol{\eta}_0}(\boldsymbol{Z},\boldsymbol{X},V)\}}{\exp\{-f_{\boldsymbol{\eta}}(\boldsymbol{Z},\boldsymbol{X},U)\}-\exp\{-f_{\boldsymbol{\eta}}(\boldsymbol{Z},\boldsymbol{X},V)\}} \\
&\quad \left.+\exp\{-f_{\boldsymbol{\eta}_0}(\boldsymbol{Z},\boldsymbol{X},V)\} \log \frac{\exp\{-f_{\boldsymbol{\eta}_0}(\boldsymbol{Z},\boldsymbol{X},V)\}}{\exp\{-f_{\boldsymbol{\eta}}(\boldsymbol{Z},\boldsymbol{X},V)\}}\right\} \\
&= \mathbb{E}\left\{[1-\exp\{-f_{\boldsymbol{\eta}}(\boldsymbol{Z},\boldsymbol{X},U)\}] h\left(\frac{1-\exp\{-f_{\boldsymbol{\eta}_0}(\boldsymbol{Z},\boldsymbol{X},U)\}}{1-\exp\{-f_{\boldsymbol{\eta}}(\boldsymbol{Z},\boldsymbol{X},U)\}}\right)\right. \\
&\quad +[\exp\{-f_{\boldsymbol{\eta}}(\boldsymbol{Z},\boldsymbol{X},U)\}-\exp\{-f_{\boldsymbol{\eta}}(\boldsymbol{Z},\boldsymbol{X},V)\}] \\
&\qquad \times h\left(\frac{\exp\{-f_{\boldsymbol{\eta}_0}(\boldsymbol{Z},\boldsymbol{X},U)\}-\exp\{-f_{\boldsymbol{\eta}_0}(\boldsymbol{Z},\boldsymbol{X},V)\}}{\exp\{-f_{\boldsymbol{\eta}}(\boldsymbol{Z},\boldsymbol{X},U)\}-\exp\{-f_{\boldsymbol{\eta}}(\boldsymbol{Z},\boldsymbol{X},V)\}}\right) \\
&\quad \left.+\exp\{-f_{\boldsymbol{\eta}}(\boldsymbol{Z},\boldsymbol{X},V)\} h\left(\frac{\exp\{-f_{\boldsymbol{\eta}_0}(\boldsymbol{Z},\boldsymbol{X},V)\}}{\exp\{-f_{\boldsymbol{\eta}}(\boldsymbol{Z},\boldsymbol{X},V)\}}\right)\right\},
\end{aligned}
$$

where $(x-1)^2/4 \le h(x) = x\log x - x + 1 \le (x-1)^2$ for $0 \le x \le 5$. And then

$$
\begin{aligned}
& L(\boldsymbol{\eta}) - L(\boldsymbol{\eta}_0) \\
\le\ & \mathbb{E}\left\{[1-\exp\{-f_{\boldsymbol{\eta}}(\boldsymbol{Z},\boldsymbol{X},U)\}] \times \left[\frac{1-\exp\{-f_{\boldsymbol{\eta}_0}(\boldsymbol{Z},\boldsymbol{X},U)\}}{1-\exp\{-f_{\boldsymbol{\eta}}(\boldsymbol{Z},\boldsymbol{X},U)\}} - 1\right]^2\right. \\
& +[\exp\{-f_{\boldsymbol{\eta}}(\boldsymbol{Z},\boldsymbol{X},U)\}-\exp\{-f_{\boldsymbol{\eta}}(\boldsymbol{Z},\boldsymbol{X},V)\}] \\
& \quad \times \left[\frac{\exp\{-f_{\boldsymbol{\eta}_0}(\boldsymbol{Z},\boldsymbol{X},U)\}-\exp\{-f_{\boldsymbol{\eta}_0}(\boldsymbol{Z},\boldsymbol{X},V)\}}{\exp\{-f_{\boldsymbol{\eta}}(\boldsymbol{Z},\boldsymbol{X},U)\}-\exp\{-f_{\boldsymbol{\eta}}(\boldsymbol{Z},\boldsymbol{X},V)\}} - 1\right]^2 \\
& \left.+\exp\{-f_{\boldsymbol{\eta}}(\boldsymbol{Z},\boldsymbol{X},V)\} \times \left[\frac{\exp\{-f_{\boldsymbol{\eta}_0}(\boldsymbol{Z},\boldsymbol{X},V)\}}{\exp\{-f_{\boldsymbol{\eta}}(\boldsymbol{Z},\boldsymbol{X},V)\}} - 1\right]^2\right\}
\end{aligned}
$$

$$
\begin{aligned}
&= \mathbb{E}\Bigg\{ \frac{[\exp\{-f_{\boldsymbol{\eta}}(\boldsymbol{Z},\boldsymbol{X},U)\} - \exp\{-f_{\boldsymbol{\eta}_0}(\boldsymbol{Z},\boldsymbol{X},U)\}]^2}{1-\exp\{-f_{\boldsymbol{\eta}}(\boldsymbol{Z},\boldsymbol{X},U)\}} \\
&\quad + \frac{1}{\exp\{-f_{\boldsymbol{\eta}}(\boldsymbol{Z},\boldsymbol{X},U)\} - \exp\{-f_{\boldsymbol{\eta}}(\boldsymbol{Z},\boldsymbol{X},V)\}} \\
&\quad \times [\exp\{-f_{\boldsymbol{\eta}_0}(\boldsymbol{Z},\boldsymbol{X},U)\} - \exp\{-f_{\boldsymbol{\eta}_0}(\boldsymbol{Z},\boldsymbol{X},V)\} \\
&\qquad - \exp\{-f_{\boldsymbol{\eta}}(\boldsymbol{Z},\boldsymbol{X},U)\} + \exp\{-f_{\boldsymbol{\eta}}(\boldsymbol{Z},\boldsymbol{X},V)\}]^2 \\
&\quad + \frac{[\exp\{-f_{\boldsymbol{\eta}_0}(\boldsymbol{Z},\boldsymbol{X},V)\} - \exp\{-f_{\boldsymbol{\eta}}(\boldsymbol{Z},\boldsymbol{X},V)\}]^2}{\exp\{-f_{\boldsymbol{\eta}}(\boldsymbol{Z},\boldsymbol{X},V)\}} \Bigg\} \\
&\lesssim d^2(\boldsymbol{\eta},\boldsymbol{\eta}_0).
\end{aligned}
$$

The last inequality holds by Taylor expansion and some bounded conditions. On the other hand, combining conditions (C1)-(C6) and Theorem 25.86 of Van der Vaart (2000), some algebra yields that

$$
\begin{aligned}
L(\boldsymbol{\eta}) - L(\boldsymbol{\eta}_0) &\geq \frac{1}{4}\mathbb{E}\Bigg\{ \frac{[\exp\{-f_{\boldsymbol{\eta}}(\boldsymbol{Z},\boldsymbol{X},U)\} - \exp\{-f_{\boldsymbol{\eta}_0}(\boldsymbol{Z},\boldsymbol{X},U)\}]^2}{1-\exp\{-f_{\boldsymbol{\eta}}(\boldsymbol{Z},\boldsymbol{X},U)\}} \\
&\quad + \frac{1}{\exp\{-f_{\boldsymbol{\eta}}(\boldsymbol{Z},\boldsymbol{X},U)\} - \exp\{-f_{\boldsymbol{\eta}}(\boldsymbol{Z},\boldsymbol{X},V)\}} \big[\exp\{-f_{\boldsymbol{\eta}_0}(\boldsymbol{Z},\boldsymbol{X},U)\} \\
&\qquad - \exp\{-f_{\boldsymbol{\eta}_0}(\boldsymbol{Z},\boldsymbol{X},V)\} - \exp\{-f_{\boldsymbol{\eta}}(\boldsymbol{Z},\boldsymbol{X},U)\} + \exp\{-f_{\boldsymbol{\eta}}(\boldsymbol{Z},\boldsymbol{X},V)\}\big]^2 \\
&\quad + \frac{[\exp\{-f_{\boldsymbol{\eta}_0}(\boldsymbol{Z},\boldsymbol{X},V)\} - \exp\{-f_{\boldsymbol{\eta}}(\boldsymbol{Z},\boldsymbol{X},V)\}]^2}{\exp\{-f_{\boldsymbol{\eta}}(\boldsymbol{Z},\boldsymbol{X},V)\}} \Bigg\} \\
&\geq \frac{1}{4}\mathbb{E}\Bigg\{ \frac{[\exp\{-f_{\boldsymbol{\eta}}(\boldsymbol{Z},\boldsymbol{X},U)\} - \exp\{-f_{\boldsymbol{\eta}_0}(\boldsymbol{Z},\boldsymbol{X},U)\}]^2}{1-\exp\{-f_{\boldsymbol{\eta}}(\boldsymbol{Z},\boldsymbol{X},U)\}} \\
&\qquad + \frac{[\exp\{-f_{\boldsymbol{\eta}_0}(\boldsymbol{Z},\boldsymbol{X},V)\} - \exp\{-f_{\boldsymbol{\eta}}(\boldsymbol{Z},\boldsymbol{X},V)\}]^2}{\exp\{-f_{\boldsymbol{\eta}}(\boldsymbol{Z},\boldsymbol{X},V)\}} \Bigg\} \\
&\gtrsim \mathbb{E}\Big[\Big\{ (\boldsymbol{\gamma}-\boldsymbol{\gamma}_0)^\top \boldsymbol{Z} + (g_2 - g_{20})(\boldsymbol{X}) \\
&\qquad + \big(\phi(U\exp\{\boldsymbol{\beta}^\top\boldsymbol{Z} + g_1(\boldsymbol{X})\}) - \phi_0(U\exp\{\boldsymbol{\beta}_0^\top\boldsymbol{Z} + g_{10}(\boldsymbol{X})\})\big) \Big\}^2 \\
&\quad + \Big\{ (\boldsymbol{\gamma}-\boldsymbol{\gamma}_0)^\top \boldsymbol{Z} + (g_2 - g_{20})(\boldsymbol{X}) \\
&\qquad + \big(\phi(V\exp\{\boldsymbol{\beta}^\top\boldsymbol{Z} + g_1(\boldsymbol{X})\}) - \phi_0(V\exp\{\boldsymbol{\beta}_0^\top\boldsymbol{Z} + g_{10}(\boldsymbol{X})\})\big) \Big\}^2 \Big] \\
&\gtrsim \|\boldsymbol{\gamma}-\boldsymbol{\gamma}_0\|^2 + \|g_2 - g_{20}\|^2_{L^2([0,1]^r)} \\
&\quad + \mathbb{E}\left\{\phi(U\exp\{\boldsymbol{\beta}^\top\boldsymbol{Z} + g_1(\boldsymbol{X})\}) - \phi_0(U\exp\{\boldsymbol{\beta}_0^\top\boldsymbol{Z} + g_{10}(\boldsymbol{X})\})\right\}^2 \\
&\quad + \mathbb{E}\left\{\phi(V\exp\{\boldsymbol{\beta}^\top\boldsymbol{Z} + g_1(\boldsymbol{X})\}) - \phi_0(V\exp\{\boldsymbol{\beta}_0^\top\boldsymbol{Z} + g_{10}(\boldsymbol{X})\})\right\}^2
\end{aligned}
$$

$$
\begin{aligned}
&\gtrsim \|\boldsymbol{\gamma}-\boldsymbol{\gamma}_0\|^2+\|g_2-g_{20}\|^2_{L^2([0,1]^r)}\\
&\quad+\mathbb{E}\left\{\phi(U\exp\{\boldsymbol{\beta}^\top\boldsymbol{Z}+g_1(\boldsymbol{X})\})-\phi(U\exp\{\boldsymbol{\beta}_0^\top\boldsymbol{Z}+g_{10}(\boldsymbol{X})\})\right.\\
&\qquad\left.+\phi(V\exp\{\boldsymbol{\beta}_0^\top\boldsymbol{Z}+g_{10}(\boldsymbol{X})\})-\phi_0(V\exp\{\boldsymbol{\beta}_0^\top\boldsymbol{Z}+g_{10}(\boldsymbol{X})\})\right\}^2\\
&\quad+\mathbb{E}\left\{\phi(U\exp\{\boldsymbol{\beta}^\top\boldsymbol{Z}+g_1(\boldsymbol{X})\})-\phi(U\exp\{\boldsymbol{\beta}_0^\top\boldsymbol{Z}+g_{10}(\boldsymbol{X})\})\right.\\
&\qquad\left.+\phi(U\exp\{\boldsymbol{\beta}_0^\top\boldsymbol{Z}+g_{10}(\boldsymbol{X})\})-\phi_0(U\exp\{\boldsymbol{\beta}_0^\top\boldsymbol{Z}+g_{10}(\boldsymbol{X})\})\right\}^2\\
&\gtrsim \|\boldsymbol{\gamma}-\boldsymbol{\gamma}_0\|^2+\|g_2-g_{20}\|^2_{L^2([0,1]^r)}\\
&\quad+\mathbb{E}\left\{\phi(U\exp\{\boldsymbol{\beta}^\top\boldsymbol{Z}+g_1(\boldsymbol{X})\})-\phi(U\exp\{\boldsymbol{\beta}_0^\top\boldsymbol{Z}+g_{10}(\boldsymbol{X})\})\right\}^2\\
&\quad+\mathbb{E}\left\{\phi(U\exp\{\boldsymbol{\beta}_0^\top\boldsymbol{Z}+g_{10}(\boldsymbol{X})\})-\phi_0(U\exp\{\boldsymbol{\beta}_0^\top\boldsymbol{Z}+g_{10}(\boldsymbol{X})\})\right\}^2\\
&\quad+\mathbb{E}\left\{\phi(V\exp\{\boldsymbol{\beta}^\top\boldsymbol{Z}+g_1(\boldsymbol{X})\})-\phi(V\exp\{\boldsymbol{\beta}_0^\top\boldsymbol{Z}+g_{10}(\boldsymbol{X})\})\right\}^2\\
&\quad+\mathbb{E}\left\{\phi(V\exp\{\boldsymbol{\beta}_0^\top\boldsymbol{Z}+g_{10}(\boldsymbol{X})\})-\phi_0(V\exp\{\boldsymbol{\beta}_0^\top\boldsymbol{Z}+g_{10}(\boldsymbol{X})\})\right\}^2\\
&\gtrsim \|\boldsymbol{\gamma}-\boldsymbol{\gamma}_0\|^2+\|g_2-g_{20}\|^2_{L^2([0,1]^r)}+\|\phi-\phi_0\|^2_\Phi+\mathbb{E}\left\{(\boldsymbol{\beta}-\boldsymbol{\beta}_0)^\top\boldsymbol{Z}+(g_1-g_{10})(\boldsymbol{X})\right\}^2\\
&\gtrsim \|\boldsymbol{\gamma}-\boldsymbol{\gamma}_0\|^2+\|g_2-g_{20}\|^2_{L^2([0,1]^r)}+\|\phi-\phi_0\|^2_\Phi+\|\boldsymbol{\beta}-\boldsymbol{\beta}_0\|^2+\|g_1-g_{10}\|^2_{L^2([0,1]^r)}=d^2\left(\boldsymbol{\eta},\boldsymbol{\eta}_0\right)
\end{aligned}
$$

Thus, it follows that $L(\boldsymbol{\eta})-L(\boldsymbol{\eta}_0)\asymp d^2(\boldsymbol{\eta},\boldsymbol{\eta}_0)$.

<u>Proof of Lemma 2</u>.

Motivated by the proof of Theorem 11.4 in Györfi et al. (2002), we show the theorem through the following steps.

Step 1: Symmetrizing the analysis using a ghost sample.

Consider $\boldsymbol{\eta}_n\in\Theta$ depending on $\boldsymbol{O}_1^n$ such that

$$
\mathbb{E}\{h_{\boldsymbol{\eta}_n}(\boldsymbol{O})|\boldsymbol{O}_1^n\}-\frac{1}{n}\sum_{i=1}^n h_{\boldsymbol{\eta}_n}(\boldsymbol{O}_i)\geq\epsilon\left(\alpha+\beta+\mathbb{E}\{h_{\boldsymbol{\eta}_n}(\boldsymbol{O})|\boldsymbol{O}_1^n\}\right).
$$

According to the proof of Lemma 1, it is easy to get

$$
\mathbb{E}\{h^2_{\boldsymbol{\eta}}(\boldsymbol{O})\}=\mathbb{E}\{m_{\boldsymbol{\eta}_0}(\boldsymbol{O})-m_{\boldsymbol{\eta}}(\boldsymbol{O})\}^2\leq C_2d^4(\boldsymbol{\eta},\boldsymbol{\eta}_0)\leq\frac{C_2C^{*2}}{C_1}\mathbb{E}\{h_{\boldsymbol{\eta}}(\boldsymbol{O})\}\leq C_0\mathbb{E}\{h_{\boldsymbol{\eta}}(\boldsymbol{O})\}.
$$

It follows that $\mathrm{var}\{h_{\boldsymbol{\eta}_n}(\boldsymbol{O})|\boldsymbol{O}_1^n\}\leq\mathbb{E}\{h^2_{\boldsymbol{\eta}_n}(\boldsymbol{O})|\boldsymbol{O}_1^n\}\leq C_0\mathbb{E}\{h_{\boldsymbol{\eta}_n}(\boldsymbol{O})|\boldsymbol{O}_1^n\}$ *a.s.* By Cheby-

shev's inequality, we have

$$
\begin{aligned}
&P\left\{\mathbb{E}\{h_{\boldsymbol{\eta}_n}(\boldsymbol{O})|\boldsymbol{O}_1^n\}-\frac{1}{n}\sum_{i=1}^n h_{\boldsymbol{\eta}_n}(\boldsymbol{O}_i')>\frac{\epsilon}{2}(\alpha+\beta)+\frac{\epsilon}{2}\mathbb{E}\{h_{\boldsymbol{\eta}_n}(\boldsymbol{O})\Big|\boldsymbol{O}_1^n\}|\boldsymbol{O}_1^n\right\}\\
\le\ &\frac{\mathrm{var}\{h_{\boldsymbol{\eta}_n}(\boldsymbol{O})|\boldsymbol{O}_1^n\}}{n\left[\frac{\epsilon}{2}(\alpha+\beta)+\frac{\epsilon}{2}\mathbb{E}\{h_{\boldsymbol{\eta}_n}(\boldsymbol{O})|\boldsymbol{O}_1^n\}\right]^2}\\
\le\ &\frac{C_0\mathbb{E}\{h_{\boldsymbol{\eta}_n}(\boldsymbol{O})|\boldsymbol{O}_1^n\}}{n\left[\frac{\epsilon}{2}(\alpha+\beta)+\frac{\epsilon}{2}\mathbb{E}\{h_{\boldsymbol{\eta}_n}(\boldsymbol{O})|\boldsymbol{O}_1^n\}\right]^2}\\
\le\ &\frac{4C_0}{n\epsilon^2}\cdot\frac{1}{4(\alpha+\beta)}=\frac{C_0}{n\epsilon^2(\alpha+\beta)}.
\end{aligned}
$$

Then, for $n>\frac{8C_0}{\epsilon^2(\alpha+\beta)}$,

$$
P\left\{\mathbb{E}\{h_{\boldsymbol{\eta}_n}(\boldsymbol{O})|\boldsymbol{O}_1^n\}-\frac{1}{n}\sum_{i=1}^n h_{\boldsymbol{\eta}_n}(\boldsymbol{O}_i')\le\frac{\epsilon}{2}(\alpha+\beta)+\frac{\epsilon}{2}\mathbb{E}\{h_{\boldsymbol{\eta}_n}(\boldsymbol{O})|\boldsymbol{O}_1^n\}\Big|\boldsymbol{O}_1^n\right\}\ge\frac{7}{8}.
$$

Hence,

$$
\begin{aligned}
&P\left\{\exists\boldsymbol{\eta}\in\Theta:\frac{1}{n}\sum_{i=1}^n h_{\boldsymbol{\eta}}(\boldsymbol{O}_i')-\frac{1}{n}\sum_{i=1}^n h_{\boldsymbol{\eta}}(\boldsymbol{O}_i)\ge\frac{\epsilon}{2}(\alpha+\beta)+\frac{\epsilon}{2}\mathbb{E}\{h_{\boldsymbol{\eta}}(\boldsymbol{O})\}\right\}\\
\ge\ &P\left\{\frac{1}{n}\sum_{i=1}^n h_{\boldsymbol{\eta}_n}(\boldsymbol{O}_i')-\frac{1}{n}\sum_{i=1}^n h_{\boldsymbol{\eta}_n}(\boldsymbol{O}_i)\ge\frac{\epsilon}{2}(\alpha+\beta)+\frac{\epsilon}{2}\mathbb{E}\{h_{\boldsymbol{\eta}_n}(\boldsymbol{O})|\boldsymbol{O}_1^n\}\right\}\\
\ge\ &P\left\{\mathbb{E}\{h_{\boldsymbol{\eta}_n}(\boldsymbol{O})|\boldsymbol{O}_1^n\}-\frac{1}{n}\sum_{i=1}^n h_{\boldsymbol{\eta}_n}(\boldsymbol{O}_i)\ge\epsilon(\alpha+\beta)+\epsilon\mathbb{E}\{h_{\boldsymbol{\eta}_n}(\boldsymbol{O})|\boldsymbol{O}_1^n\},\right.\\
&\qquad\left.\mathbb{E}\{h_{\boldsymbol{\eta}_n}(\boldsymbol{O})|\boldsymbol{O}_1^n\}-\frac{1}{n}\sum_{i=1}^n h_{\boldsymbol{\eta}_n}(\boldsymbol{O}_i')\le\frac{\epsilon}{2}(\alpha+\beta)+\frac{\epsilon}{2}\mathbb{E}\{h_{\boldsymbol{\eta}_n}(\boldsymbol{O})|\boldsymbol{O}_1^n\}\right\}\\
=\ &\mathbb{E}_{\boldsymbol{S}}\Bigg[I_{\left\{\mathbb{E}\{h_{\boldsymbol{\eta}_n}(\boldsymbol{O})|\boldsymbol{O}_1^n\}-\frac{1}{n}\sum_{i=1}^n h_{\boldsymbol{\eta}_n}(\boldsymbol{O}_i)\ge\epsilon(\alpha+\beta)+\epsilon\mathbb{E}\{h_{\boldsymbol{\eta}_n}(\boldsymbol{O})|\boldsymbol{O}_1^n\}\right\}}\\
&\qquad\times\mathbb{E}_{\boldsymbol{S}'}\left\{I_{\left\{\mathbb{E}\{h_{\boldsymbol{\eta}_n}(\boldsymbol{O})|\boldsymbol{O}_1^n\}-\frac{1}{n}\sum_{i=1}^n h_{\boldsymbol{\eta}_n}(\boldsymbol{O}_i')\le\frac{\epsilon}{2}(\alpha+\beta)+\frac{\epsilon}{2}\mathbb{E}\{h_{\boldsymbol{\eta}_n}(\boldsymbol{O})|\boldsymbol{O}_1^n\}\right\}}\Big|\boldsymbol{O}_1^n\right\}\Bigg]\\
=\ &\mathbb{E}_{\boldsymbol{S}}\Bigg[I_{\left\{\mathbb{E}\{h_{\boldsymbol{\eta}_n}(\boldsymbol{O})|\boldsymbol{O}_1^n\}-\frac{1}{n}\sum_{i=1}^n h_{\boldsymbol{\eta}_n}(\boldsymbol{O}_i)\ge\epsilon(\alpha+\beta)+\epsilon\mathbb{E}\{h_{\boldsymbol{\eta}_n}(\boldsymbol{O})|\boldsymbol{O}_1^n\}\right\}}\\
&\qquad\times P\left\{\mathbb{E}\{h_{\boldsymbol{\eta}_n}(\boldsymbol{O})|\boldsymbol{O}_1^n\}-\frac{1}{n}\sum_{i=1}^n h_{\boldsymbol{\eta}_n}(\boldsymbol{O}_i')\le\frac{\epsilon}{2}(\alpha+\beta)+\frac{\epsilon}{2}\mathbb{E}\{h_{\boldsymbol{\eta}_n}(\boldsymbol{O})|\boldsymbol{O}_1^n\}\Big|\boldsymbol{O}_1^n\right\}\Bigg]
\end{aligned}
$$

$$\geq \frac{7}{8}P\left\{\mathbb{E}\{h_{\boldsymbol{\eta}_n}(\boldsymbol{O})|\boldsymbol{O}_1^n\} - \frac{1}{n}\sum_{i=1}^n h_{\boldsymbol{\eta}_n}(\boldsymbol{O}_i) \geq \epsilon(\alpha+\beta) + \epsilon\mathbb{E}\{h_{\boldsymbol{\eta}_n}(\boldsymbol{O})|\boldsymbol{O}_1^n\}\right\}$$
$$= \frac{7}{8}P\left\{\exists \boldsymbol{\eta} \in \Theta : \mathbb{E}\{h_{\boldsymbol{\eta}}(\boldsymbol{O})\} - \frac{1}{n}\sum_{i=1}^n h_{\boldsymbol{\eta}}(\boldsymbol{O}_i) \geq \epsilon(\alpha+\beta) + \epsilon\mathbb{E}\{h_{\boldsymbol{\eta}}(\boldsymbol{O})\}\right\}.$$

Thus, we have shown that, for $n > \frac{8C_0}{\epsilon^2(\alpha+\beta)}$,

$$\begin{aligned} &P\left\{\exists \boldsymbol{\eta} \in \Theta : \mathbb{E}\{h_{\boldsymbol{\eta}}(\boldsymbol{O})\} - \frac{1}{n}\sum_{i=1}^n h_{\boldsymbol{\eta}}(\boldsymbol{O}_i) \geq \epsilon(\alpha+\beta) + \epsilon\mathbb{E}\{h_{\boldsymbol{\eta}}(\boldsymbol{O})\}\right\} \\ &\leq \frac{8}{7}P\left\{\exists \boldsymbol{\eta} \in \Theta : \frac{1}{n}\sum_{i=1}^n h_{\boldsymbol{\eta}}(\boldsymbol{O}_i') - \frac{1}{n}\sum_{i=1}^n h_{\boldsymbol{\eta}}(\boldsymbol{O}_i) \geq \frac{\epsilon}{2}(\alpha+\beta) + \frac{\epsilon}{2}\mathbb{E}\{h_{\boldsymbol{\eta}}(\boldsymbol{O})\}\right\}. \end{aligned} \tag{2}$$

Step 2: Replacing the expectation in equation (2) *with an empirical mean calculated from the ghost sample.*

$$\begin{aligned} &P\left\{\exists \boldsymbol{\eta} \in \Theta : \frac{1}{n}\sum_{i=1}^n h_{\boldsymbol{\eta}}(\boldsymbol{O}_i') - \frac{1}{n}\sum_{i=1}^n h_{\boldsymbol{\eta}}(\boldsymbol{O}_i) \geq \frac{\epsilon}{2}(\alpha+\beta) + \frac{\epsilon}{2}\mathbb{E}\{h_{\boldsymbol{\eta}}(\boldsymbol{O})\}\right\} \\ &\leq P\Bigg\{\exists \boldsymbol{\eta} \in \Theta : \frac{1}{n}\sum_{i=1}^n h_{\boldsymbol{\eta}}(\boldsymbol{O}_i') - \frac{1}{n}\sum_{i=1}^n h_{\boldsymbol{\eta}}(\boldsymbol{O}_i) \geq \frac{\epsilon}{2}(\alpha+\beta) + \frac{\epsilon}{2}\mathbb{E}\{h_{\boldsymbol{\eta}}(\boldsymbol{O})\}, \\ &\qquad \frac{1}{n}\sum_{i=1}^n h_{\boldsymbol{\eta}}^2(\boldsymbol{O}_i) - \mathbb{E}\{h_{\boldsymbol{\eta}}^2(\boldsymbol{O})\} \leq \epsilon(\alpha+\beta+\frac{1}{n}\sum_{i=1}^n h_{\boldsymbol{\eta}}^2(\boldsymbol{O}_i) + \mathbb{E}\{h_{\boldsymbol{\eta}}^2(\boldsymbol{O})\}), \\ &\qquad \frac{1}{n}\sum_{i=1}^n h_{\boldsymbol{\eta}}^2(\boldsymbol{O}_i') - \mathbb{E}\{h_{\boldsymbol{\eta}}^2(\boldsymbol{O})\} \leq \epsilon(\alpha+\beta+\frac{1}{n}\sum_{i=1}^n h_{\boldsymbol{\eta}}^2(\boldsymbol{O}_i') + \mathbb{E}\{h_{\boldsymbol{\eta}}^2(\boldsymbol{O})\})\Bigg\} \\ &\quad + 2P\left\{\exists \boldsymbol{\eta} \in \Theta : \frac{\frac{1}{n}\sum_{i=1}^n h_{\boldsymbol{\eta}}^2(\boldsymbol{O}_i) - \mathbb{E}\{h_{\boldsymbol{\eta}}^2(\boldsymbol{O})\}}{\alpha+\beta+\frac{1}{n}\sum_{i=1}^n h_{\boldsymbol{\eta}}^2(\boldsymbol{O}_i) + \mathbb{E}\{h_{\boldsymbol{\eta}}^2(\boldsymbol{O})\}} > \epsilon\right\}. \end{aligned} \tag{3}$$

Application of Theorem 11.6 of Györfi et al. (2002) to the second probability on the right-hand side of (3) yields

$$\begin{aligned} &P\left\{\exists \boldsymbol{\eta} \in \Theta : \frac{\frac{1}{n}\sum_{i=1}^n h_{\boldsymbol{\eta}}^2(\boldsymbol{O}_i) - \mathbb{E}\{h_{\boldsymbol{\eta}}^2(\boldsymbol{O})\}}{\alpha+\beta+\frac{1}{n}\sum_{i=1}^n h_{\boldsymbol{\eta}}^2(\boldsymbol{O}_i) + \mathbb{E}\{h_{\boldsymbol{\eta}}^2(\boldsymbol{O})\}} > \epsilon\right\} \\ &\leq 4\sup_{\boldsymbol{o}_1^n}\mathcal{N}_1\left(\frac{\epsilon(\alpha+\beta)}{5}, \mathcal{H}_\Theta, \boldsymbol{o}_1^n\right)\exp\left(-\frac{3(\alpha+\beta)\epsilon^2 n}{40B^2}\right) \end{aligned}$$

Now we consider the first probability on the right-hand side of (3). The second inequality inside the probability implies

$$(1+\epsilon)\mathbb{E}\{h_{\boldsymbol{\eta}}^2(\boldsymbol{O})\} \geq (1-\epsilon)\frac{1}{n}\sum_{i=1}^n h_{\boldsymbol{\eta}}^2(\boldsymbol{O}_i) - \epsilon(\alpha+\beta),$$

which is equivalent to

$$\frac{1}{2C_0}\mathbb{E}\{h_{\boldsymbol{\eta}}^2(\boldsymbol{O})\} \geq \frac{1-\epsilon}{2C_0(1+\epsilon)}\frac{1}{n}\sum_{i=1}^n h_{\boldsymbol{\eta}}^2(\boldsymbol{O}_i) - \frac{\epsilon(\alpha+\beta)}{2C_0(1+\epsilon)}.$$

We can deal similarly with the third inequality. Using this and the inequality $\mathbb{E}\{h_{\boldsymbol{\eta}}(\boldsymbol{O})\} \geq \frac{\mathbb{E}\{h_{\boldsymbol{\eta}}^2(\boldsymbol{O})\}}{C_0}$, we can bound the first probability on the right-hand side of (3) by

$$P\left\{\exists \boldsymbol{\eta}\in\Theta : \frac{1}{n}\sum_{i=1}^n h_{\boldsymbol{\eta}}(\boldsymbol{O}_i') - \frac{1}{n}\sum_{i=1}^n h_{\boldsymbol{\eta}}(\boldsymbol{O}_i) \geq \frac{\epsilon}{2}(\alpha+\beta) + \frac{\epsilon}{2}\left(\frac{1-\epsilon}{2C_0(1+\epsilon)}\frac{1}{n}\sum_{i=1}^n h_{\boldsymbol{\eta}}^2(\boldsymbol{O}_i) \right.\right.$$
$$\left.\left. - \frac{\epsilon(\alpha+\beta)}{2C_0(1+\epsilon)} + \frac{1-\epsilon}{2C_0(1+\epsilon)}\frac{1}{n}\sum_{i=1}^n h_{\boldsymbol{\eta}}^2(\boldsymbol{O}_i') - \frac{\epsilon(\alpha+\beta)}{2C_0(1+\epsilon)}\right)\right\}.$$

This shows

$$\begin{aligned}
&P\left\{\exists \boldsymbol{\eta}\in\Theta : \frac{1}{n}\sum_{i=1}^n h_{\boldsymbol{\eta}}(\boldsymbol{O}_i') - \frac{1}{n}\sum_{i=1}^n h_{\boldsymbol{\eta}}(\boldsymbol{O}_i) \geq \frac{\epsilon}{2}(\alpha+\beta) + \frac{\epsilon}{2}\mathbb{E}\{h_{\boldsymbol{\eta}}(\boldsymbol{O})\}\right\} \\
&\leq P\left\{\exists \boldsymbol{\eta}\in\Theta : \frac{1}{n}\sum_{i=1}^n h_{\boldsymbol{\eta}}(\boldsymbol{O}_i') - \frac{1}{n}\sum_{i=1}^n h_{\boldsymbol{\eta}}(\boldsymbol{O}_i) \geq \frac{\epsilon}{2}(\alpha+\beta) - \frac{\epsilon^2(\alpha+\beta)}{2C_0(1+\epsilon)} \right. \\
&\qquad\qquad \left. + \frac{\epsilon(1-\epsilon)}{4C_0(1+\epsilon)}\frac{1}{n}\sum_{i=1}^n \left(h_{\boldsymbol{\eta}}^2(\boldsymbol{O}_i) + h_{\boldsymbol{\eta}}^2(\boldsymbol{O}_i')\right)\right\} \qquad (4) \\
&\quad + 8\sup_{\boldsymbol{o}_1^n}\mathcal{N}_1\left(\frac{\epsilon(\alpha+\beta)}{5}, \mathcal{H}_\Theta, \boldsymbol{o}_1^n\right)\exp\left(-\frac{3(\alpha+\beta)\epsilon^2 n}{40B^2}\right)
\end{aligned}$$

Step 3. Introducing additional randomization by incorporating random signs.

Let $G_1,\ldots,G_n$ be independent and uniformly distributed over the set $\{-1,1\}$ and

independent of $\boldsymbol{O}_1^n$ and $\boldsymbol{O}_1^{n'}$. The first term of inequality (4) is equal to

$$P\left\{\exists\boldsymbol{\eta}\in\Theta:\frac{1}{n}\sum_{i=1}^{n}G_i\left(h_{\boldsymbol{\eta}}(\boldsymbol{O}_i')-h_{\boldsymbol{\eta}}(\boldsymbol{O}_i)\right)\geq\frac{\epsilon}{2}(\alpha+\beta)-\frac{\epsilon^2(\alpha+\beta)}{2C_0(1+\epsilon)}\right.$$
$$\left.+\frac{\epsilon(1-\epsilon)}{4C_0(1+\epsilon)}\frac{1}{n}\sum_{i=1}^{n}\left(h_{\boldsymbol{\eta}}^2(\boldsymbol{O}_i)+h_{\boldsymbol{\eta}}^2(\boldsymbol{O}_i')\right)\right\},$$

and then, this is bounded by

$$\begin{aligned}
&P\left\{\exists\boldsymbol{\eta}\in\Theta:\left|\frac{1}{n}\sum_{i=1}^{n}G_ih_{\boldsymbol{\eta}}(\boldsymbol{O}_i')\right|\geq\frac{1}{2}\left(\frac{\epsilon(\alpha+\beta)}{2}-\frac{\epsilon^2(\alpha+\beta)}{2C_0(1+\epsilon)}\right)+\frac{\epsilon(1-\epsilon)}{4C_0(1+\epsilon)}\frac{1}{n}\sum_{i=1}^{n}h_{\boldsymbol{\eta}}^2(\boldsymbol{O}_i')\right\}\\
&+P\left\{\exists\boldsymbol{\eta}\in\Theta:\left|\frac{1}{n}\sum_{i=1}^{n}G_ih_{\boldsymbol{\eta}}(\boldsymbol{O}_i)\right|\geq\frac{1}{2}\left(\frac{\epsilon(\alpha+\beta)}{2}-\frac{\epsilon^2(\alpha+\beta)}{2C_0(1+\epsilon)}\right)+\frac{\epsilon(1-\epsilon)}{4C_0(1+\epsilon)}\frac{1}{n}\sum_{i=1}^{n}h_{\boldsymbol{\eta}}^2(\boldsymbol{O}_i)\right\}\\
&=2P\left\{\exists\boldsymbol{\eta}\in\Theta:\left|\frac{1}{n}\sum_{i=1}^{n}G_ih_{\boldsymbol{\eta}}(\boldsymbol{O}_i)\right|\geq\frac{\epsilon(\alpha+\beta)}{4}-\frac{\epsilon^2(\alpha+\beta)}{4C_0(1+\epsilon)}+\frac{\epsilon(1-\epsilon)}{4C_0(1+\epsilon)}\frac{1}{n}\sum_{i=1}^{n}h_{\boldsymbol{\eta}}^2(\boldsymbol{O}_i)\right\}.
\end{aligned}\tag{5}$$

Step 4. Conditioning and introducing the covering number.

Next we consider the probability on the right-hand side of (5) conditional on $\boldsymbol{O}_1^n=\{\boldsymbol{o}_1,\ldots,\boldsymbol{o}_n\}$ for fixed $\{\boldsymbol{o}_1,\ldots,\boldsymbol{o}_n\}$:

$$P\left\{\exists\boldsymbol{\eta}\in\Theta:\left|\frac{1}{n}\sum_{i=1}^{n}G_ih_{\boldsymbol{\eta}}(\boldsymbol{o}_i)\right|\geq\frac{\epsilon(\alpha+\beta)}{4}-\frac{\epsilon^2(\alpha+\beta)}{4C_0(1+\epsilon)}+\frac{\epsilon(1-\epsilon)}{4C_0(1+\epsilon)}\frac{1}{n}\sum_{i=1}^{n}h_{\boldsymbol{\eta}}^2(\boldsymbol{o}_i)\right\}$$

Let $\delta>0$ and let $\mathcal{H}_\delta$ be an L_1 δ-cover of $\mathcal{H}_\Theta=\{h_{\boldsymbol{\eta}}:\boldsymbol{\eta}\in\Theta\}$ on $\{\boldsymbol{o}_1,\ldots,\boldsymbol{o}_n\}$. Fix $\boldsymbol{\eta}\in\Theta$. Then there exists $h\in\mathcal{H}_\delta$ such that $\frac{1}{n}\sum_{i=1}^{n}|h(\boldsymbol{o}_i)-h_{\boldsymbol{\eta}}(\boldsymbol{o}_i)|<\delta$. Without loss of generality, we can assume $|h|\leq 2B$. This implies

$$\begin{aligned}
\left|\frac{1}{n}\sum_{i=1}^{n}G_ih_{\boldsymbol{\eta}}(\boldsymbol{o}_i)\right|&=\left|\frac{1}{n}\sum_{i=1}^{n}G_ih(\boldsymbol{o}_i)+\frac{1}{n}\sum_{i=1}^{n}G_i\left(h_{\boldsymbol{\eta}}(\boldsymbol{o}_i)-h(\boldsymbol{o}_i)\right)\right|\\
&\leq\left|\frac{1}{n}\sum_{i=1}^{n}G_ih(\boldsymbol{o}_i)\right|+\frac{1}{n}\sum_{i=1}^{n}|h_{\boldsymbol{\eta}}(\boldsymbol{o}_i)-h(\boldsymbol{o}_i)|\\
&<\left|\frac{1}{n}\sum_{i=1}^{n}G_ih(\boldsymbol{o}_i)\right|+\delta,
\end{aligned}$$

and

$$\begin{aligned}\frac{1}{n}\sum_{i=1}^{n} h_{\boldsymbol{\eta}}^2(\boldsymbol{o}_i) &= \frac{1}{n}\sum_{i=1}^{n} h^2(\boldsymbol{o}_i) + \frac{1}{n}\sum_{i=1}^{n}\left(h_{\boldsymbol{\eta}}^2(\boldsymbol{o}_i) - h^2(\boldsymbol{o}_i)\right)\\ &= \frac{1}{n}\sum_{i=1}^{n} h^2(\boldsymbol{o}_i) + \frac{1}{n}\sum_{i=1}^{n}(h_{\boldsymbol{\eta}}(\boldsymbol{o}_i) + h(\boldsymbol{o}_i))(h_{\boldsymbol{\eta}}(\boldsymbol{o}_i) - h(\boldsymbol{o}_i))\\ &\geq \frac{1}{n}\sum_{i=1}^{n} h^2(\boldsymbol{o}_i) - 3B\frac{1}{n}\sum_{i=1}^{n}|h_{\boldsymbol{\eta}}(\boldsymbol{o}_i) - h(\boldsymbol{o}_i)|\\ &> \frac{1}{n}\sum_{i=1}^{n} h^2(\boldsymbol{o}_i) - 3B\delta.\end{aligned}$$

It follows that

$$\begin{aligned}&P\left\{\exists\boldsymbol{\eta}\in\Theta : \Big|\frac{1}{n}\sum_{i=1}^{n} G_i h_{\boldsymbol{\eta}}(\boldsymbol{o}_i)\Big| \geq \frac{\epsilon(\alpha+\beta)}{4} - \frac{\epsilon^2(\alpha+\beta)}{4C_0(1+\epsilon)} + \frac{\epsilon(1-\epsilon)}{4C_0(1+\epsilon)}\frac{1}{n}\sum_{i=1}^{n} h_{\boldsymbol{\eta}}^2(\boldsymbol{o}_i)\right\}\\ &\leq P\left\{\exists h\in\mathcal{H}_\delta : \Big|\frac{1}{n}\sum_{i=1}^{n} G_i h(\boldsymbol{o}_i)\Big| + \delta\right.\\ &\qquad\left.\geq \frac{\epsilon(\alpha+\beta)}{4} - \frac{\epsilon^2(\alpha+\beta)}{4C_0(1+\epsilon)} + \frac{\epsilon(1-\epsilon)}{4C_0(1+\epsilon)}\left(\frac{1}{n}\sum_{i=1}^{n} h^2(\boldsymbol{o}_i) - 3B\delta\right)\right\}\\ &\leq |\mathcal{H}_\delta|\sup_{h\in\mathcal{H}_\delta} P\left\{\Big|\frac{1}{n}\sum_{i=1}^{n} G_i h(\boldsymbol{o}_i)\Big|\right.\\ &\qquad\left.\geq \frac{\epsilon(\alpha+\beta)}{4} - \frac{\epsilon^2(\alpha+\beta)}{4C_0(1+\epsilon)} - \delta - \frac{3\delta B\epsilon(1-\epsilon)}{4C_0(1+\epsilon)} + \frac{\epsilon(1-\epsilon)}{4C_0(1+\epsilon)}\frac{1}{n}\sum_{i=1}^{n} h^2(\boldsymbol{o}_i)\right\}.\end{aligned}$$

Next we set $\delta = \frac{\epsilon\beta}{8}$. This, together with, $C_0 \geq B \geq 1$, and $0 < \epsilon \leq \frac{1}{2}$ implies

$$\frac{\epsilon\beta}{4} - \frac{\epsilon^2\beta}{4C_0(1+\epsilon)} - \delta - \frac{3\delta B\epsilon(1-\epsilon)}{4C_0(1+\epsilon)} = \frac{\epsilon\beta}{4} - \frac{\epsilon^2\beta}{4C_0(1+\epsilon)} - \frac{\epsilon\beta}{8} - \frac{B\beta\epsilon^2(1-\epsilon)}{16C_0(1+\epsilon)} \geq 0.$$

Thus

$$\begin{aligned}&P\left\{\exists\boldsymbol{\eta}\in\Theta : \Big|\frac{1}{n}\sum_{i=1}^{n} G_i h_{\boldsymbol{\eta}}(\boldsymbol{o}_i)\Big| \geq \frac{\epsilon(\alpha+\beta)}{4} - \frac{\epsilon^2(\alpha+\beta)}{4C_0(1+\epsilon)} + \frac{\epsilon(1-\epsilon)}{4C_0(1+\epsilon)}\frac{1}{n}\sum_{i=1}^{n} h_{\boldsymbol{\eta}}^2(\boldsymbol{o}_i)\right\}\\ &\leq \Big|\mathcal{H}_{\frac{\epsilon\beta}{8}}\Big|\sup_{h\in\mathcal{H}_{\frac{\epsilon\beta}{8}}} P\left\{\Big|\frac{1}{n}\sum_{i=1}^{n} G_i h(\boldsymbol{o}_i)\Big| \geq \frac{\epsilon\alpha}{4} - \frac{\epsilon^2\alpha}{4C_0(1+\epsilon)} + \frac{\epsilon(1-\epsilon)}{4C_0(1+\epsilon)}\frac{1}{n}\sum_{i=1}^{n} h_{\boldsymbol{\eta}}^2(\boldsymbol{o}_i)\right\}.\end{aligned}$$

Step 5. Application of Bernstein's inequality.

In this step, we use Bernstein's inequality to bound

$$P\left\{\Big|\frac{1}{n}\sum_{i=1}^{n} G_i h(\boldsymbol{o}_i)\Big| \geq \frac{\epsilon\alpha}{4} - \frac{\epsilon^2\alpha}{4C_0(1+\epsilon)} + \frac{\epsilon(1-\epsilon)}{4C_0(1+\epsilon)}\frac{1}{n}\sum_{i=1}^{n} h_{\boldsymbol{\eta}}^2(\boldsymbol{o}_i)\right\},$$

where $\boldsymbol{o}_1, \ldots, \boldsymbol{o}_n \in \mathbb{R}^{p+r+4}$ are fixed and $h : \mathbb{R}^{p+r+4} \to [-2B, 2B]$. We relate $\frac{1}{n}\sum_{i=1}^{n} h^2(\boldsymbol{o}_i)$ to the variance of $G_i h(\boldsymbol{o}_i)$,

$$\frac{1}{n}\sum_{i=1}^{n} \operatorname{var}(G_i h(\boldsymbol{o}_i)) = \frac{1}{n}\sum_{i=1}^{n} h^2(\boldsymbol{o}_i)\operatorname{var}(G_i) = \frac{1}{n}\sum_{i=1}^{n} h^2(\boldsymbol{o}_i).$$

Thus the probability above is equal to

$$P\left\{\Big|\frac{1}{n}\sum_{i=1}^{n} D_i\Big| \geq A_1 + A_2\sigma^2\right\},$$

where $D_i = G_i h(\boldsymbol{o}_i)$, $\sigma^2 = \frac{1}{n}\sum_{i=1}^{n} \operatorname{var}(G_i h(\boldsymbol{o}_i))$, $A_1 = \frac{\epsilon\alpha}{4} - \frac{\epsilon^2\alpha}{4C_0(1+\epsilon)}$, $A_2 = \frac{\epsilon(1-\epsilon)}{4C_0(1+\epsilon)}$. Note that $D_1, \ldots, D_n$ are independent random variables satisfying $|D_i| \leq |h(\boldsymbol{o}_i)| \leq 2B$ $(i = 1, \ldots, n)$, and that $A_1, A_2 \geq 0$. By Bernstein's inequality, we have

$$\begin{aligned} P\left\{\Big|\frac{1}{n}\sum_{i=1}^{n} D_i\Big| \geq A_1 + A_2\sigma^2\right\} &\leq 2\exp\left(-\frac{n(A_1 + A_2\sigma^2)^2}{2\sigma^2 + (A_1 + A_2\sigma^2)\frac{4B}{3}}\right) \\ &= 2\exp\left(-\frac{3nA_2}{4B} \cdot \frac{\left(\frac{A_1}{A_2} + \sigma^2\right)^2}{\frac{A_1}{A_2} + \left(1 + \frac{3}{2A_2B}\right)\sigma^2}\right). \end{aligned} \tag{6}$$

A direct calculation shows that, for any $a, b, x > 0$,

$$\frac{(a+x)^2}{a+bx} \geq \frac{\left(a + \frac{b-2}{b}\cdot a\right)^2}{a + b\cdot\frac{b-2}{b}\cdot a} = \frac{4a(b-1)}{b^2}.$$

Thus setting $a = \frac{A_1}{A_2}, b = 1 + \frac{3}{2A_2B}, x = \sigma^2$, and using the bound above, we get for (6) that

$$\frac{3nA_2}{4B}\cdot\frac{\left(\frac{A_1}{A_2} + \sigma^2\right)^2}{\frac{A_1}{A_2} + \left(1 + \frac{3}{2A_2B}\right)\sigma^2} \geq \frac{3nA_2}{4B}\cdot\frac{4\cdot\frac{A_1}{A_2}\cdot\frac{3}{2A_2B}}{\left(1 + \frac{3}{2A_2B}\right)^2} = 18n\cdot\frac{A_1A_2}{(2A_2B+3)^2}.$$

Substituting the formulas for A_1 and A_2 and noticing

$$A_1 = \frac{\epsilon\alpha}{4} - \frac{\epsilon^2\alpha}{4C_0(1+\epsilon)} \geq \frac{\epsilon\alpha}{4} - \frac{\epsilon\alpha}{12} = \frac{\epsilon\alpha}{6},$$

we obtain that

$$\begin{aligned}
18n \cdot \frac{A_1 A_2}{(2A_2 B+3)^2} &\geq 18n \cdot \frac{\epsilon\alpha}{6} \cdot \frac{\epsilon(1-\epsilon)}{4C_0(1+\epsilon)} \cdot \frac{1}{\left(\frac{B\epsilon(1-\epsilon)}{2C_0(1+\epsilon)}+3\right)^2} \\
&\geq 18n \cdot \frac{\epsilon^2(1-\epsilon)\alpha}{24C_0(1+\epsilon)} \cdot \frac{1}{\left(\frac{3-2\sqrt{2}}{2}+3\right)^2} \geq \frac{\epsilon^2(1-\epsilon)\alpha n}{13C_0(1+\epsilon)}.
\end{aligned}$$

Plugging the lower bound above into (6), we finally get that

$$P\left\{\Big|\frac{1}{n}\sum_{i=1}^{n} G_i h(\boldsymbol{o}_i)\Big| \geq \frac{\epsilon\alpha}{4} - \frac{\epsilon^2\alpha}{4C_0(1+\epsilon)} + \frac{\epsilon(1-\epsilon)}{4C_0(1+\epsilon)}\frac{1}{n}\sum_{i=1}^{n} h_{\boldsymbol{\eta}}^2(\boldsymbol{o}_i)\right\} \leq 2\exp\left(-\frac{\epsilon^2(1-\epsilon)\alpha n}{13C_0(1+\epsilon)}\right).$$

So it follows that

$$\begin{aligned}
&P\Bigg\{\exists \boldsymbol{\eta} \in \Theta : \frac{1}{n}\sum_{i=1}^{n}\left(h_{\boldsymbol{\eta}}(\boldsymbol{O}_i') - h_{\boldsymbol{\eta}}(\boldsymbol{O}_i)\right) \geq \frac{\epsilon}{2}(\alpha+\beta) - \frac{\epsilon^2(\alpha+\beta)}{2C_0(1+\epsilon)} \\
&\qquad\qquad + \frac{\epsilon(1-\epsilon)}{4C_0(1+\epsilon)}\frac{1}{n}\sum_{i=1}^{n}\left(h_{\boldsymbol{\eta}}^2(\boldsymbol{O}_i) + h_{\boldsymbol{\eta}}^2(\boldsymbol{O}_i')\right)\Bigg\} \\
&\leq 4\sup_{\boldsymbol{o}_1^n} \mathcal{N}_1\left(\frac{\epsilon\beta}{8}, \mathcal{H}_\Theta, \boldsymbol{o}_1^n\right)\exp\left(-\frac{\epsilon^2(1-\epsilon)\alpha n}{13C_0(1+\epsilon)}\right).
\end{aligned}$$

Step 6. Conclusion.

Steps 1, 2, and 5 imply that, for $n > \frac{8C_0}{\epsilon^2(\alpha+\beta)}$,

$$\begin{aligned}
&P\left\{\exists \boldsymbol{\eta} \in \Theta : \mathbb{E}\{h_{\boldsymbol{\eta}}(\boldsymbol{O})\} - \frac{1}{n}\sum_{i=1}^{n} h_{\boldsymbol{\eta}}(\boldsymbol{O}_i) \geq \epsilon\left(\alpha+\beta+\mathbb{E}\{h_{\boldsymbol{\eta}}(\boldsymbol{O})\}\right)\right\} \\
&\leq \frac{8}{7}\Bigg\{4\sup_{\boldsymbol{o}_1^n} \mathcal{N}_1\left(\frac{\epsilon\beta}{8}, \mathcal{H}_\Theta, \boldsymbol{o}_1^n\right)\exp\left(-\frac{\epsilon^2(1-\epsilon)\alpha n}{13C_0(1+\epsilon)}\right) \\
&\qquad + 8\sup_{\boldsymbol{o}_1^n} \mathcal{N}_1\left(\frac{\epsilon(\alpha+\beta)}{5}, \mathcal{H}_\Theta, \boldsymbol{o}_1^n\right)\exp\left(-\frac{3(\alpha+\beta)\epsilon^2 n}{40B^2}\right)\Bigg\} \\
&\leq 14\sup_{\boldsymbol{o}_1^n} \mathcal{N}_1\left(\frac{\epsilon\beta}{8}, \mathcal{H}_\Theta, \boldsymbol{o}_1^n\right)\exp\left(-\frac{\epsilon^2(1-\epsilon)\alpha n}{40B^2C_0(1+\epsilon)}\right).
\end{aligned}$$

For $n \le \frac{8C_0}{\epsilon^2(\alpha+\beta)}$, we have

$$\exp\left(-\frac{\epsilon^2(1-\epsilon)\alpha n}{40B^2C_0(1+\epsilon)}\right) \ge \exp\left(-\frac{\epsilon^2(1-\epsilon)\alpha}{40B^2C_0(1+\epsilon)} \cdot \frac{8C_0}{\epsilon^2(\alpha+\beta)}\right) \ge \exp\left(-\frac{1}{5}\right) \ge \frac{1}{14},$$

and hence the assertion can be readily concluded.

Proof of Theorem 1.

Combining Lemma 1 and the fact that $\widehat{\boldsymbol{\eta}}_n$ is the minimum of $L_n(\boldsymbol{\eta})$, we get

$$\begin{aligned}
\mathbb{E}d^2(\widehat{\boldsymbol{\eta}}_n, \boldsymbol{\eta}_0) &\le \frac{1}{C_1}\mathbb{E}\left\{L(\widehat{\boldsymbol{\eta}}_n) - L(\boldsymbol{\eta}_0)\right\} \\
&= \frac{1}{C_1}\mathbb{E}\left[L(\widehat{\boldsymbol{\eta}}_n) - L(\boldsymbol{\eta}_0) - 2\{L_n(\widehat{\boldsymbol{\eta}}_n) - L(\boldsymbol{\eta}_0)\} + 2\{L_n(\widehat{\boldsymbol{\eta}}_n) - L(\boldsymbol{\eta}_0)\}\right] \\
&= \frac{1}{C_1}\mathbb{E}\left\{L(\boldsymbol{\eta}_0) - 2L_n(\widehat{\boldsymbol{\eta}}_n) + L(\widehat{\boldsymbol{\eta}}_n)\right\} + \frac{2}{C_1}\mathbb{E}\{L_n(\widehat{\boldsymbol{\eta}}_n) - L(\boldsymbol{\eta}_0)\} \\
&\le \frac{1}{C_1}\mathbb{E}\left\{L(\boldsymbol{\eta}_0) - 2L_n(\widehat{\boldsymbol{\eta}}_n) + L(\widehat{\boldsymbol{\eta}}_n)\right\} + \frac{2}{C_1}\mathbb{E}\{L_n(\boldsymbol{\eta}_n) - L(\boldsymbol{\eta}_0)\} \\
&= \frac{1}{C_1}\mathbb{E}\left\{L(\boldsymbol{\eta}_0) - 2L_n(\widehat{\boldsymbol{\eta}}_n) + L(\widehat{\boldsymbol{\eta}}_n)\right\} + \frac{2}{C_1}\{L(\boldsymbol{\eta}_n) - L(\boldsymbol{\eta}_0)\} \\
&\le \frac{1}{C_1}\mathbb{E}\left\{L(\boldsymbol{\eta}_0) - 2L_n(\widehat{\boldsymbol{\eta}}_n) + L(\widehat{\boldsymbol{\eta}}_n)\right\} + \frac{2C_2}{C_1}d^2(\boldsymbol{\eta}_n, \boldsymbol{\eta}_0).
\end{aligned} \tag{7}$$

Note that

$$\mathbb{E}\left\{L(\boldsymbol{\eta}_0) - 2L_n(\widehat{\boldsymbol{\eta}}_n) + L(\widehat{\boldsymbol{\eta}}_n)\right\} = \mathbb{E}_S\left[\mathbb{E}_{\boldsymbol{O}}\left\{h_{\widehat{\boldsymbol{\eta}}_n}(\boldsymbol{O})\right\} - \frac{2}{n}\sum_{i=1}^{n} h_{\widehat{\boldsymbol{\eta}}_n}(\boldsymbol{O}_i)\right]. \tag{8}$$

By Lemma 2 with $\alpha = \beta = \frac{t}{2}$ and $\epsilon = \frac{1}{2}$, we have

$$\begin{aligned}
&P\left\{\mathbb{E}_{\boldsymbol{O}}\left\{h_{\widehat{\boldsymbol{\eta}}_n}(\boldsymbol{O})\right\} - \frac{2}{n}\sum_{i=1}^{n} h_{\widehat{\boldsymbol{\eta}}_n}(\boldsymbol{O}_i) > t\right\} \\
&\le P\left\{\exists \boldsymbol{\eta} \in \Theta : \mathbb{E}_{\boldsymbol{O}}\left\{h_{\boldsymbol{\eta}}(\boldsymbol{O})\right\} - \frac{2}{n}\sum_{i=1}^{n} h_{\boldsymbol{\eta}}(\boldsymbol{O}_i) > t\right\} \\
&\le 14 \sup_{\boldsymbol{o}_1^n} \mathcal{N}_1\left(\frac{t}{32}, \mathcal{H}_\Theta, \boldsymbol{o}_1^n\right) \exp\left(-\frac{nt}{960B^2C_0}\right).
\end{aligned}$$

Then for $b_n > 0$,

$$\begin{aligned}
\mathbb{E}_S\left[\mathbb{E}_{\boldsymbol{O}}\left\{h_{\widehat{\boldsymbol{\eta}}_n}(\boldsymbol{O})\right\} - \frac{2}{n}\sum_{i=1}^{n} h_{\widehat{\boldsymbol{\eta}}_n}(\boldsymbol{O}_i)\right] &\leq b_n + \int_{b_n}^{\infty} P\left\{\mathbb{E}_{\boldsymbol{O}}\left\{h_{\widehat{\boldsymbol{\eta}}_n}(\boldsymbol{O})\right\} - \frac{2}{n}\sum_{i=1}^{n} h_{\widehat{\boldsymbol{\eta}}_n}(\boldsymbol{O}_i) > t\right\} dt \\
&\leq b_n + \int_{b_n}^{\infty} 14 \sup_{\boldsymbol{o}_1^n} \mathcal{N}_1\left(\frac{t}{32}, \mathcal{H}_\Theta, \boldsymbol{o}_1^n\right) \exp\left(-\frac{nt}{960B^2C_0}\right) dt \\
&\leq b_n + 14 \sup_{\boldsymbol{o}_1^n} \mathcal{N}_1\left(\frac{b_n}{32}, \mathcal{H}_\Theta, \boldsymbol{o}_1^n\right) \int_{b_n}^{\infty} \exp\left(-\frac{nt}{960B^2C_0}\right) dt \\
&= b_n + 14 \sup_{\boldsymbol{o}_1^n} \mathcal{N}_1\left(\frac{b_n}{32}, \mathcal{H}_\Theta, \boldsymbol{o}_1^n\right) \cdot \frac{960B^2C_0}{n} \exp\left(-\frac{nb_n}{960B^2C_0}\right).
\end{aligned}$$

We choose $b_n = \frac{960B^2C_0}{n} \log\left(14 \sup_{\boldsymbol{o}_1^n} \mathcal{N}_1\left(\frac{1}{n}, \mathcal{H}_\Theta, \boldsymbol{o}_1^n\right)\right)$. Note that $\frac{b_n}{32} \geq \frac{1}{n}$ and $\mathcal{N}_1\left(\frac{1}{n}, \mathcal{H}_\Theta, \boldsymbol{o}_1^n\right) \geq \mathcal{N}_1\left(\frac{b_n}{32}, \mathcal{H}_\Theta, \boldsymbol{o}_1^n\right)$. Then we get

$$\mathbb{E}_S\left[\mathbb{E}_{\boldsymbol{O}}\left\{h_{\widehat{\boldsymbol{\eta}}_n}(\boldsymbol{O})\right\} - \frac{2}{n}\sum_{i=1}^{n} h_{\widehat{\boldsymbol{\eta}}_n}(\boldsymbol{O}_i)\right] \leq \frac{960B^2C_0\left\{\log\left(14 \sup_{\boldsymbol{o}_1^n} \mathcal{N}_1\left(\frac{1}{n}, \mathcal{H}_\Theta, \boldsymbol{o}_1^n\right)\right) + 1\right\}}{n}. \tag{9}$$

Therefore, combining (7), (8) and (9), we have

$$\mathbb{E}d^2(\widehat{\boldsymbol{\eta}}_n, \boldsymbol{\eta}_0) \leq \frac{960B^2C_0\left\{\log\left(14 \sup_{\boldsymbol{o}_1^n} \mathcal{N}_1\left(\frac{1}{n}, \mathcal{H}_\Theta, \boldsymbol{o}_1^n\right)\right) + 1\right\}}{nC_1} + \frac{2C_2}{C_1} d^2(\boldsymbol{\eta}_n, \boldsymbol{\eta}_0). \tag{10}$$

Next, we construct an L_1 δ-cover of $\mathcal{H}_\Theta = \{h_{\boldsymbol{\eta}} : \boldsymbol{\eta} \in \Theta\}$ on $\boldsymbol{o}_1, \ldots, \boldsymbol{o}_n$. Let $\boldsymbol{\eta}_1, \ldots, \boldsymbol{\eta}_\ell$, $\ell = \mathcal{N}_1\left(\frac{\delta}{C_3}, \Theta, \boldsymbol{o}_1^n\right)$ be an $\frac{\delta}{C_3}$-cover of Θ on $\boldsymbol{o}_1^n$ with $C_3 > 0$. Let $\boldsymbol{\eta} \in \Theta$ be arbitrary. Then there exists an $\boldsymbol{\eta}_j = (\boldsymbol{\beta}_{j_1}, \boldsymbol{\gamma}_{j_2}, g_{1j_3}, g_{2j_4}, \phi_{j_5})$ such that

$$\begin{aligned}
\|\boldsymbol{\eta} - \boldsymbol{\eta}_j\|_f := \{&\|\boldsymbol{\beta} - \boldsymbol{\beta}_{j_1}\| + \|\boldsymbol{\gamma} - \boldsymbol{\gamma}_{j_2}\| \\
&+\frac{1}{n}\sum_{i=1}^{n} \|g_1(\boldsymbol{x}_i) - g_{1j_3}(\boldsymbol{x}_i)\| + \frac{1}{n}\sum_{i=1}^{n} \|g_2(\boldsymbol{x}_i) - g_{2j_4}(\boldsymbol{x}_i)\| \\
&+\frac{1}{n}\sum_{i=1}^{n} \left\|\phi\left(u_i \exp\{\boldsymbol{\beta}_0^\top \boldsymbol{z}_i + g_{10}(\boldsymbol{x}_i)\}\right) - \phi_{j_5}\left(u_i \exp\{\boldsymbol{\beta}_0^\top \boldsymbol{z}_i + g_{10}(\boldsymbol{x}_i)\}\right)\right\| \\
&+\frac{1}{n}\sum_{i=1}^{n} \left\|\phi\left(v_i \exp\{\boldsymbol{\beta}_0^\top \boldsymbol{z}_i + g_{10}(\boldsymbol{x}_i)\}\right) - \phi_{j_5}\left(v_i \exp\{\boldsymbol{\beta}_0^\top \boldsymbol{z}_i + g_{10}(\boldsymbol{x}_i)\}\right)\right\|\Big\} \\
\leq \frac{\delta}{C_3}.&
\end{aligned}$$

We obtain $\frac{1}{n}\sum_{i=1}^{n}|h_{\boldsymbol{\eta}}(\boldsymbol{o}_i) - h_{\boldsymbol{\eta}_j}(\boldsymbol{o}_i)| \le C_3\|\boldsymbol{\eta} - \boldsymbol{\eta}_j\|_f \le \delta$. Thus $h_{\boldsymbol{\eta}_1}, \ldots, h_{\boldsymbol{\eta}_\ell}$ can be an δ-cover of $\mathcal{H}_\Theta = \{h_{\boldsymbol{\eta}} : \boldsymbol{\eta} \in \Theta\}$ on $\boldsymbol{o}_1^n$ of size $\mathcal{N}_1\left(\frac{\delta}{C_3}, \Theta, \boldsymbol{o}_1^n\right)$. Without loss of generality, we assume $\|\boldsymbol{\beta} - \boldsymbol{\beta}_{j_1}\| < \frac{\delta}{6C_3}$, $\|\boldsymbol{\gamma} - \boldsymbol{\gamma}_{j_2}\| < \frac{\delta}{6C_3}$, and

$$\frac{1}{n}\sum_{i=1}^{n}\|g_1(\boldsymbol{x}_i) - g_{1j_3}(\boldsymbol{x}_i)\| < \frac{\delta}{6C_3},\ \frac{1}{n}\sum_{i=1}^{n}\|g_2(\boldsymbol{x}_i) - g_{2j_4}(\boldsymbol{x}_i)\| < \frac{\delta}{6C_3},$$
$$\frac{1}{n}\sum_{i=1}^{n}\left\|\phi\left(u_i\exp\{\boldsymbol{\beta}_0^\top\boldsymbol{z}_i + g_{10}(\boldsymbol{x}_i)\}\right) - \phi_{j_5}\left(u_i\exp\{\boldsymbol{\beta}_0^\top\boldsymbol{z}_i + g_{10}(\boldsymbol{x}_i)\}\right)\right\| < \frac{\delta}{6C_3},$$
$$\frac{1}{n}\sum_{i=1}^{n}\left\|\phi\left(v_i\exp\{\boldsymbol{\beta}_0^\top\boldsymbol{z}_i + g_{10}(\boldsymbol{x}_i)\}\right) - \phi_{j_5}\left(v_i\exp\{\boldsymbol{\beta}_0^\top\boldsymbol{z}_i + g_{10}(\boldsymbol{x}_i)\}\right)\right\| < \frac{\delta}{6C_3}.$$

Then

$$\mathcal{N}_1\left(\frac{\delta}{C_3}, \Theta, \boldsymbol{o}_1^n\right) \le \mathcal{N}_2\left(\frac{\delta}{6C_3}, \mathcal{B}_1\right)\cdot\mathcal{N}_2\left(\frac{\delta}{6C_3}, \mathcal{B}_2\right)\cdot\mathcal{N}_2^2\left(\frac{\delta}{6C_3}, \mathcal{M}_n\right)\cdot\mathcal{N}_2^2\left(\frac{\delta}{6C_3}, \mathcal{G}_n, \boldsymbol{x}_1^n\right).$$

It is known that both $\mathcal{B}_1$ and $\mathcal{B}_2$ can be covered by $C_4\left(\frac{6C_3}{\delta}\right)^p$ balls with radius $\frac{\delta}{6C_3}$. Based on the bracketing number calculations developed by Shen and Wong (1994), we have

$$\mathcal{N}_2\left(\frac{\delta}{6C_3}, \mathcal{M}_n\right) \le C_5\left(\frac{6C_3}{\delta}\right)^{q_n}.$$

Let $\mathrm{Pdim}(\mathcal{G}_n)$ denote the pseudo dimension of $\mathcal{G}_n$. According to Theorem 12.2 in Anthony and Bartlett (1999), for $n > \mathrm{Pdim}(\mathcal{G}_n)$,

$$\mathcal{N}_2\left(\frac{\delta}{6C_3}, \mathcal{G}_n, \boldsymbol{x}_1^n\right) \le \mathcal{N}_\infty\left(\frac{\delta}{6C_3}, \mathcal{G}_n, \boldsymbol{x}_1^n\right) \le \left(\frac{6C_3 \mathrm{e}\mathcal{B}n}{\delta\mathrm{Pdim}(\mathcal{G}_n)}\right)^{\mathrm{Pdim}(\mathcal{G}_n)}.$$

In addition, by Theorems 3 and 7 in Bartlett et al. (2019), there exist universal constants c_1 and c_2 such that

$$c_1 \cdot \mathcal{SL}\log(\mathcal{S}/\mathcal{L}) \le \mathrm{Pdim}(\mathcal{G}_n) \le c_2 \cdot \mathcal{SL}\log(\mathcal{S}).$$

Let $\delta = \frac{1}{n}$. Then we get

$$\log\left\{\sup_{\boldsymbol{o}_1}\mathcal{N}_1\left(\frac{1}{n}, \mathcal{H}_\Theta, \boldsymbol{o}_1^n\right)\right\} \le C_4\{p + q_n + \mathcal{SL}\log(\mathcal{S})\}\log n. \tag{11}$$

Combining (10) and (11), we obtain

$$\mathbb{E}d^2(\widehat{\boldsymbol{\eta}}_n, \boldsymbol{\eta}_0) \leq C_5 \left\{ \frac{B^2 C_0 \{p + q_n + \mathcal{S}\mathcal{L}\log(\mathcal{S})\} \log n}{n} + d^2(\boldsymbol{\eta}_n, \boldsymbol{\eta}_0) \right\}. \tag{12}$$

Hence, we have

$$d^2(\boldsymbol{\eta}_n, \boldsymbol{\eta}_0) = \|g_{1n} - g_{10}\|^2_{L^2([0,1]^r)} + \|g_{2n} - g_{20}\|^2_{L^2([0,1]^r)} + \|\phi_n - \phi_0\|^2_{\Phi}.$$

By Lemma 1 in Lu et al. (2007), we get $\|\phi_n - \phi_0\|_\Phi = O(n^{-\mu\nu})$, for $1/(2\mu+1) < \nu < 1/(2\mu)$ with $\mu \geq 1$. By Corollary 3.1 in Jiao et al. (2023), it follows that

$$\|g_{1n} - g_{10}\|_{L^2([0,1]^r)} \leq \|g_{1n} - g_{10}\|_\infty \leq 19B_0(\lfloor\xi\rfloor + 1)^2 r^{\lfloor\xi\rfloor + (\xi \vee 1)/2}(NM)^{-2\xi/r},$$

and

$$\|g_{2n} - g_{20}\|_{L^2([0,1]^r)} \leq \|g_{2n} - g_{10}\|_\infty \leq 19B_0(\lfloor\xi\rfloor + 1)^2 r^{\lfloor\xi\rfloor + (\xi \vee 1)/2}(NM)^{-2\xi/r}.$$

Combining these approximation results above and (12), we conclude that

$$\begin{aligned}\mathbb{E}d^2(\widehat{\boldsymbol{\eta}}_n, \boldsymbol{\eta}_0) \leq C\Big\{ & \frac{B^2 C_0 \{p + q_n + \mathcal{S}\mathcal{L}\log(\mathcal{S})\} \log n}{n} + n^{-2\mu\nu} \\ & + B_0^2(\lfloor\xi\rfloor + 1)^4 r^{2\lfloor\xi\rfloor + \xi\vee 1}(NM)^{-4\xi/r}\Big\},\end{aligned}$$

where $C > 0$ is a constant independent of $n, p, r, q_n, \mu, \nu, \xi, B, B_0, C_0, \mathcal{S}, \mathcal{L}, N$ or M.
Proof of Theorem 2.

The efficient score function (Kosorok (2008), Chapter 3) for $\boldsymbol{\theta}_0$ is

$$\ell^*_{\boldsymbol{\theta}}(\boldsymbol{\eta}_0) := \dot{\ell}_{\boldsymbol{\theta}}(\boldsymbol{\eta}_0) - \Pi_{g_{10},g_{20},\phi_0}(\dot{\ell}_{\boldsymbol{\theta}}(\boldsymbol{\eta}_0)|\dot{\boldsymbol{P}}_1 + \dot{\boldsymbol{P}}_2 + \dot{\boldsymbol{P}}_3),$$

where $\dot{\ell}_{\boldsymbol{\theta}}(\boldsymbol{\eta}_0) = (\dot{\ell}_{\boldsymbol{\beta}}(\boldsymbol{\eta}_0)^\top, \dot{\ell}_{\boldsymbol{\gamma}}(\boldsymbol{\eta}_0)^\top)^\top$, and $\Pi_{g_{10},g_{20},\phi_0}(\dot{\ell}_{\boldsymbol{\theta}}(\boldsymbol{\eta}_0)|\dot{\boldsymbol{P}}_1+\dot{\boldsymbol{P}}_2+\dot{\boldsymbol{P}}_3)$ is the projection of $\dot{\ell}_{\boldsymbol{\theta}}(\boldsymbol{\eta}_0)$ onto the sumspace $\dot{\boldsymbol{P}}_1 + \dot{\boldsymbol{P}}_2 + \dot{\boldsymbol{P}}_3$ with $\dot{\boldsymbol{P}}_1 := \{\dot{\ell}_{g_1}(\boldsymbol{\eta}_0)[a] : a \in \overline{\mathbb{S}}_{g_0}\}$, $\dot{\boldsymbol{P}}_2 := \{\dot{\ell}_{g_2}(\boldsymbol{\eta}_0)[b] : b \in \overline{\mathbb{S}}_{g_0}\}$ and $\dot{\boldsymbol{P}}_3 := \{\dot{\ell}_{\phi}(\boldsymbol{\eta}_0)[c] : c \in \overline{\mathbb{S}}_{\phi_0}\}$. Finding $\Pi_{g_{10},g_{20},\phi_0}(\dot{\ell}_{\boldsymbol{\theta}}(\boldsymbol{\eta}_0)|\dot{\boldsymbol{P}}_1+\dot{\boldsymbol{P}}_2+\dot{\boldsymbol{P}}_3)$ is equivalent to finding the least favorable direction $(\boldsymbol{a}^{*\top}, \boldsymbol{b}^{*\top}, \boldsymbol{c}^{*\top})^\top \in \overline{\mathbb{S}}^{2p}_{g_0} \times \overline{\mathbb{S}}^{2p}_{g_0} \times \overline{\mathbb{S}}^{2p}_{\phi_0}$

such that

$$\mathbb{E}\left\{\left(\dot{\ell}_{\boldsymbol{\theta}}(\boldsymbol{\eta}_0)-\dot{\ell}_{g_1}(\boldsymbol{\eta}_0)[\boldsymbol{a}^*]-\dot{\ell}_{g_2}(\boldsymbol{\eta}_0)[\boldsymbol{b}^*]-\dot{\ell}_{\phi}(\boldsymbol{\eta}_0)[\boldsymbol{c}^*]\right)\dot{\ell}_{g_1}(\boldsymbol{\eta}_0)[a]\right\}=0,\ \text{for all } a\in\overline{\mathbb{S}}_{g_0},$$
$$\mathbb{E}\left\{\left(\dot{\ell}_{\boldsymbol{\theta}}(\boldsymbol{\eta}_0)-\dot{\ell}_{g_1}(\boldsymbol{\eta}_0)[\boldsymbol{a}^*]-\dot{\ell}_{g_2}(\boldsymbol{\eta}_0)[\boldsymbol{b}^*]-\dot{\ell}_{\phi}(\boldsymbol{\eta}_0)[\boldsymbol{c}^*]\right)\dot{\ell}_{g_2}(\boldsymbol{\eta}_0)[b]\right\}=0,\ \text{for all } b\in\overline{\mathbb{S}}_{g_0},$$
$$\mathbb{E}\left\{\left(\dot{\ell}_{\boldsymbol{\theta}}(\boldsymbol{\eta}_0)-\dot{\ell}_{g_1}(\boldsymbol{\eta}_0)[\boldsymbol{a}^*]-\dot{\ell}_{g_2}(\boldsymbol{\eta}_0)[\boldsymbol{b}^*]-\dot{\ell}_{\phi}(\boldsymbol{\eta}_0)[\boldsymbol{c}^*]\right)\dot{\ell}_{\phi}(\boldsymbol{\eta}_0)[c]\right\}=0,\ \text{for all } c\in\overline{\mathbb{S}}_{\phi_0}.$$

Then $\Pi_{g_{10},g_{20},\phi_0}(\dot{\ell}_{\boldsymbol{\theta}}(\boldsymbol{\eta}_0)|\dot{\boldsymbol{P}}_1+\dot{\boldsymbol{P}}_2+\dot{\boldsymbol{P}}_3)=\dot{\ell}_{g_1}(\boldsymbol{\eta}_0)[\boldsymbol{a}^*]+\dot{\ell}_{g_2}(\boldsymbol{\eta}_0)[\boldsymbol{b}^*]+\dot{\ell}_{\phi}(\boldsymbol{\eta}_0)[\boldsymbol{c}^*]$. This implies that $(\boldsymbol{a}^{*\top},\boldsymbol{b}^{*\top},\boldsymbol{c}^{*\top})^\top$ minimizes $\mathbb{E}\|\dot{\ell}_\theta(\boldsymbol{\eta})-\dot{\ell}_{g_1}(\boldsymbol{\eta})[\boldsymbol{a}]-\dot{\ell}_{g_2}(\boldsymbol{\eta})[\boldsymbol{b}]-\dot{\ell}_{\phi}(\boldsymbol{\eta})[\boldsymbol{c}]\|_c^2$. By Conditions (C1)-(C7) and Appendix A.4 in Bickel et al. (1993), we know that the minimizer $(\boldsymbol{a}^{*\top},\boldsymbol{b}^{*\top},\boldsymbol{c}^{*\top})^\top$ is well defined and unique. Therefore, the efficient score is

$$\ell^*_{\boldsymbol{\theta}}(\boldsymbol{\eta}_0)=\dot{\ell}_{\boldsymbol{\theta}}(\boldsymbol{\eta}_0)-\dot{\ell}_{g_1}(\boldsymbol{\eta}_0)[\boldsymbol{a}^*]-\dot{\ell}_{g_2}(\boldsymbol{\eta}_0)[\boldsymbol{b}^*]-\dot{\ell}_{\phi}(\boldsymbol{\eta}_0)[\boldsymbol{c}^*],$$

and the information matrix is

$$\boldsymbol{I}(\boldsymbol{\beta}_0)=\mathbb{E}\{\ell^*_{\boldsymbol{\theta}}(\boldsymbol{\eta}_0)^{\otimes 2}\}=\mathbb{E}\left\{\dot{\ell}_{\boldsymbol{\theta}}(\boldsymbol{\eta}_0)-\dot{\ell}_{g_1}(\boldsymbol{\eta}_0)[\boldsymbol{a}^*]-\dot{\ell}_{g_2}(\boldsymbol{\eta}_0)[\boldsymbol{b}^*]-\dot{\ell}_{\phi}(\boldsymbol{\eta}_0)[\boldsymbol{c}^*]\right\}^{\otimes 2}.$$

Lemma 3. *Assume that*

(1) $\mathbb{P}_n\left\{\dot{\ell}_{\boldsymbol{\theta}}(\widehat{\boldsymbol{\eta}}_n)\right\}=\mathbb{P}_n\left\{\dot{\ell}_{g_1}(\widehat{\boldsymbol{\eta}}_n)\left[\boldsymbol{a}^*\right]\right\}=\mathbb{P}_n\left\{\dot{\ell}_{g_2}(\widehat{\boldsymbol{\eta}}_n)\left[\boldsymbol{b}^*\right]\right\}=\mathbb{P}_n\left\{\dot{\ell}_{\phi}(\widehat{\boldsymbol{\eta}}_n)\left[\boldsymbol{c}^*\right]\right\}=o_p\left(n^{-1/2}\right)$,

(2) $\left(\mathbb{P}_n-P\right)\left\{\ell^*_{\boldsymbol{\theta}}(\widehat{\boldsymbol{\eta}}_n)-\ell^*_{\boldsymbol{\theta}}(\boldsymbol{\eta}_0)\right\}=o_p\left(n^{-1/2}\right)$,

(3) $P\left\{\ell^*_{\boldsymbol{\theta}}(\widehat{\boldsymbol{\eta}}_n)-\ell^*_{\boldsymbol{\theta}}(\boldsymbol{\eta}_0)\right\}=-\boldsymbol{I}\left(\boldsymbol{\theta}_0\right)\left(\widehat{\boldsymbol{\theta}}_n-\boldsymbol{\theta}_0\right)+o_p\left(n^{-1/2}\right)$ *hold and suppose* $\boldsymbol{I}\left(\boldsymbol{\theta}_0\right)$ *is nonsingular. Then*

$$n^{1/2}\left(\widehat{\boldsymbol{\theta}}_n-\boldsymbol{\theta}_0\right)=n^{1/2}\boldsymbol{I}^{-1}\left(\boldsymbol{\theta}_0\right)\mathbb{P}_n\left\{\ell^*_{\boldsymbol{\theta}}(\boldsymbol{\eta}_0)\right\}+o_p(1)\xrightarrow{d}\mathcal{N}\left(0,\boldsymbol{I}^{-1}\left(\boldsymbol{\theta}_0\right)\right),n\to\infty.$$

Proof of Lemma 3.

Combining conditions (2) and (3), we have $\mathbb{P}_n\{\ell^*_{\boldsymbol{\theta}}(\widehat{\boldsymbol{\eta}}_n)-\ell^*_{\boldsymbol{\theta}}\left(\boldsymbol{\eta}_0\right)\}=-\boldsymbol{I}\left(\boldsymbol{\theta}_0\right)(\widehat{\boldsymbol{\theta}}_n-\boldsymbol{\theta}_0)+o_p\left(\|\widehat{\boldsymbol{\theta}}_n-\boldsymbol{\theta}_0\|\right)+o_p\left(n^{-1/2}\right)$. By condition (1), it follows that

$$\mathbb{P}_n\ell^*_{\boldsymbol{\theta}}\left(\boldsymbol{\eta}_0\right)=\boldsymbol{I}\left(\boldsymbol{\theta}_0\right)(\widehat{\boldsymbol{\theta}}_n-\boldsymbol{\theta}_0)+o_p\left(\|\widehat{\boldsymbol{\theta}}_n-\boldsymbol{\theta}_0\|\right)+o_p\left(n^{-1/2}\right).$$

Because $\boldsymbol{I}\left(\boldsymbol{\theta}_0\right)$ is nonsingular and $o_p\left(\|\widehat{\boldsymbol{\theta}}_n-\boldsymbol{\theta}_0\|\right)=o_p\left(n^{-1/2}\right)$, we have

$$n^{1/2}\left(\widehat{\boldsymbol{\theta}}_n-\boldsymbol{\theta}_0\right)=n^{1/2}\boldsymbol{I}^{-1}\left(\boldsymbol{\theta}_0\right)\mathbb{P}_n\left\{\ell^*_{\boldsymbol{\theta}}(\boldsymbol{\eta}_0)\right\}+o_p(1)\xrightarrow{d}\mathcal{N}\left(0,\boldsymbol{I}^{-1}\left(\boldsymbol{\theta}_0\right)\right),n\to\infty.$$

Proof of Theorem 3.

Now we verify the conditions in Lemma 3 to show the asymptotic normality of $\widehat{\boldsymbol{\theta}}_n$. By the definition of $\widehat{\boldsymbol{\eta}}_n$, $\mathbb{P}_n\left\{\dot{\ell}_{\boldsymbol{\theta}}(\widehat{\boldsymbol{\eta}}_n)\right\} = 0$. It is easy to show that there exists a $h_n \in \mathcal{M}_n$ such that $\|h_n - h^*\|_\Phi = O\left(n^{-\mu\nu}\right)$, with $\nu = \xi/(\mu(2\xi + r))$, and $\mathbb{P}_n\left\{\dot{\ell}_\phi(\widehat{\boldsymbol{\eta}}_n)[h_n]\right\} = 0$. Therefore, we can write $\mathbb{P}_n\left\{\dot{\ell}_\phi(\widehat{\boldsymbol{\eta}}_n)[h^*]\right\} = I_{1,n} + I_{2,n}$, where $I_{1,n} = (\mathbb{P}_n - P)\left\{\dot{\ell}_\phi(\widehat{\boldsymbol{\eta}}_n)\left[h^* - h_n\right]\right\}$ and $I_{2,n} = P\left\{\dot{\ell}_\phi(\widehat{\boldsymbol{\eta}}_n)\left[h^* - h_n\right] - \dot{\ell}_\phi(\boldsymbol{\eta}_0)\left[h^* - h_n\right]\right\}$. Let

$$\mathcal{L}_1 = \left\{\dot{\ell}_\phi(\boldsymbol{\eta})\left[h^* - h\right] : \boldsymbol{\eta} \in \mathfrak{B}_1 \times \mathfrak{B}_2 \times \mathcal{G}_n \times \mathcal{G}_n \times \mathcal{M}_n, h \in \mathcal{M}_n \text{ and } \|\mathrm{h}^* - \mathrm{h}\|_\Phi \leq \mathrm{n}^{-\mu\nu}\right\}.$$

By the definition of $\dot{\ell}_\phi(\boldsymbol{\eta})[h]$, we have

$$\left\|\dot{\ell}_\phi(\boldsymbol{\eta})\left[h^* - h\right]\right\|_\infty \leq \frac{2\exp\{pA + \mathcal{B} + \mathcal{C}\}}{1 - \exp\{-pA - \mathcal{B} - \mathcal{C}\}} + \frac{2\exp\{3pA + 3\mathcal{B} + 2\mathcal{C} + \exp\{pA + \mathcal{B} + \mathcal{C}\}\}}{\kappa}.$$

Denote $G = \frac{2\exp\{pA+\mathcal{B}+\mathcal{C}\}}{1-\exp\{-pA-\mathcal{B}-\mathcal{C}\}} + \frac{2\exp\{3pA+3\mathcal{B}+2\mathcal{C}+\exp\{pA+\mathcal{B}+\mathcal{C}\}\}}{\kappa}$. Let $\gamma = O(n^{-\mu\nu})$, then we have $\sup_{f\in\mathcal{L}_1} Pf^2 \leq \gamma^2$. By Lemma 3.4.2 of Van Der Vaart and Wellner (1996), we obtain

$$\mathbb{E}\left\{\sup_{f\in\mathcal{L}_1} \sqrt{n}(\mathbb{P}_n - P)(f)\right\} \leq \tilde{J}_{[]}(\gamma, \mathcal{L}_1, \|\cdot\|_{L_2(P)}) + \frac{\tilde{J}^2_{[]}(\gamma, \mathcal{L}_1, \|\cdot\|_{L_2(P)})}{\gamma^2\sqrt{n}} G, \tag{13}$$

where $\tilde{J}_{[]}(\gamma, \mathcal{L}_1, \|\cdot\|_{L_2(P)}) = \int_0^\gamma \sqrt{1 + \log \mathcal{N}_{[]}(\epsilon, \mathcal{L}_1, \|\cdot\|_{L_2(P)})} d\epsilon$ is the entropy integral. According to Theorem 2.7.11 of Van Der Vaart and Wellner (1996), for any $\epsilon > 0$, we have

$$\begin{aligned}
&\sqrt{1 + \log \mathcal{N}_{[]}(\epsilon, \mathcal{L}_1, \|\cdot\|_{L_2(P)})} \\
\leq\ &\sqrt{1 + \log \mathcal{N}\left(\frac{\epsilon}{2G}, \mathfrak{B}_1 \times \mathfrak{B}_2 \times \mathcal{G}_n \times \mathcal{G}_n \times \mathcal{M}_n \times \mathcal{M}_n, \|\cdot\|_2\right)} \\
\leq\ &\sqrt{1 + \log \mathcal{N}_\infty\left(\frac{\epsilon}{2G}, \mathfrak{B}_1 \times \mathfrak{B}_2 \times \mathcal{G}_n \times \mathcal{G}_n \times \mathcal{M}_n \times \mathcal{M}_n, n\right)} \\
\lesssim\ &\sqrt{1 + \{p + q_n + \mathcal{S}\mathcal{L}\log(\mathcal{S})\}\log\left(\frac{2G\mathcal{B}n}{\epsilon}\right)} \\
\leq\ &\left\{\sqrt{\log\left(\frac{2G\mathcal{B}n}{\epsilon}\right)} + 1\right\}\sqrt{p + q_n + \mathcal{S}\mathcal{L}\log(\mathcal{S})}.
\end{aligned}$$

Then, we have

$$
\begin{aligned}
\tilde{J}_{[]}(\gamma, \mathcal{L}_1, \|\cdot\|_{L_2(P)}) &\lesssim \int_0^\gamma \left\{ \sqrt{\log\left(\frac{\mathcal{B}n}{\epsilon}\right)} + 1 \right\} \sqrt{p + q_n + \mathcal{S}\mathcal{L}\log(\mathcal{S})} d\epsilon \\
&= \sqrt{p + q_n + \mathcal{S}\mathcal{L}\log(\mathcal{S})} \left\{ \gamma + \int_0^\gamma \sqrt{\log\left(\frac{\mathcal{B}n}{\epsilon}\right)} d\epsilon \right\} \\
&\leq \sqrt{p + q_n + \mathcal{S}\mathcal{L}\log(\mathcal{S})} \left\{ \gamma + \int_0^\gamma \left(\sqrt{\log(\mathcal{B}n)} + \sqrt{\log\left(\frac{1}{\epsilon}\right) \vee 0} \right) d\epsilon \right\} \\
&\leq \sqrt{p + q_n + \mathcal{S}\mathcal{L}\log(\mathcal{S})} \gamma \left\{ 1 + \sqrt{\log(\mathcal{B}n)} + \sqrt{\log\left(\frac{1}{\gamma}\right) \vee 1} \right\},
\end{aligned}
$$

where the last inequality can be derived from the fact that

$$
\int_0^x \sqrt{\log(1/t)} \vee 0 \; dt \leq x(\sqrt{\log(1/x)} \vee 1),
$$

and $a \vee b$ represents $\max(a, b)$. We thus obtain

$$
\tilde{J}_{[]}(\gamma, \mathcal{L}_1, \|\cdot\|_{L_2(P)}) \lesssim \gamma \sqrt{\{p + q_n + \mathcal{S}\mathcal{L}\log(\mathcal{S})\} \log n} := V_n. \tag{14}
$$

Based on the assumptions $N \asymp \left(\frac{n}{\log n}\right)^{\frac{r}{2r+4\xi}}$, that M and p are fixed constants independent of n, we obtain

$$
p + q_n + \mathcal{S}\mathcal{L}\log(\mathcal{S}) = O\left(\left(\frac{n}{\log n}\right)^{\frac{r}{2\xi+r}} \right).
$$

According to the assumption $\nu = \xi/(\mu(2\xi + r))$ with $\mu \geq 1$, it follows that $\gamma = O(n^{-\mu\nu}) = O(n^{-\frac{\xi}{2\xi+r}})$. Hence, $V_n = O\left(n^{\frac{r-2\xi}{2(2\xi+r)}} (\log n)^{\frac{\xi}{2\xi+r}}\right)$. Combining (13) and (14), under the assumption $\xi > r/2$, we have

$$
\mathbb{E}\left\{ \sup_{f \in \mathcal{L}_1} \sqrt{n}(\mathbb{P}_n - P)(f) \right\} \lesssim V_n + \frac{V_n^2}{\gamma^2 \sqrt{n}} G = O\left(n^{\frac{r-2\xi}{2(2\xi+r)}} (\log n)^{\frac{2\xi}{2\xi+r}}\right) = o(1).
$$

Therefore, we have $I_{1,n} = o_p(n^{-1/2})$. In addition, the Cauchy-Schwarz inequality yields

$$
\begin{aligned}
I_{2,n} &\leq Cd(\widehat{\boldsymbol{\eta}}_n, \boldsymbol{\eta}_0)\|h^* - h_n\|_\Phi \\
&\leq Cn^{-\mu\nu} d(\widehat{\boldsymbol{\eta}}_n, \boldsymbol{\eta}_0).
\end{aligned}
$$

Hence $I_{2,n} = o_p(n^{-1/2})$. Thus, we have $\mathbb{P}_n \left\{ \dot{\ell}_\phi(\widehat{\boldsymbol{\eta}}_n) \left[c^*\right] \right\} = o_p(n^{-1/2})$. The proofs of $\mathbb{P}_n \left\{ \dot{\ell}_{g_1}(\widehat{\boldsymbol{\eta}}_n) \left[a^*\right] \right\} = o_p\left(n^{-1/2}\right)$ and $\mathbb{P}_n \left\{ \dot{\ell}_{g_2}(\widehat{\boldsymbol{\eta}}_n) \left[b^*\right] \right\} = o_p\left(n^{-1/2}\right)$ are similar. By the definition of $\widehat{\boldsymbol{\eta}}_n$, $\mathbb{P}_n \left\{ \dot{\ell}_{g_1}(\widehat{\boldsymbol{\eta}}_n) \left[a\right] \right\} = 0$ for any $a \in \mathcal{G}_n$. According to Corollary 3.1 in Jiao et al. (2023), there exists $a_n \in \mathcal{G}_n$ such that $\|a^* - a_n\|_{L^2} \leq 19B_0(\lfloor \xi \rfloor + 1)^2 r^{\lfloor \xi \rfloor + (\xi \vee 1)/2}(NM)^{-2\xi/r}$. Therefore, it suffices to show $\mathbb{P}_n \left\{ \dot{\ell}_{g_1}(\widehat{\boldsymbol{\eta}}_n) \left[a^* - a_n\right] \right\} = o_p\left(n^{-1/2}\right)$. The term $\mathbb{P}_n \left\{ \dot{\ell}_{g_1}(\widehat{\boldsymbol{\eta}}_n) \left[a^* - a_n\right] \right\}$ can be written as $I_{3,n} + I_{4,n}$, where

$$I_{3,n} = (\mathbb{P}_n - P)\left\{ \dot{\ell}_{g_1}(\widehat{\boldsymbol{\eta}}_n) \left[a^* - a_n\right] \right\} \text{ and } I_{4,n} = P\Big\{ \dot{\ell}_{g_1}(\widehat{\boldsymbol{\eta}}_n)[a^* - a_n] - \dot{\ell}_{g_1}(\boldsymbol{\eta}_0) \left[a^* - a_n\right] \Big\}.$$

Define

$$\mathcal{L}_2 = \Big\{ \dot{\ell}_{g_1}(\boldsymbol{\eta}) \left[a_j^* - a\right] : \boldsymbol{\eta} \in \mathfrak{B}_1 \times \mathfrak{B}_2 \times \mathcal{G}_n \times \mathcal{G}_n \times \mathcal{M}_n, \\ \|a_j^* - a\|_\infty \leq 19B_0(\lfloor \xi \rfloor + 1)^2 r^{\lfloor \xi \rfloor + (\xi \vee 1)/2}(NM)^{-2\xi/r} \Big\}.$$

The proofs of $I_{3,n} = o_p(n^{-1/2})$ and $I_{4,n} = o_p(n^{-1/2})$ are similar to the proofs of $I_{1,n}$ and $I_{2,n}$. For $0 < \varepsilon < \varsigma$, define $\mathcal{L}_3 = \{\ell^*_{\boldsymbol{\theta}}(\boldsymbol{\eta}) - \ell^*_{\boldsymbol{\theta}}(\boldsymbol{\eta}_0) : \boldsymbol{\eta} \in \mathfrak{B}_1 \times \mathfrak{B}_2 \times \mathcal{G}_n \times \mathcal{G}_n \times \mathcal{M}_n, d(\boldsymbol{\eta}, \boldsymbol{\eta}_0) \leq \varsigma\}$. Using similar proofs as above, we get $(\mathbb{P}_n - P)\{\ell^*_{\boldsymbol{\theta}}(\widehat{\boldsymbol{\eta}}_n) - \ell^*_{\boldsymbol{\theta}}(\boldsymbol{\eta}_0)\} = o_p\left(n^{-1/2}\right)$. By Taylor expansion and the assumptions in Theorem 3, it follows that $P\{\ell^*_{\boldsymbol{\theta}}(\widehat{\boldsymbol{\eta}}_n) - \ell^*_{\boldsymbol{\theta}}(\boldsymbol{\eta}_0)\} = -\boldsymbol{I}(\boldsymbol{\theta}_0)(\widehat{\boldsymbol{\theta}}_n - \boldsymbol{\theta}_0) + o_p\left(n^{-1/2}\right)$.

Web Appendix B: Hyperparameter settings and additional simulation results

This section presents the hyperparameter settings used for model validation and the supplementary results in Section 5, including some additional simulation results.

Table S1 summarizes the hyperparameter settings, including the number of layers {1, 2, 3}, the number of neurons per layer {25, 50, 75, 100}, dropout rate {0.0, 0.01, 0.1}, and learning rate $\{1 \times 10^{-4}, 5 \times 10^{-4}, 1 \times 10^{-3}\}$.

To evaluate the performance of our proposed method, we predict the survival functions of three subjects, respectively, with covariates: $Z = 0$, $\boldsymbol{X} = (0.1, 0.3, 0.5, 0.7)^\top$; $Z = 0$, $\boldsymbol{X} = (0.8, 0.5, 0.2, 0)^\top$; $Z = 1$, $\boldsymbol{X} = (0.8, 0.5, 0.2, 0)^\top$. Figures S1-S3 depict the prediction of survival functions by the DGAHM (dotted line) and EHM (dashed line) methods under two kinds of baseline cumulative hazard functions ($\Lambda_0(t) = t/8$ (the two leftmost columns) and $\Lambda_0(t) = t^{3/4}/6$ (the two rightmost columns)) for the above three

subjects, respectively, along with true values of the survival functions (black curve). The predicted survival curves for the remaining three individuals are shown in Figures S1-S3 in Web Appendix B. All figures demonstrate that the DGAHM estimates are approximately equal to the true survival function. In contrast, the EHM estimates for cases 2-4 exhibit substantial biases. These results suggest that the DGAHM method performs well in all the situations considered.

Table S1: Hyperparameter settings.

Number of layers	1	2	3	
Number of neurons	25	50	75	100
Dropout rate	0.0	0.01	0.1	
Learning rate	1×10^{-4}	5×10^{-4}	1×10^{-3}	

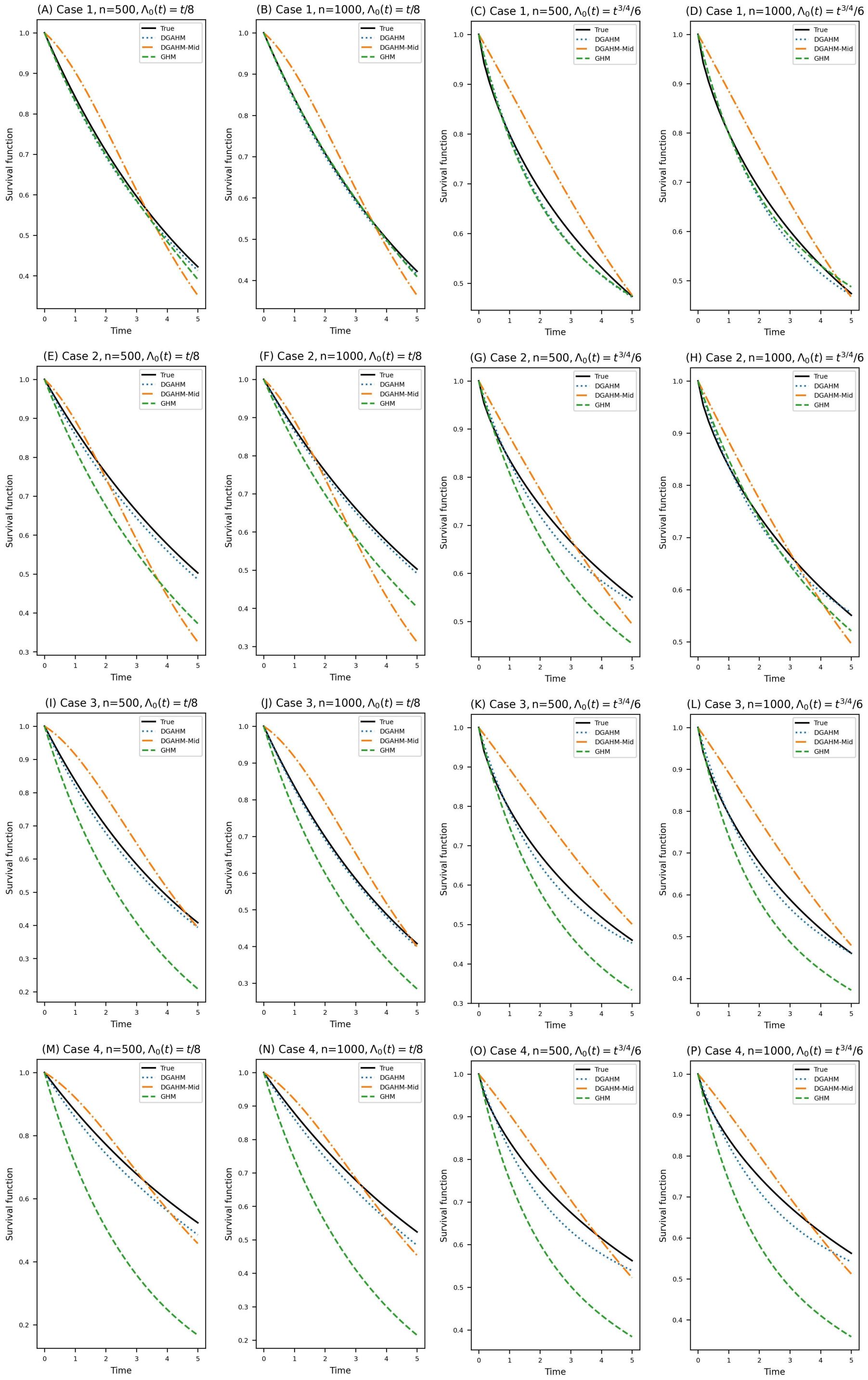

Figure S1: Predictions of survival functions by the DGAHM (dotted line), DGAHM-Mid (dash-dotted line) and EHM (dashed line) methods under two kinds of baseline cumulative hazard functions ($\Lambda_0(t) = t/8$ (the two leftmost columns) and $\Lambda_0(t) = t^{3/4}/6$ (the two rightmost columns)) for the subject with $Z = 0$ and $\boldsymbol{X} = (0.1, 0.3, 0.5, 0.7)^\top$, along with true values of the survival functions (black curve).

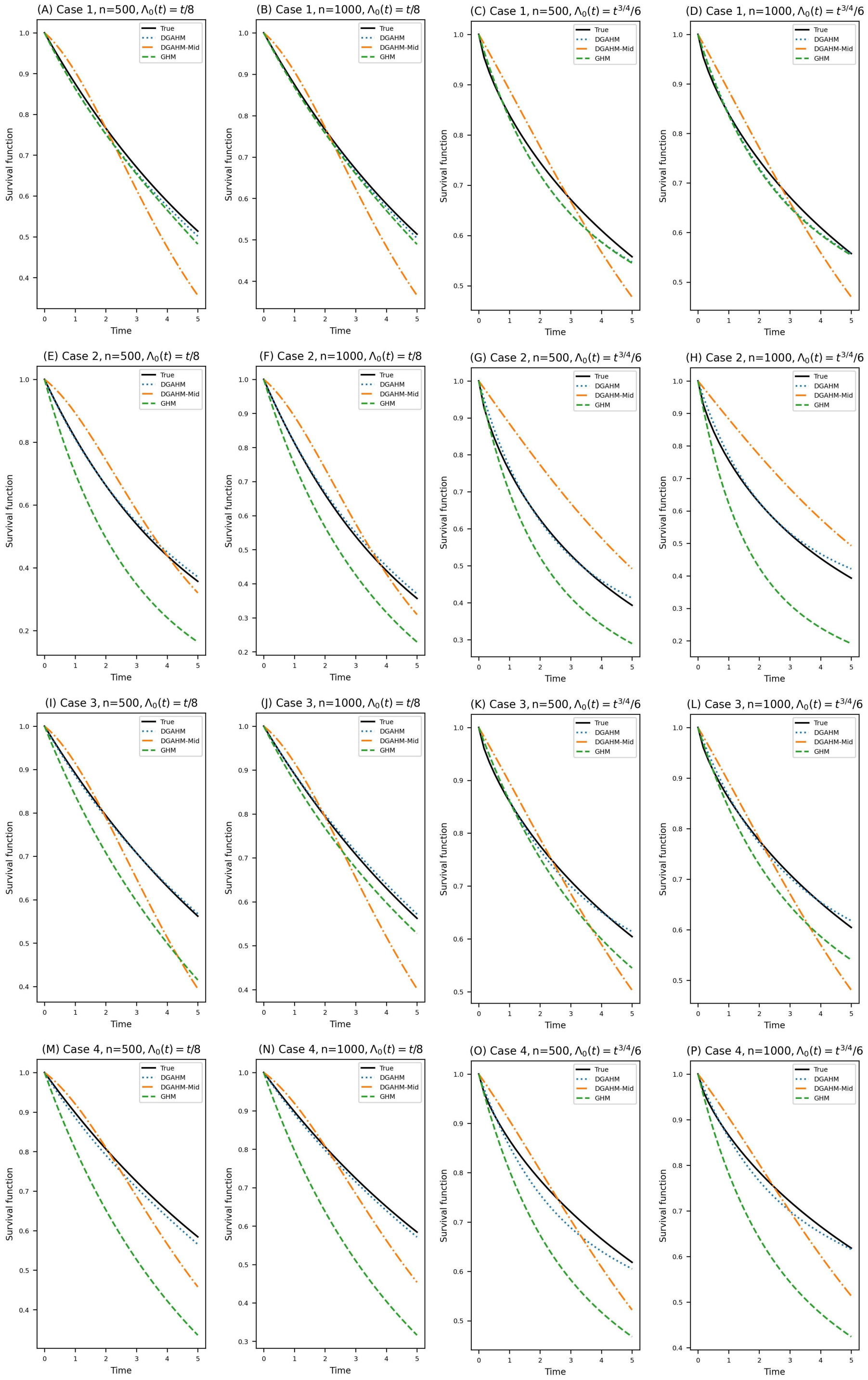

Figure S2: Predictions of survival functions by the DGAHM (dotted line), DGAHM-Mid (dash-dotted line) and EHM (dashed line) methods under two kinds of baseline cumulative hazard functions ($\Lambda_0(t) = t/8$ (the two leftmost columns) and $\Lambda_0(t) = t^{3/4}/6$ (the two rightmost columns)) for the subject with $Z = 0$ and $\boldsymbol{X} = (0.8, 0.5, 0.2, 0)^\top$, along with true values of the survival functions (black curve).

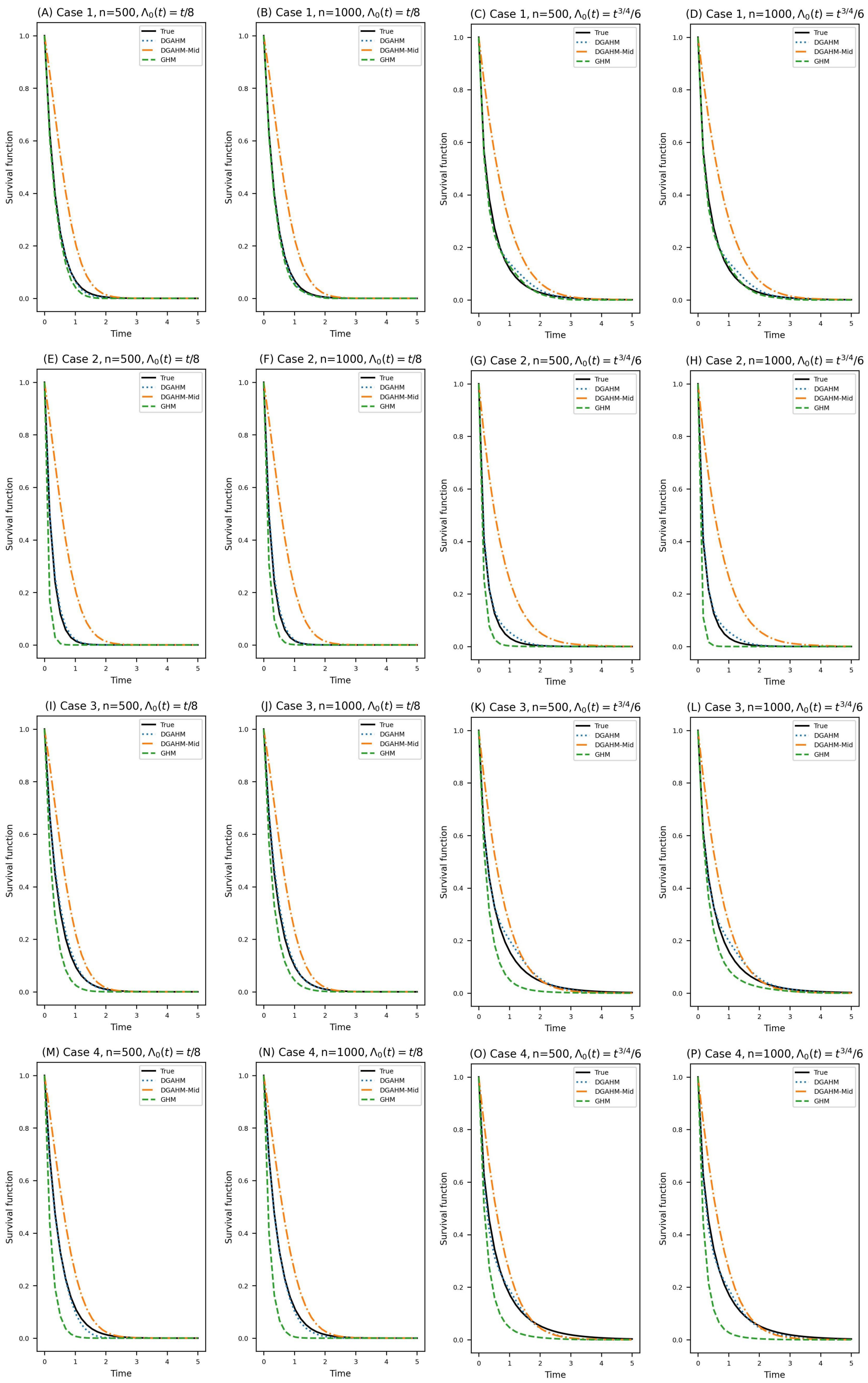

Figure S3: Predictions of survival functions by the DGAHM (dotted line), DGAHM-Mid (dash-dotted line) and EHM (dashed line) methods under two kinds of baseline cumulative hazard functions ($\Lambda_0(t) = t/8$ (the two leftmost columns) and $\Lambda_0(t) = t^{3/4}/6$ (the two rightmost columns)) for the subject with $Z = 1$ and $\boldsymbol{X} = (0.8, 0.5, 0.2, 0)^\top$, along with true values of the survival functions (black curve).

Web Appendix C: Optimization algorithm

To address the specific concerns regarding hyperparameter selection and training stability, we provide the following practical guidelines and algorithmic guarantees:

- *Knot Number:* Theory suggests the number of knots should grow at a rate of $O(n^{\nu})$ with ν set to be a constant between 0 and 1/2 such as 1/3, and practically, one can treat it as a tuning parameter and choose it by, for example, BIC. On the other hand, many authors have pointed out that the analysis results are usually insensitive to q_n and the placement of knots (Lu et al., 2009; Deng et al., 2015).

- *Support Search:* To prevent boundary overflow and eliminate the need for manual support tuning, Algorithm 1 dynamically constructs the knot sequence based on the empirical distribution of the initial risk scores. Specifically, we compute $M^{(0)} = \left\{ V_i \exp\{\widehat{\boldsymbol{\beta}}^{(0)\top} \boldsymbol{Z}_i + \widehat{g}_1^{(0)}(\boldsymbol{X}_i)\} \right\}_{i=1}^{n_{train}}$. Instead of using a fixed support, we place the internal knots at the quantiles of $M^{(0)}$. Crucially, to accommodate potential expansions during subsequent DNN updates and avoid unstable extrapolation, we artificially extend the right boundary by adding extreme knots. The final sequence is defined as

$$\text{Knots} = \left(0, q_1^{(0)}, \ldots, q_{k_n-2}^{(0)}, q_{\max}^{(0)}, 10 \cdot q_{\max}^{(0)}, 100 \cdot q_{\max}^{(0)}\right),$$

  where $q_{\max}^{(0)} = \max(M^{(0)})$, and $q_i^{(0)}$ represents the $\frac{i}{k_n-1}$-th quantile of $M^{(0)}$. This data-driven expansion guarantees numerical stability without adding any tuning burden.

- *Early Stopping & Optimizer:* To ensure stable convergence and prevent overfitting without excessive manual tuning, we employ the Adam optimizer alongside a robust early stopping strategy. We set the maximum number of training epochs to 1000 and monitor the validation loss. If the validation loss fails to improve for 10 consecutive epochs, the training is automatically halted. The DNN parameters from the epoch yielding the lowest validation loss are then restored as the final model.

The detailed procedure of Algorithm 1 is outlined as follows:

Initialization Phase: First, we fix the initial B-spline basis functions constructed over an equally divided support set $[0, 1000]$. Given these fixed bases, the initial values for the linear parameters $\widehat{\boldsymbol{\beta}}^{(0)}$, $\widehat{\boldsymbol{\gamma}}^{(0)}$, and the DNNs $\widehat{g}_1^{(0)}$ and $\widehat{g}_2^{(0)}$ are estimated using

the EHM method. Next, we compute $M^{(0)} = \{V_i \exp\{\widehat{\boldsymbol{\beta}}^{(0)\top} \boldsymbol{Z}_i + \widehat{g}_1^{(0)}(\boldsymbol{X}_i)\}\}_{i=1}^{n_{train}}$. A knot sequence is then defined using the quantiles of $M^{(0)}$, specifically placing interior knots at the $\frac{i}{k_n-1}$-th quantiles, and extending the boundary knots to $10 \cdot q_{\max}^{(0)}$ and $100 \cdot q_{\max}^{(0)}$, where $q_{\max}^{(0)}$ is the maximum value of $M^{(0)}$. With the initial basis functions $\boldsymbol{B}_m^{(0)}$ determined, the initial B-spline coefficients $\widehat{\boldsymbol{c}}^{(0)}$ are obtained by minimizing the negative log-likelihood on the training data while keeping the other parameters fixed.

Alternating Optimization Phase: After initialization, the algorithm proceeds by iteratively alternating between two main steps for a maximum of 50 iterations:

- **Step A (Updating DNN parameters):** We specify a neural network architecture comprising $\mathcal{L}$ hidden layers and $\mathcal{W}$ nodes per layer, incorporating dropout regularization with a probability of d_r. For a maximum of 1000 epochs, the DNN functions g_1 and g_2 are updated by minimizing the training loss using the `Adam` optimizer. To prevent overfitting, an early stopping mechanism is implemented using a separate validation dataset. At each epoch, the negative log-likelihood is evaluated on the validation data. If the validation loss fails to improve for 10 consecutive epochs, the training process is terminated, and the model weights that yielded the lowest validation loss are retained as $\widehat{g}_1^{(k+1)}$ and $\widehat{g}_2^{(k+1)}$.

- **Step B (Updating linear parameters and B-spline coefficients):** Utilizing the newly updated DNN $\widehat{g}_1^{(k+1)}$ and the previous linear parameters $\widehat{\boldsymbol{\beta}}^{(k)}$, we recompute $M^{(k+1)} = \{V_i \exp\{\widehat{\boldsymbol{\beta}}^{(k)\top} \boldsymbol{Z}_i + \widehat{g}_1^{(k+1)}(\boldsymbol{X}_i)\}\}_{i=1}^{n_{train}}$ and update the corresponding B-spline basis functions $\boldsymbol{B}_m^{(k+1)}$ following the same knot construction rule. Subsequently, the updated linear parameters $\widehat{\boldsymbol{\beta}}^{(k+1)}$, $\widehat{\boldsymbol{\gamma}}^{(k+1)}$, and B-spline coefficients $\widehat{\boldsymbol{c}}^{(k+1)}$ are jointly estimated by minimizing the training loss, holding the DNN functions constant.

Convergence Criterion: Steps A and B are repeated iteratively. The alternating optimization process terminates when the Euclidean distances between consecutive estimates of all parametric components are strictly less than a predefined tolerance threshold of 0.01 (i.e., ($\|\widehat{\boldsymbol{\beta}}^{(k+1)} - \widehat{\boldsymbol{\beta}}^{(k)}\| < 0.01$, $\|\widehat{\boldsymbol{\gamma}}^{(k+1)} - \widehat{\boldsymbol{\gamma}}^{(k)}\| < 0.01$, and $\|\widehat{\boldsymbol{c}}^{(k+1)} - \widehat{\boldsymbol{c}}^{(k)}\| < 0.01$), or when the maximum number of iterations is reached.

Algorithm 1: Maximum likelihood estimation via alternating optimization.

Input: Training data $\{(\Delta_{1i}^{train}, \Delta_{2i}^{train}, U_i^{train}, V_i^{train}, \boldsymbol{Z}_i^{train}, \boldsymbol{X}_i^{train})\}_{i=1}^{n_{train}}$ and validation data $\{(\Delta_{1j}^{valid}, \Delta_{2j}^{valid}, U_j^{valid}, V_j^{valid}, \boldsymbol{Z}_j^{valid}, \boldsymbol{X}_j^{valid})\}_{j=1}^{n_{valid}}$; Hidden layer $\mathcal{L}$; Number of node $\mathcal{W}$; Dropout rate $= d_r$; Learning rate η; Number of knots k_n; Spline degree m.

Output: Estimated parameters $\widehat{\boldsymbol{\beta}}, \widehat{\boldsymbol{\gamma}}$; Trained DNNs $\widehat{g}_1, \widehat{g}_2$; B-spline coefficients $\widehat{\boldsymbol{c}}$.

1 **Initialization:** Fixing the initial B-spline basis functions constructed over an equally divided support set $[0, 1000]$, we initialize $\widehat{\boldsymbol{\beta}}^{(0)}$, $\widehat{\boldsymbol{\gamma}}^{(0)}$, $\widehat{g}_1^{(0)}$ and $\widehat{g}_2^{(0)}$ via the EHM method.

2 **Determine B-spline basis functions:** Compute $M^{(0)} = \Big\{V_i \exp\{\widehat{\boldsymbol{\beta}}^{(0)\top}\boldsymbol{Z}_i + \widehat{g}_1^{(0)}(\boldsymbol{X}_i)\}\Big\}_{i=1}^{n_{train}}$; The knot sequence is defined as: $\text{Knots} = \Big(0, q_1^{(0)}, \ldots, q_{k_n-2}^{(0)}, q_{\max}^{(0)}, 10 \cdot q_{\max}^{(0)}, 100 \cdot q_{\max}^{(0)}\Big)$ where $q_{\max}^{(0)} = \max(M^{(0)})$, and $q_i^{(0)}$ represents the $\frac{i}{k_n-1}$-th quantile of $M^{(0)}$. Obtain the basis functions $\boldsymbol{B}_m^{(0)} = (B_1^{(0)}, \ldots, B_{q_n}^{(0)})^\top$ with $q_n = m + k_n + 1$.

3 **Obtain the initialization of B-spline coefficients $\widehat{\boldsymbol{c}}^{(0)}$:** Estimate $\boldsymbol{c}_0$ by optimizing $\widehat{\boldsymbol{c}}^{(0)} = \arg\min_{\boldsymbol{c} \in \mathbb{R}^{q_n}} L_{n_{train}}(\widehat{\boldsymbol{\beta}}^{(0)}, \widehat{\boldsymbol{\gamma}}^{(0)}, \widehat{g}_1^{(0)}, \widehat{g}_2^{(0)}, \boldsymbol{c}^\top \boldsymbol{B}_m^{(0)})$.

4 **for** $k = 1, \ldots, 50$ **do**

5   ***Step A: Update DNN parameters***

6   Construct a neural network with $\mathcal{L}$ inner layers and with a number of nodes $\mathcal{W}$.

7   Set $loss_{best} = \infty$, $patience = 0$. Initialize $result_{loss} \leftarrow \emptyset$, $result_{\widehat{g}_1} \leftarrow \emptyset$, $result_{\widehat{g}_2} \leftarrow \emptyset$.

8   **for** $epoch = 1, 2, \ldots, 1000$ **do**

9   Estimate (g_{10}, g_{20}) by optimizing

$$(\widehat{g}_1^{(k+1)}, \widehat{g}_2^{(k+1)}) = \arg\min_{(g_1, g_2) \in \mathcal{G}_n \times \mathcal{G}_n} L_{n_{train}}(\widehat{\boldsymbol{\beta}}^{(k)}, \widehat{\boldsymbol{\gamma}}^{(k)}, g_1, g_2, \widehat{\boldsymbol{c}}^{(k)\top} \boldsymbol{B}_m^{(k)})$$

        using the training data and the Adam optimizer with learning rate $= \eta$ and dropout rate $= d_r$. Calculate the following negative log-likelihood using the validation data: $s = L_{n_{valid}}(\widehat{\boldsymbol{\beta}}^{(k)}, \widehat{\boldsymbol{\gamma}}^{(k)}, \widehat{g}_1^{(k+1)}, \widehat{g}_2^{(k+1)}, \widehat{\boldsymbol{c}}^{(k)\top} \boldsymbol{B}_m^{(k)})$.

10   **if** $s < loss_{best}$ **then**

11   Update $loss_{best} = s$, $patience = 0$, $\widehat{g}_{best} = \widehat{g}$

12   **end**

13   **else**

14   Update $patience = patience + 1$

15   **end**

16   **if** $patience \geq 10$ **then**

17   Append $L_{n_{train}}(\widehat{\boldsymbol{\beta}}^{(k)}, \widehat{\boldsymbol{\gamma}}^{(k)}, \widehat{g}_1^{(k+1)}, \widehat{g}_2^{(k+1)}, \widehat{\boldsymbol{c}}^{(k)\top} \boldsymbol{B}_m^{(k)}$ to $result_{loss}$

18   Append $\widehat{g}_{1,best}$ to $result_{\widehat{g}_1}$; Append $\widehat{g}_{2,best}$ to $result_{\widehat{g}_2}$

19   break

20   **end**

21   **end**

22   ***Step B: Update linear parameters and B-spline coefficients***

23   Compute $M^{(k+1)} = \Big\{V_i \exp\{\widehat{\boldsymbol{\beta}}^{(k)\top}\boldsymbol{Z}_i + \widehat{g}_1^{(k+1)}(\boldsymbol{X}_i)\}\Big\}_{i=1}^{n_{train}}$; Similar to ***Step* 2**, we obtain the basis functions $\boldsymbol{B}_m^{(k+1)}$; Estimate $(\boldsymbol{\beta}_0, \boldsymbol{\gamma}_0, \boldsymbol{c}_0)$ by optimizing

$$(\widehat{\boldsymbol{\beta}}^{(k+1)}, \widehat{\boldsymbol{\gamma}}^{(k+1)}, \widehat{\boldsymbol{c}}^{(k+1)}) = \arg\min_{(\boldsymbol{\beta}, \boldsymbol{\gamma}, \boldsymbol{c}) \in \mathbb{R}^p \times \mathbb{R}^p \times \mathbb{R}^{q_n}} L_{n_{train}}(\boldsymbol{\beta}, \boldsymbol{\gamma}, \widehat{g}_1^{(k+1)}, \widehat{g}_2^{(k+1)}, \boldsymbol{c}^\top \boldsymbol{B}_m^{(k+1)}).$$

24   **Repeat** the ***Step A*** and ***Step B*** until $\|\widehat{\boldsymbol{\beta}}^{(k+1)} - \widehat{\boldsymbol{\beta}}^{(k)}\| < 0.01$, $\|\widehat{\boldsymbol{\gamma}}^{(k+1)} - \widehat{\boldsymbol{\gamma}}^{(k)}\| < 0.01$, and $\|\widehat{\boldsymbol{c}}^{(k+1)} - \widehat{\boldsymbol{c}}^{(k)}\| < 0.01$.

25 **end**

References

Anthony, M. and Bartlett, P. L. (1999). *Neural network learning: Theoretical foundations*. Cambridge: Cambridge University Press.

Bartlett, P. L., Harvey, N., Liaw, C., and Mehrabian, A. (2019). Nearly-tight vc-dimension and pseudodimension bounds for piecewise linear neural networks. *Journal of Machine Learning Research*, 20(63):1–17.

Bickel, P. J., Klaassen, C. A., Bickel, P. J., Ritov, Y., Klaassen, J., Wellner, J. A., and Ritov, Y. (1993). *Efficient and adaptive estimation for semiparametric models*. Berlin: Springer.

Deng, S., Liu, L., and Zhao, X. (2015). Monotone spline-based least squares estimation for panel count data with informative observation times. *Biometrical Journal*, 57(5):743–765.

Györfi, L., Kohler, M., Krzyzak, A., and Walk, H. (2002). *A distribution-free theory of nonparametric regression*. Berlin: Springer.

Jiao, Y., Shen, G., Lin, Y., and Huang, J. (2023). Deep nonparametric regression on approximate manifolds: Nonasymptotic error bounds with polynomial prefactors. *The Annals of Statistics*, 51(2):691–716.

Kosorok, M. R. (2008). *Introduction to empirical processes and semiparametric inference*. Berlin: Springer.

Lu, M., Zhang, Y., and Huang, J. (2007). Estimation of the mean function with panel count data using monotone polynomial splines. *Biometrika*, 94(3):705–718.

Lu, M., Zhang, Y., and Huang, J. (2009). Semiparametric estimation methods for panel count data using monotone b-splines. *Journal of the American Statistical Association*, 104(487):1060–1070.

Shen, X. and Wong, W. H. (1994). Convergence rate of sieve estimates. *The Annals of Statistics*, 22(2):580–615.

Van der Vaart, A. W. (2000). *Asymptotic statistics*. Cambridge: Cambridge University Press.

Van Der Vaart, A. W. and Wellner, J. A. (1996). *Weak convergence and empirical processes: with applications to statistics*. New York: Springer-Verlag.
